# Engineered bipaternal mice reveal the consequences of life without a maternal genomic contribution

**DOI:** 10.1371/journal.pbio.3003871

**Published:** 2026-06-25

**Authors:** Si-Nan Ma, Fan Li, Yu-Long Zhao, Xue-Han Sun, Xue-Song Chen, Tian-Shi Pan, Qing-Tong Shan, Chao Liu, Gui-Hai Feng, Zhi-Kun Li, Qi Zhou, Wei Li

**Affiliations:** 1 College of Life Sciences, Northeast Agricultural University, Harbin, China; 2 State Key Laboratory of Organ Regeneration and Reconstruction, Institute of Zoology, Chinese Academy of Sciences, Beijing, China; 3 University of Chinese Academy of Sciences, Beijing, China; 4 Beijing Institute for Stem Cell and Regenerative Medicine, Beijing, China; 5 State Key Laboratory of Biocontrol, School of Life Sciences, Sun Yat-sen University, Guangzhou, China; University of Missouri, UNITED STATES OF AMERICA

## Abstract

Successful mammalian development normally requires contributions from both maternal and paternal genomes, yet how these parental components jointly shape organismal development remains incompletely understood. Using engineered bipaternal mice generated from androgenetic embryonic stem cells carrying extensive imprinting-region modifications and produced through tetraploid complementation, we examined developmental and physiological consequences of development supported exclusively by paternal genomes. Placental analyses revealed partial normalization of placental growth but persistent differences among conceptuses. Transcriptomic profiling across embryos and postnatal tissues similarly showed broad alterations in gene expression states involving both imprinted and non-imprinted genes. Despite these differences during development, adult physiology showed a more coherent endpoint: integrated transcriptomic and metabolomic analyses revealed that adult livers converge toward an altered metabolic configuration characterized by coordinated perturbations of the tricarboxylic acid cycle and associated lipid metabolism, accompanied by hepatic lipid accumulation and increased systemic fat mass. These findings indicate that paternal-only mammalian development can proceed across multiple stages but follows altered developmental trajectories that culminate in distinct physiological states, providing insight into how maternal and paternal genomic contributions interact to shape mammalian development and physiology.

## Abstract 2

Successful mammalian development requires both maternal and paternal genomes, but how do these parental components jointly shape the growing organism? This study uses engineered bipaternal mice to reveal the physiological consequences of development supported exclusively by paternal genomes.

## Introduction

Sexual reproduction predominates across complex animals, yet why mammalian development so strongly depends on contributions from both maternal and paternal genomes remains incompletely understood [1–3]. Natural instances of unisexual reproduction highlight this puzzle. In several vertebrate lineages, parthenogenesis can occasionally produce embryos that reach late developmental stages or even hatch, yet developmental success remains sporadic and uneven [4–6]. In birds such as quail, for example, parthenogenetic embryos sharing essentially identical genetic backgrounds can progress to hatching, but a substantial fraction die during late embryogenesis or within days after birth, while a minority survive longer [5,7]. Similar patterns—sporadic success accompanied by pronounced variation in survival—have been reported in other vertebrates capable of facultative parthenogenesis, including reptiles and cartilaginous fishes [4,8]. Across these settings, reduced developmental success is often accompanied by marked differences among individuals that are otherwise genetically similar, suggesting that development supported by a single parental lineage may be less tightly constrained than development supported by contributions from both parents.

Mammalian systems provide an unusually controlled context in which these questions can be examined. Through targeted modification of a limited number of parent-of-origin regulatory loci, embryos carrying genomes derived exclusively from two mothers or two fathers can be experimentally generated in mice [9–11]. Because these engineered embryos can share the same genetic background and the same set of defined genomic modifications, they allow developmental outcomes to be compared under far more controlled conditions than those accessible in naturally occurring unisexual systems. Strikingly, even under these controlled circumstances, developmental outcomes remain highly heterogeneous: individuals that are genetically and experimentally matched can nevertheless differ markedly in whether development proceeds successfully [9–11]. This variability coexists with another recurrent feature of uniparental development—directional biases in organismal growth. Bi-maternal mice tend to remain smaller than wild-type (WT) animals even when birthweight is partially restored, whereas bipaternal mice are markedly larger at birth and continue to follow an elevated postnatal growth trajectory [9–13]. These reciprocal growth tendencies suggest that maternal and paternal genomes may exert distinct—and potentially opposing—influences on developmental regulation, while the persistence of heterogeneous outcomes among matched individuals suggests that the loss of one parental contribution may also permit increased divergence among developmental trajectories.

Although several mechanisms have been proposed to explain the limited success of unisexual reproduction—including increased homozygosity, genome duplication processes associated with automixis, and parent-of-origin regulatory effects on early development—unisexual reproduction itself rarely operates as a regular reproductive program in vertebrates [6,14,15]. In most non-mammalian vertebrates it occurs instead as facultative parthenogenesis under uncommon circumstances, frequently reported in isolated females lacking access to males or in captive populations, and typically associated with low developmental success of the resulting embryos [5,6,16,17]. In mammals, development from a single parental lineage is even more constrained and requires experimental correction of imprinting barriers [9–11]. Yet even under these controlled conditions, individuals that share nearly identical genetic backgrounds and engineered genomic configurations can still diverge markedly in developmental and physiological outcomes [9–13]. This persistence of divergence suggests that biparental development may not only enable development but may also help maintain more reproducible developmental outcomes [15].

Here we investigate this possibility by leveraging engineered bipaternal mice in which extensive modifications at established parent-of-origin regulatory regions enable postnatal survival [11]. Transcriptomic analyses across multiple embryonic and postnatal tissues reveal broad alterations in gene expression organization across organs, indicating that developmental regulation is widely affected in the absence of maternal genomic contribution. At later stages, this altered developmental organization is accompanied by convergence of adult liver physiology toward a distinct state characterized by coordinated alterations in tricarboxylic acid cycle metabolism—an axis central to systemic energy balance and organismal growth [18]. When considered together with the reciprocal growth patterns observed across maternal- and paternal-biased mammalian systems, these findings are consistent with the idea that maternal and paternal genomes exert partially opposing developmental influences whose balance may contribute to the stability of mammalian development [10–13]. Loss of either parental contribution may weaken this balance, whereas their coexistence may help maintain the stability of mammalian developmental and physiological regulation.

### Tetraploid complementation improves the generation of bipaternal mice and reveals early variability in placental development

**Fig 1:**
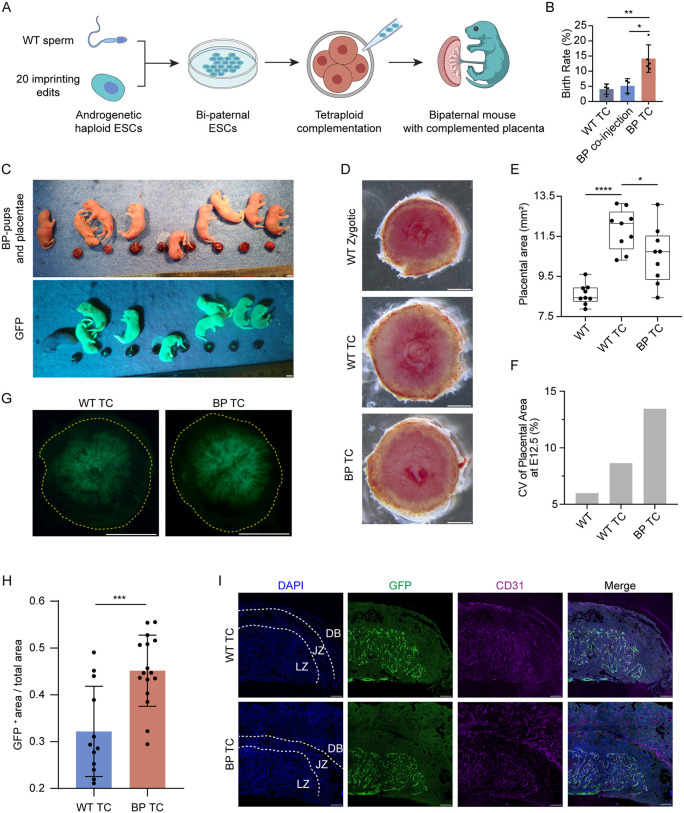
Placental development and GFP contribution in tetraploid-complemented BP conceptuses. **(A)** Schematic illustration of the experimental strategy used to generate BP TC mice. Sperm and androgenetic haploid embryonic stem cells (ahESCs) carrying 20 imprinting modifications were used to generate BP ESCs. These BP ESCs were then introduced into tetraploid embryos via tetraploid complementation. Following embryo transfer, tetraploid-complemented placentas and BP mice were obtained. **(B)** Comparison of birth rates among experimental groups. Bar graph showing the percentage of transferred embryos that yielded live-born pups in the WT TC, BP co-injection, and BP TC groups. Data are presented as mean ± SD, with individual data points representing independent embryo transfer experiments. Statistical significance is indicated. **(C)** Representative images of BP TC pups and placentas. Bright-field (top) and GFP fluorescence (bottom) images of BP TC pups and their corresponding placentas. GFP-positive signals indicate contribution from BP ESCs. The white asterisk marks a control pup lacking GFP fluorescence. Scale bar, 5 mm. **(D)** Representative images of E12.5 placentas derived from WT zygote, WT TC, and BP TC embryos. Scale bar, 1 mm. **(E)** Quantification of placental area at E12.5 for WT, WT TC, and BP TC placentas shown in **(D)**. WT placentas were collected from two naturally pregnant females, whereas WT TC and BP TC placentas were collected from six and eight recipient females, respectively. Each dot represents one placenta. **(F)** Coefficient of variation (CV) of placental area at E12.5 for each group. **(G)** Fluorescence images of the E12.5 placentas shown in D from WT TC and BP TC embryos. Dashed lines outline the placental boundary. Scale bar, 1 mm. **(H)** Quantification of the ratio of GFP-positive area to total placental area for the fluorescence images shown in (G) from WT TC and BP TC placentas. Each dot represents an individual placenta. **(I)** Representative immunofluorescence images of paraffin-embedded placental sections from WT TC and BP TC groups. Sections were stained with DAPI to label nuclei, GFP to detect GFP signal, and CD31 to mark endothelial cells. The placental layers, including the decidua basalis (DB), junctional zone (JZ), and labyrinth zone (LZ), are indicated by dashed lines. Merged images show the spatial distribution of GFP signal relative to the vascular endothelium. Scale bar, 500 μm. Data are mean ± SEM; *p < 0.05, ***p < 0.001, ****p < 0.0001. The data underlying this Figure can be found in S1 Data.

In our previous work, deletions at 20 key imprinted loci enabled the generation of bipaternal (BP) mice by co-injecting androgenetic haploid embryonic stem cells (ahESCs) together with sperm into enucleated oocytes. These modifications comprised 13 imprinting control regions and 7 additional imprinted genes or loci [11]. In that configuration, both embryo and placenta developed under the BP genetic background, and placental abnormalities remained evident even in individuals capable of reaching postnatal stages. To reduce this extraembryonic constraint and establish a more controlled framework for analyzing BP development, we adopted tetraploid complementation (TC), in which ESC-derived cells form the embryo proper while tetraploid cells contribute predominantly to extraembryonic tissues [19]. BP ESC lines carrying the 20 imprinting modifications were first established and subsequently injected into WT tetraploid blastocysts (Fig 1A). Compared with the previously reported BP co-injection strategy, tetraploid complementation significantly increased the birth rate of BP pups, and BP TC also showed a higher birth rate than WT TC (Fig 1B). Pups recovered at birth displayed normal gross morphology and showed uniform, body-wide GFP fluorescence, whereas the corresponding placentas exhibited little detectable GFP signal (Fig 1C).

Previous studies showed that, although fetal weight can be fully restored after imprinting correction, placentas of 20KO BP conceptuses generated by co-injection of sperm and ahESCs remain significantly enlarged relative to WT controls [11]. We found that this placental abnormality was alleviated but not eliminated under tetraploid complementation. When placental weight was normalized to the mean fetal weight of each group, the resulting value decreased progressively with increasing extent of imprinting modification in conceptuses generated by tetraploid complementation, yet remained significantly higher in 20KO BP conceptuses than in WT controls (S1A Fig). To determine whether this residual defect was already evident during early placental development, we next examined placentas at embryonic day 12.5 (E12.5). As commonly observed in ESC-based tetraploid complementation, placentas associated with WT ESC-derived conceptuses were enlarged relative to those from naturally fertilized WT embryos. By comparison, placentas associated with BP ESC-derived conceptuses were smaller than WT ESC TC placentas, although complete normalization was not achieved (Fig 1D and 1E).

Beyond this shift in mean size, the distribution of placental size appeared broader in the BP group. Quantification using the coefficient of variation (CV) showed a tendency toward greater dispersion among placentas associated with BP ESC-derived conceptuses compared with those derived from WT ESC TC embryos, although the difference did not reach statistical significance (Levene’s test, *p* = 0.106) (Fig 1F). Notably, placentas generated from WT ESC TC embryos exhibited relatively limited variability despite also originating from ESC-derived embryos, indicating that the increased variation observed here is unlikely to arise simply from ESC derivation itself.

We also examined whether BP ESCs might contribute directly to placental tissues. Classical studies of uniparental embryos showed that maternal genomes preferentially support embryonic development whereas paternal genomes favor placental growth [2], raising the possibility that androgenetic cells might retain an enhanced capacity to enter placental lineages. Consistent with this possibility, recent work reported that induction of totipotent blastomere-like states in human pluripotent cells was accompanied by upregulation of DIO3, whose mouse ortholog we also found to be elevated in androgenetic samples [10,20].

Scattered GFP-positive ESC-derived cells were detected on the fetal side of placentas from both WT ESC TC and BP ESC TC conceptuses (Fig 1G). Quantitative analysis showed that the GFP-positive area relative to total placental area was significantly higher in placentas associated with BP ESC-derived conceptuses than in those derived from WT ESCs (Fig 1H). However, these GFP-positive cells were localized predominantly within placental vasculature. Immunofluorescence staining revealed strong overlap between GFP signals and the endothelial marker CD31, indicating that ESC-derived cells contributed specifically to endothelial cells within the labyrinth vasculature (Fig 1I). Thus, although BP ESC-derived cells were detectable within placental tissue, their contribution was restricted to fetal endothelial components rather than broadly distributed across trophoblast compartments. Together, these observations indicate that tetraploid complementation substantially improves the recovery of BP conceptuses while minimizing overt extraembryonic failure. Nevertheless, placentas associated with BP embryos still showed incomplete normalization of size and a broader distribution across individuals. These features suggested that developmental outcomes under the BP condition remain heterogeneous, leading us to further examine embryonic development in this system.

### Broader developmental and transcriptional states in BP embryos during mid-gestation

Because placentas associated with BP conceptuses retained residual abnormalities and a broader size distribution even under tetraploid complementation, we next examined whether embryonic development itself exhibited corresponding differences in developmental state. Embryos were therefore analyzed at embryonic day 12.5 (E12.5), a stage at which major organ systems have formed while embryos remain sufficiently comparable for quantitative assessment.

The E12.5 WT TC and BP TC embryos analyzed here were obtained from multiple independent tetraploid-complementation experiments. WT TC embryos were collected from five independent experiments, and BP TC embryos were collected from five additional independent experiments; in each experiment, 60 tetraploid embryos were transferred in total, with 20 embryos transferred into each CD-1 pseudopregnant recipient. Body length was quantified from six embryos per group. WT embryos were naturally conceived embryos collected from two litters, whereas WT TC and BP TC embryos were recovered from four and three recipient females, respectively.

**Fig 2:**
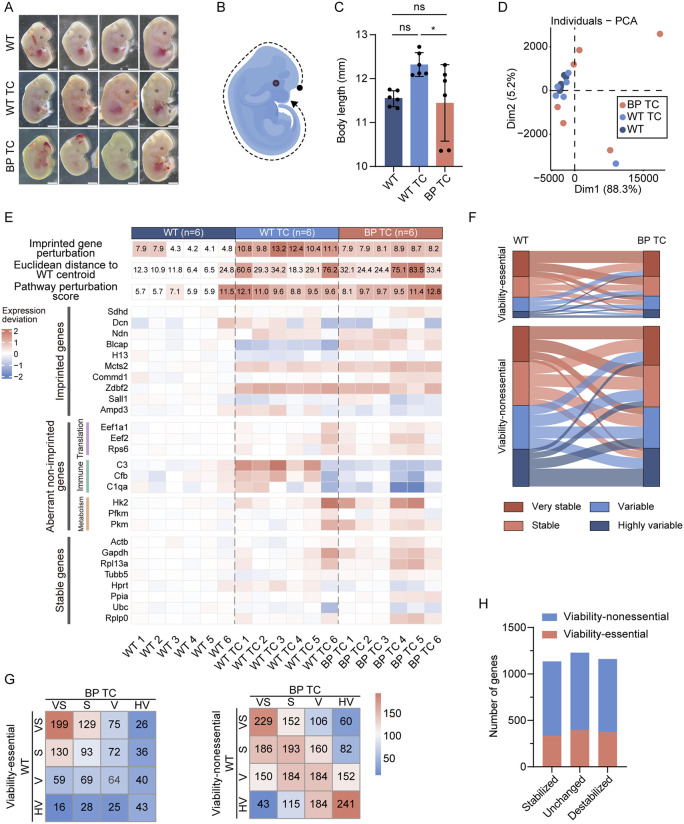
Transcriptome-wide gene expression stability analysis in E12.5 BP TC embryos. **(A)** Representative bright-field images of E12.5 embryos from WT, WT TC, and BP TC groups. Localized hemorrhage in the cranial region was observed in a subset of BP TC embryos, while no obvious hemorrhagic phenotype was detected in WT or WT TC embryos. Scale bar, 1 mm. **(B)** Schematic illustration of the body length measurement. Body length was measured from the forehead to the tip of the tail, following the curvature of the embryonic body along the somite axis. **(C)** Quantification of embryonic body length at E12.5 for WT, WT TC, and BP TC embryos. Each dot represents one embryo. Body-length quantification included six embryos per group. WT embryos were collected from two naturally conceived litters, whereas WT TC and BP TC embryos were obtained from four and three recipient females, respectively, after embryo transfer. **(D)** PCA of RNA-seq transcriptomes from E12.5 embryos in WT, WT TC, and BP TC groups. Each dot represents an individual embryo (*n* = 6 per group). Dim1 and Dim2 account for 88.3% and 5.2% of the total variance, respectively. Samples are colored according to genotype as indicated. **(E)** Integrated expression perturbation map across individual E12.5 embryos. Upper tracks show sample-level imprinted-gene, WT-centroid distance, and pathway perturbation scores for WT, WT TC, and BP TC embryos. The heatmap shows WT-centered expression deviations for selected imprinted genes, representative aberrant non-imprinted genes related to translation, immune/complement, and metabolism, and stable non-imprinted genes. Red and blue indicate increased and decreased expression relative to the WT mean, respectively. **(F)** Sankey diagrams illustrating transitions in gene expression stability between WT and BP TC embryos for viability-essential (top) and viability-nonessential (bottom) gene sets. Genes were classified into four stability categories (very stable, stable, variable, and highly variable) based on the 25%, 50%, and 75% percentiles of the CV distribution shown in S1E Fig. Flows represent the proportion of genes transitioning between stability states from WT to BP TC. **(G)** Heatmaps showing the number of genes undergoing transitions between expression stability states from WT to BP TC embryos for viability-essential (top) and viability-nonessential (bottom) gene sets. Stability categories (very stable [VS], stable [S], variable [V], and highly variable [HV]) were defined based on the 25%, 50%, and 75% percentiles of the CV distributions shown in S1E Fig. Rows indicate stability states in WT embryos, and columns indicate stability states in BP TC embryos. Numbers within each cell represent gene counts. **(H)** Numbers of stabilized, unchanged, and destabilized genes in BP TC embryos relative to WT, stratified by viability-essential and viability-nonessential gene sets. Gene stability categories were defined based on CV thresholds shown in S1E Fig. Bars represent gene counts. Data are mean ± SEM; *p < 0.05, **p < 0.01, ns, not significant. The data underlying this Figure can be found in S2 Data.

Direct measurement of embryo body length was performed from bright-field images using the somite-aligned approach shown in Fig 2A and 2B. BP TC embryos showed a modest reduction in body length and a broader observed range compared with WT embryos, consistent with heterogeneous developmental progression at this stage. In contrast, WT TC embryos displayed body lengths and distributions broadly comparable to naturally conceived WT embryos. Because these embryos were collected from multiple litters or recipient females, the body-length distribution is presented as a developmental-state observation within the current experimental design rather than as evidence that length variation arises solely from intrinsic BP-associated developmental variability (Fig 2C).

To characterize transcriptional states in these embryos, we performed whole-embryo RNA-seq on WT, WT TC, and BP TC embryos at E12.5, using six biological replicates per group. Principal component analysis (PCA) showed tissue relationships among WT, WT TC, and BP TC embryos at the whole-embryo level. WT TC embryos overlapped with WT embryos but also showed measurable individual-level deviations, whereas BP TC embryos included samples with stronger deviations in transcriptional space (Fig 2D). Pairwise distance and correlation analyses further indicated that TC-derived embryos should be interpreted in relation to both naturally conceived WT embryos and the WT TC procedural baseline (S1B and S1C Fig).

Differential expression analysis followed by gene set enrichment analysis identified alterations in pathways related to core cellular and metabolic processes, including protein translation, complement activation, and glucose metabolism (S1D Fig). Because individual pathway-module plots alone do not establish a greater BP TC range than WT TC, we used these analyses to identify representative perturbed processes and incorporated them into the sample-level perturbation framework described below.

To further examine how transcriptional perturbation was distributed across individual embryos, we integrated sample-level perturbation scores with expression deviations of selected gene classes (Fig 2E). WT TC embryos already showed measurable deviation from naturally conceived WT embryos, particularly in imprinted-gene and pathway-level perturbation scores, indicating that ESC-derived tetraploid complementation establishes a detectable transcriptional baseline. BP TC embryos shared this baseline but showed additional or stronger deviations across imprinted genes and representative non-imprinted gene modules. In contrast, a set of stable non-imprinted genes remained largely unchanged across groups, indicating that the observed perturbation was structured rather than uniformly genome-wide. These results support the interpretation that BP TC embryos exhibit incomplete normalization within an ESC-derived developmental background.

To further quantify transcriptional variability, we calculated the CV for all expressed genes within each group. BP TC embryos showed the most right-shifted overall CV distribution in this analysis, indicating an expansion in the range of gene expression levels across embryos. WT TC embryos also showed a rightward shift relative to WT embryos, consistent with a measurable transcriptional effect of the ESC-derived tetraploid-complementation procedure itself (S1E Fig). Sankey analysis revealed that a subset of genes shifted stability categories in both WT TC and BP TC embryos, suggesting effects of tetraploid complementation and altered imprinting states on gene expression stability (S1F Fig). The observed dispersion was not explained by differences in sequencing depth among samples, as illustrated by representative analyses of several highly variable genes (S2 Fig).

**Fig 3:**
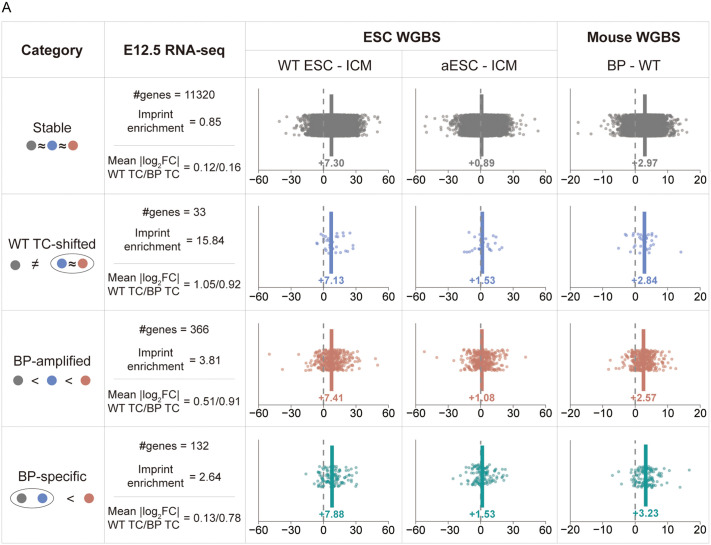
RNA-seq-defined gene classes are associated with methylation features in ESCs and mouse WGBS datasets. **(A)** Integrated summary of E12.5 RNA-seq-defined gene classes and corresponding WGBS methylation features. Genes were classified as stable, WT TC-shifted, BP-amplified, or BP-specific according to expression patterns across WT, WT TC, and BP TC embryos. In the category schematics, gray, blue, and red indicate WT, WT TC, and BP TC, respectively. The RNA-seq summary shows gene number, imprinted gene enrichment, and mean absolute log2 fold-change relative to WT. Dot-strip plots show ΔmCG for gene-associated regions in WT ESC vs. ICM, aESC vs. ICM, and BP vs. WT mouse WGBS comparisons. Each dot represents one gene-associated region. Solid vertical lines indicate median ΔmCG; dashed lines indicate ΔmCG = 0. The data underlying this Figure can be found in S3 Data.

The observation that some stability-state transitions were shared by WT TC and BP TC embryos indicated that the E12.5 transcriptomic differences included both a procedure-associated component and BP-associated components. To separate these patterns more explicitly and to link the embryonic transcriptome to methylome features at other levels of the system, we classified E12.5 expressed genes into stable, WT TC-shifted, BP-amplified, and BP-specific groups according to their expression patterns across WT, WT TC, and BP TC embryos (Fig 3A). The WT TC-shifted class captured genes concordantly shifted in WT TC and BP TC embryos relative to WT; this class was small but strongly enriched for imprinted genes and showed methylation differences in WT ESCs relative to ICM, consistent with the sensitivity of these loci to the ESC-derived experimental context. The BP-amplified and BP-specific classes identified genes with additional BP-associated deviation, and these classes showed corresponding methylation features in aESC/ICM and BP-WT mouse WGBS comparisons. Although these comparisons do not establish direct methylation inheritance across stages, they provide cross-stage methylome context for the E12.5 RNA-seq-defined gene classes. This framework also provided a basis for examining how BP-associated transcriptional and methylation states are represented across mouse tissues.

To examine whether these transcriptional changes also involved genes important for embryonic development, we analyzed the expression of genes associated with embryo lethality, abnormal survival, or premature death [21]. BP TC embryos exhibited stronger dysregulation of essential genes than WT TC embryos, and these genes were linked to functions including neural development, heart development, and growth regulation (Fig 2F–2H). A subset of canonical imprinted genes also remained dysregulated in BP TC embryos (S1G Fig), providing a direct molecular connection to the imprinting perturbations engineered in BP ESCs. Together, these analyses indicate that by mid-gestation BP embryos occupy altered developmental and transcriptional states relative to control embryos. Even under tetraploid complementation, which largely alleviates extraembryonic constraints, embryonic development under the BP condition remains incompletely normalized.

### Organ-specific transcriptional organization across multiple tissues in BP mice

**Fig 4:**
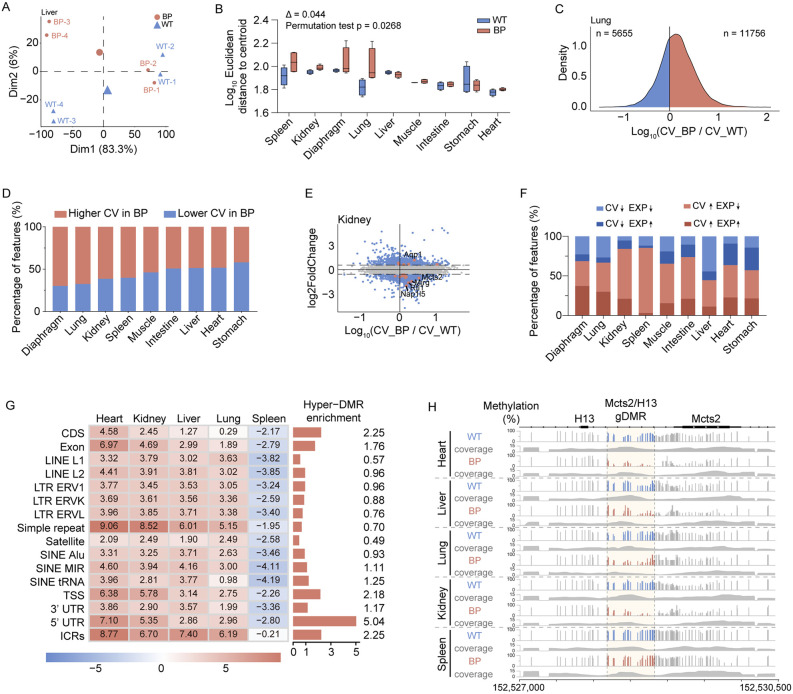
Multi-organ transcriptomic organization and DNA methylation profiles in BP mice. **(A)** PCA of RNA-seq transcriptomes from liver tissues of WT and BP mice collected at term. Each dot represents an individual biological replicate (*n* = 4 per group). Samples are colored and labeled according to genotype. Dim1 and Dim2 account for 55.6% and 32.1% of the total variance, respectively. Samples are colored according to genotype as indicated. PCA results from additional organs are shown in S3A Fig. **(B)** Quantification of Euclidean distances to the group centroid in PCA space for nine organs collected at term from WT and BP mice. Distances were calculated for each sample based on the corresponding organ-specific PCA analyses. The difference in mean distances between BP and WT groups (Δ = 0.044) and the permutation test p value (*p* = 0.0268) are indicated. Boxes indicate the median and interquartile range. **(C)** Probability density distribution of log10(CV_BP/ CV_WT) for all expressed genes in the lung. Gene-wise CV values were calculated across four biological replicates for each genotype. Positive values indicate higher expression variability in BP relative to WT, whereas negative values indicate lower variability. Numbers indicate the total genes included. Corresponding distributions for other organs are shown in S3B Fig. **(D)** Percentage of genes showing higher or lower expression variability in BP relative to WT across nine organs, based on the CV ratio analysis shown in C and S3B Fig. Genes with log10(CV_BP/CV_WT) > 0 were classified as having higher CV in BP, whereas genes with log10(CV_BP/CV_WT) < 0 were classified as having lower CV in BP. **(E)** Scatter plot showing the relationship between gene expression fold change (log2 fold change) and expression variability change (log10(CV_BP/CV_WT)) in the kidney. Each dot represents an individual differentially expressed gene. Imprinted genes with higher CV in BP are highlighted and labeled. The dashed line represents the threshold for differential expression (log2 fold change ≥ 1.5), and the full horizontal line indicates the threshold for higher CV in BP relative to WT (log10(CV_BP/CV_WT) > 0). Corresponding plots for other organs are shown in S4A Fig. **(F)** Proportional distribution of genes classified into four categories based on changes in expression variability (CV) and expression level in BP relative to WT across different organs. Genes were grouped as CV↓/EXP↓, CV↓/EXP↑, CV↑/EXP↓, or CV↑/EXP↑, according to the direction of change in log10(CV_BP/CV_WT) and log2 fold change shown in E. Stacked bars represent the percentage of genes in each category for the indicated organ. **(G)** Element-level DNA methylation differences and BP-hypermethylated DMR enrichment across five mouse tissues. The heatmap shows BP minus WT ΔmCG across genomic features including coding regions, exons, transposable element subclasses, TSS, UTRs, and ICRs/gDMRs. The bar plot shows enrichment of merged BP-hypermethylated DMRs in each genomic feature. Positive ΔmCG values indicate higher methylation in BP. **(H)** Locus-specific methylation profiles at the Mcts2/H13 gDMR across five mouse tissues. The methylation scale ranges from 0% to 100%. The highlighted interval indicates the Mcts2/H13 gDMR, redefined according to the public parental methylome annotation. Coverage tracks are shown below the methylation profiles to distinguish low methylation from missing or low-coverage CpG sites. The data underlying this Figure can be found in S4 Data.

To examine how developmental differences observed during embryogenesis are reflected at the organismal level, we analyzed transcriptional states across multiple organs in BP and WT mice at term. Bulk RNA sequencing was performed on nine tissues collected from BP and WT animals. The term BP mice used for the additional nine-organ collection were generated from three independent tetraploid-complementation experiments, each involving transfer of 60 tetraploid embryos in total, with 20 embryos transferred into each CD-1 pseudopregnant recipient. PCA showed tissue-dependent separation between WT and BP samples, with the extent and direction of separation differing among organs (Figs 4A and S3A).

Quantification of distances to group centroids showed a tendency toward increased dispersion in BP samples in several organs, and an across-organ permutation analysis indicated a modest overall increase in BP within-group dispersion relative to WT (Fig 4B). These analyses suggest altered transcriptional organization across BP tissues, although individual tissue-level dispersion patterns should be interpreted cautiously given the limited number of biological replicates.

At the gene-expression level, CV-ratio and differential-expression analyses provided gene-level views of these tissue-associated transcriptional patterns. In several organs, a substantial fraction of expressed genes showed higher CV in BP than in WT, whereas other genes showed lower CV, indicating that BP tissues do not display a uniform genome-wide increase in variability (Figs 4C, 4D, and S3B). Genes contributing to transcriptional differences between BP and WT animals were then examined together with their CV changes (Fig 4E and 4F).

Because BP mice were generated through manipulation of genomic imprinting, we next examined transcriptional patterns associated with imprinted loci. Imprinted genes that remained dysregulated in BP mice generally displayed broader expression ranges relative to WT samples across several organs (Figs 4E and S4A). Consistent with this observation, analyses linking non-imprinted gene expression with an imprinting-associated transcriptional module suggested that imprinting-related dysregulation may be associated with broader regulatory programs across tissues (S4B Fig).

To evaluate whether the observed patterns could be explained by technical variation, we performed technical replicate analyses by splitting RNA samples prior to library construction and sequencing. Variation between technical replicates was substantially lower than that observed between biological samples (S5 Fig), supporting the interpretation that the transcriptional differences observed across BP tissues primarily reflect biological differences among individuals. These technical replicates help exclude library construction or sequencing noise as the main explanation, although they do not replace larger biological cohorts for estimating tissue-level variance.

Finally, because genomic imprinting is closely associated with DNA methylation, we analyzed mouse tissue WGBS data from WT and BP mice to examine BP-associated methylation patterns across multiple organs. To resolve the sequence contexts underlying the BP-associated methylation changes, we stratified BP-WT differences across major genomic annotations, including coding and exonic regions, repetitive elements, UTR/TSS-associated regulatory regions, and imprinted control regions/germline DMRs (Fig 4G). These annotations are partially overlapping and provide sequence-context information rather than an exhaustive non-overlapping partition of the genome. This analysis revealed that methylation differences were organized by both genomic context and tissue type. Several genic and regulatory annotations, including 5′ UTR, TSS-associated regions and ICRs/gDMRs, showed prominent BP-associated hypermethylation or hyper-DMR enrichment, whereas other repeat and intergenic classes showed more variable or tissue-dependent patterns. Sample-level global mCG values are provided as a whole-genome overview in S6A Fig, and locus-level methylation and coverage at the Mcts2/H13 gDMR are shown in Fig 4H. Together, these results provide a more resolved epigenetic framework for interpreting the postnatal transcriptional abnormalities observed in BP tissues.

### Metabolic alterations in adult BP liver reveal convergence toward a distinct physiological state

To examine how developmental divergence observed earlier in BP mice is reflected at later physiological stages, we analyzed liver metabolism in adult animals. Because the liver integrates major pathways of systemic energy metabolism—including carbohydrate utilization, mitochondrial energy production, and lipid metabolism—we focused on this organ to assess whether developmental differences are accompanied by metabolic changes in adulthood [22].

**Fig 5:**
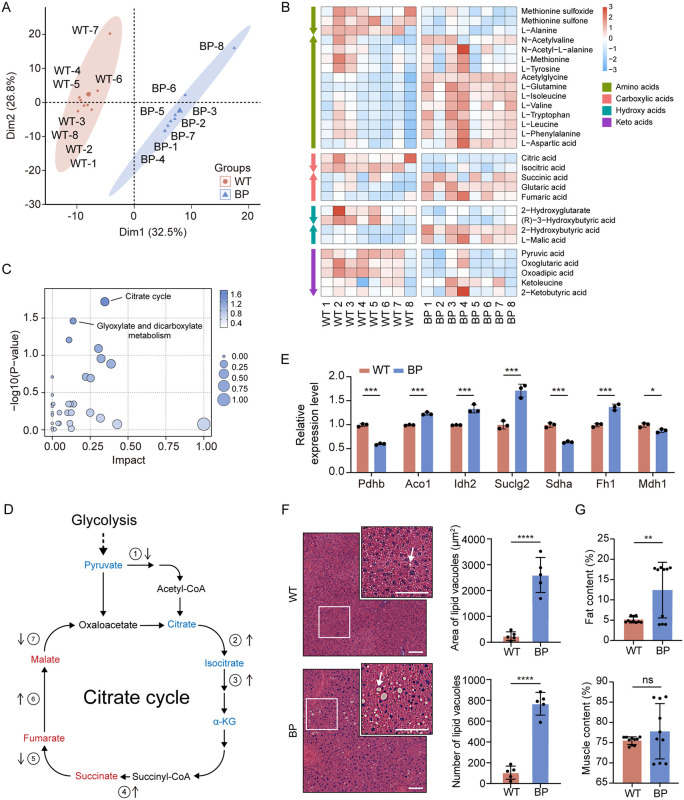
Integrated transcriptomic and metabolomic analyses identify alterations in TCA cycle activity and lipid metabolism in BP mice. **(A)** PCA of metabolomic profiles from WT and BP samples. Each dot represents an individual biological replicate. WT and BP samples show separation along the principal components. Dim1 and Dim2 account for 32.5% and 26.8% of the total variance, respectively. **(B)** Heatmap illustrating differential metabolite profiles between wild-type (WT) and BP samples. Metabolite abundances were scaled and visualized across individual biological replicates, with colors representing relative levels (red, higher abundance; blue, lower abundance). Upward and downward arrows indicate metabolite up-regulation and down-regulation, respectively. Metabolites are grouped by biochemical class, including amino acids, carboxylic acids, hydroxy acids, and keto acids. Samples are arranged by genotype, with separate columns for BP and WT replicates. **(C)** Integrated pathway enrichment analysis of metabolomic and transcriptomic data. Bubble plot showing significantly enriched metabolic pathways identified by joint analysis of metabolite abundance and gene expression changes. The x-axis represents pathway impact, and the y-axis indicates −log10(*P*-value). Bubble size reflects the proportion of matched features, and color intensity corresponds to pathway impact. **(D)** Schematic overview of the TCA cycle highlighting metabolic steps altered based on integrated transcriptomic and metabolomic analyses. Metabolites and enzymatic steps showing increased or decreased abundance or activity are indicated by upward or downward arrows, respectively. Numbers denote individual reaction steps within the TCA cycle. **(E)** Quantitative RT–PCR analysis of key genes encoding enzymes of the TCA cycle. Relative expression levels of *Pdhb*, *Aco1*, *Idh2*, *Suclg2*, *Sdha*, *Fh1*, and *Mdh1* were measured to validate the involvement of the TCA cycle identified by integrated transcriptomic and metabolomic analyses. Data are normalized to WT controls. **(F)** Representative hematoxylin and eosin (H&E)-stained liver sections from WT and BP mice at term. Boxed regions are shown at higher magnification to highlight lipid vacuole-like structures (arrows). Quantification of lipid vacuole area (top right) and number (bottom right) is shown. Each dot represents one randomly selected, non-overlapping microscopic field sampled from different section levels of the same liver, chosen to provide broader coverage of the tissue. **(G)** Body composition analysis of adult WT and BP mice at 16 weeks of age, showing fat mass and muscle mass as measured by the body composition analyzer. These parameters are presented separately and do not sum to total body composition, which also includes additional compartments such as free water. Each dot represents one biological replicate, and bars represent mean ± SEM. Statistical significance is indicated as *P < 0.05, **P < 0.01, ***P < 0.001, ****P < 0.0001; ns, not significant. The data underlying this Figure can be found in S5 Data.

Untargeted metabolomic profiling of adult liver tissues revealed a clear separation between BP and WT samples in PCA, indicating substantial differences in global metabolic states between the two groups (Fig 5A). Notably, in contrast to the transcriptional dispersion observed during earlier developmental stages, metabolomic profiles of adult BP livers formed a relatively compact cluster. This pattern suggests convergence toward a distinct metabolic state rather than persistent metabolic dispersion.

A heatmap of differential metabolites showed broad metabolic remodeling in BP liver (Fig 5B). Pathway analysis identified enrichment of amino acid, carbohydrate, nucleotide, and lipid metabolic pathways (S7A Fig). Notably, tricarboxylic acid (TCA) cycle-related metabolites were altered in BP liver, including changes in citrate, *cis-*aconitate, succinate, and fumarate (Figs 5C and S7B). Integration with liver transcriptomic data further showed dysregulation of genes involved in mitochondrial metabolism and the TCA cycle (Fig 5D). Quantitative PCR confirmed altered expression of representative TCA-associated genes (Fig 5E), consistent with disruption of mitochondrial metabolic programs in BP liver (S7C Fig) [23].

Histological examination of liver tissue revealed abundant lipid vacuoles within hepatocytes of BP mice that were largely absent in WT controls (Fig 5F, white arrows). Quantitative analysis confirmed a marked increase in both the number and area of lipid vacuoles in BP livers, indicating substantial lipid accumulation.

To evaluate whether these hepatic differences were accompanied by broader physiological changes, body composition was analyzed using nuclear magnetic resonance. Adult BP mice exhibited a significant increase in total fat mass compared with WT animals, whereas lean mass remained largely unchanged (Fig 5G). Inter-individual variation in fat mass was also detectable among BP mice. In addition, skeletal abnormalities, including spinal curvature, were observed in adult BP mice (S7D Fig), indicating that physiological differences extend beyond hepatic metabolism. Taken together, these observations indicate that although developmental trajectories in BP mice display increased divergence during earlier stages, adult physiology can converge toward a distinct metabolic state centered on hepatic energy metabolism. The metabolic differences observed in adult BP livers are accompanied by hepatic lipid accumulation and altered systemic fat composition, suggesting that developmental differences associated with parental genome imbalance may ultimately be reflected in physiological states in adulthood. Consistent with this interpretation, BP mice have previously been reported to exhibit accelerated postnatal growth and shortened life span, organismal traits broadly compatible with differences in systemic metabolic regulation [11].

## Discussion

The present study provides a multi-level view of mammalian development supported exclusively by paternal genomes. Across placental development, mid-gestation embryos, postnatal tissues, and adult physiology, BP conceptuses and animals do not exhibit a single uniform defect. Instead, they show altered developmental and transcriptional organization, including broader distributions in several analyses. At the same time, adult liver physiology converges toward a relatively compact but shifted metabolic configuration characterized by coordinated alterations in tricarboxylic acid cycle metabolism and lipid homeostasis. Together, these observations indicate that paternal-only development alters not only developmental outcomes but also the overall organization of developmental progression across individuals.

One interpretation consistent with these findings is that maternal and paternal genomes normally provide complementary influences that help stabilize mammalian development [1,2]. Reciprocal growth tendencies in uniparental systems support this view: bi-maternal animals tend toward reduced growth, whereas BP animals display overgrowth phenotypes, observations broadly consistent with kinship-based models of parental conflict [9,11]. At the same time, however, both bi-maternal and BP mouse systems retain residual abnormalities in imprinted gene regulation [11,12,15]. The present findings therefore cannot determine whether the developmental dispersion observed here arises entirely from incomplete normalization of imprinting or also reflects additional parental differences. What they do show is that even after extensive correction of known imprinting barriers, development supported by paternal genomes alone remains less tightly constrained [10,11].

A similar pattern of uneven developmental outcomes appears in vertebrates capable of unisexual reproduction [5,6]. In birds, reptiles, and cartilaginous fishes, offspring derived from a single parental lineage can occasionally develop to advanced stages, yet survival and developmental success vary markedly among individuals despite apparently similar genetic origins [4,5,8]. Because these systems lack the canonical imprinting architecture characteristic of mammals, such observations indicate that developmental instability in uniparental reproduction cannot be interpreted solely through classical mammalian imprinting [5,6,15]. Although classical genomic imprinting is largely restricted to mammals, parental contributions in vertebrates may still differ in ways that influence developmental outcomes across species. Instead, they point more broadly to functional differences between sperm- and oocyte-derived contributions that may influence developmental processes [24,25]. In this context, mammalian imprinting may represent one particularly stable molecular realization of parental asymmetry [15].

Maternal and paternal genomes enter embryogenesis in distinct regulatory states shaped by gamete-specific chromatin organization and epigenetic remodeling histories [24,25]. Some of these asymmetries are transient, yet transient regulatory differences can still influence later development. For example, loci such as Zdbf2 involve early parent-specific regulatory events that subsequently establish secondary epigenetic states affecting postnatal physiology, and maternally inherited H3K27me3 can transiently mediate parent-of-origin gene expression during early development [26,27]. These examples illustrate how early parental differences could shape developmental trajectories even when their initial molecular signatures are later remodeled.

Several limitations should nevertheless be considered. ESC derivation and tetraploid complementation introduce a shared procedural background, and residual dysregulation of imprinted genes remains detectable in BP tissues. WT TC embryos therefore provide an important procedure-matched baseline at E12.5; at the same time, the methylome and transcriptional analyses in Fig 3 indicate that WT ESC-derived states already carry measurable imprinting-related perturbations, while naturally conceived WT animals remain the physiological reference for postnatal endpoints. The absence of a postnatal WT TC cohort therefore limits fully procedure-matched interpretation of tissue-level BP–WT comparisons, but does not diminish their value as postnatal manifestations of development supported by paternal genomes alone. The limited scale of the RNA-seq and WGBS datasets also supports context-resolved analyses rather than definitive tissue-by-tissue variance estimates. Despite these constraints, engineered BP systems remain uniquely informative for examining mammalian development in the complete absence of maternal genomic contribution and for probing how parental genome imbalance shapes postnatal regulatory and physiological states.

Taken together, these observations support a view in which maternal and paternal genomes help constrain mammalian development within a relatively narrow range of trajectories. When development proceeds with paternal genomes alone, this constraint may be weakened, allowing developmental states to deviate more readily before adult physiology stabilizes in an altered metabolic configuration. More broadly, the BP model highlights how maternal and paternal genomic contributions jointly shape mammalian development across embryonic, postnatal, and physiological levels.

## Materials and methods

### Ethics statement

All animal experiments in this study were reviewed and approved by the Institutional Animal Care and Use Committee of the Institute of Zoology, Chinese Academy of Sciences (IOZ, CAS). No specific approval number was issued; approval was granted by the named committee. All mice were maintained under SPF conditions in the animal facilities of IOZ, CAS, and all procedures were performed in accordance with the Guidelines for the Care and Use of Laboratory Animals of IOZ, CAS. The 2-cell-stage embryos used for electrofusion and the pseudopregnant female mice were CD-1 mice provided by Beijing Vital River Laboratories.

### Cultivation of 20ko-androgenetic diploid ESCs

The androgenetic diploid embryonic stem cells (adESC) were derived as previously described [11]. Briefly, RFP-positive sperm of adult male mice were injected into MII oocytes by intracytoplasmic sperm injection (ICSI). Then the G0/G1 stage GFP-positive ahESCs carrying 20 imprinted-region deletions were injected into the reconstructed embryos to generate androgenetic diploid embryos. Derived embryos were activated by SrCl2 (10 mM) in calcium-free CZB medium containing cytochalasin B (5 mg/mL) for 4 hours, and further cultured in CZB for 4.5 days, 37 °C/5% CO_2_. Blastocysts with GFP and RFP were used to establish the androgenetic diploid ESCs (adESCs). The 20ko-adESC lines were cultured in the 2i medium plus 5% knockout serum replacement (Gibco). The 2i medium consists of N2B27 medium supplemented with 1 μM MEK inhibitor PD0325901 (Stemgent), 3 μM GSK3β inhibitor CHIR99021 (Stemgent), and mouse recombinant LIF (Millipore).

### Tetraploid embryo complementation

The procedure for the tetraploid complementation experiment followed previously reported methods [19]. CD-1 female mice were superovulated by intraperitoneal injection of PMSG, followed 48 hours later by HCG injection. The females were then mated with CD-1 males, and 2-cell stage embryos were collected on the following day. These embryos were subjected to electrofusion to generate tetraploid embryos, which were then cultured in CZB medium until the 4-cell stage. Chimeric experiments were subsequently performed, followed by embryo transfer. The chimeric embryos were transplanted into the oviducts of CD-1 E0.5 pseudopregnant mice. Subsequently, male mice were obtained on gestational day E19.5. For all tetraploid complementation experiments, 20 tetraploid embryos were transferred into each CD-1 pseudopregnant female. The WT TC experiments used to collect E12.5 embryos were performed five independent times, with 60 tetraploid embryos transferred per experiment. The BP TC experiments used to collect additional E12.5 embryos were also performed five independent times, with 60 tetraploid embryos transferred per experiment. The BP TC experiments used to generate additional term mice for nine-organ collection were performed three independent times, with 60 tetraploid embryos transferred per experiment.

### Mouse strains and ESC lines

All mice used in this study were maintained under specific pathogen-free conditions with a 12-hour light/12-hour dark cycle and free access to food and water. C57BL/6J mice were used as the genetic background for naturally conceived WT embryos, WT ESCs, BP ESCs, and sperm donors unless otherwise stated. WT embryos used in this study were obtained by in vivo fertilization using C57BL/6J mice. The WT ESC line used for tetraploid complementation was an unmanipulated diploid biparental ESC line derived from naturally fertilized C57BL/6J-GFP embryos cultured to the blastocyst stage. These WT ESCs were conventional fertilization-derived diploid ESCs, not diploid androgenetic ESCs. BP ESCs were androgenetic diploid ESCs carrying 20 imprinting-control-region deletions, established as described above and in our previous study [11]. CD-1 females were used as pseudopregnant recipients for embryo transfer.

### Sample collection and processing

For whole-embryo RNA-seq analysis: WT, WT TC, and BP TC embryos were harvested at embryonic day 12.5 (E12.5). Following dissection from the uterus in ice-cold PBS, each whole embryo was rinsed briefly to remove maternal blood contaminants and immediately snap-frozen in liquid nitrogen as a single intact sample. For placental area quantification at E12.5, WT placentas were collected from two naturally pregnant females, whereas WT TC and BP TC placentas were collected from six and eight recipient females, respectively. For embryo body-length analysis, six E12.5 embryos were quantified per group. WT embryos were naturally conceived embryos collected from two litters. WT TC embryos were obtained from four recipient females following embryo transfer, and BP TC embryos were obtained from three recipient females following embryo transfer. Each embryo was photographed individually, and body length was measured from bright-field images using the somite-aligned measurement strategy shown in Fig 2B. Frozen embryos were stored in liquid nitrogen until RNA extraction and sequencing library preparation. For tissue-specific RNA-seq analysis: WT and BP mice were collected at term. Each organ was harvested using standard dissection techniques, ensuring removal of surrounding connective tissues, and immediately snap-frozen in liquid nitrogen to preserve nucleic acid integrity. All tissue samples were stored in liquid nitrogen prior to further processing. For both sample types, frozen tissues were maintained on dry ice during transport to the sequencing facility to prevent any thawing or degradation. Embryonic analyses included naturally conceived WT embryos, ESC-derived WT TC embryos, and BP TC embryos, whereas postnatal tissue RNA-seq analyses included WT and BP mice.

### Quantitative real-time PCR

Total RNA (1 µg) was reverse transcribed using the HiScript III 1st Strand cDNA Synthesis Kit (Vazyme). Quantitative PCR was performed using the QuantStudio 6 Flex Real-Time PCR System (Applied Biosystems). Relative expression levels were calculated using the 2−ΔΔCt method with Gapdh as the internal control.

### Histological section analyses

Liver tissues from BP or WT mice were fixed in 4% PFA for overnight, and embedded in paraffin and sectioned into 4 μm thick sections. After being baked at 37 °C, all samples were sliced at room temperature. Sections were deparaffinized in xylene (2 × 5 min), and rehydrated with successive 1-min washes in 100%, 96%, 80%, and 70% EtOH. After that, sections were stained with hematoxylin for 2 min and rinsed in distilled water, 0.1% hydrochloric acid in 50% EtOH, and distilled water for 15 min. Following that, the samples were stained with eosin for 1 min before being rinsed in distilled water. The slides were dehydrated with 95% and 100% EtOH, dehydrated with xylene (2 × 5 min), and mounted with coverslips. All slides were photographed under Aperio VERSA Brightfield, Fluorescence & FISH Digital Pathology Scanner (Leica).

### Immunofluorescence of placental sections

Placentae from E12.5 embryo were fixed in 4% paraformaldehyde, embedded in paraffin, and sectioned into 5 μm-thick serial sections. Antigen retrieval was performed using standard protocols, followed by three washes in PBS. Sections were blocked with 1% BSA in PBS at room temperature for 1 hour and then incubated overnight at 4 °C with anti-GFP and anti-CD31 monoclonal antibody (Abcam) diluted in the blocking solution. After washing three times in PBS, the sections were incubated with Alexa Fluor 488 donkey anti-chicken and Alexa Fluor 647 donkey anti-rabbit secondary antibody (Invitrogen) for 1 hour at room temperature. Nuclei were counterstained with DAPI for 10 min following an additional three PBS washes. Images were acquired using a Dragonfly 200 imaging system (Andor).

### NMR-based body composition analysis

Body composition analysis was performed using quantitative magnetic resonance (QMR) imaging with a body composition analyzer for conscious small animals (QMR06-060H-PRO, NIUMAG, China). Adult mice at 16 weeks of age were analyzed without anesthesia. The relative contents of fat mass, muscle mass (lean mass), and free water were directly measured for BP and WT mice according to the manufacturer’s instructions. Body composition parameters were expressed as percentages of total body weight. Each mouse was measured individually and the values were recorded for subsequent analysis.

### Mouse tissue WGBS data and analysis

Mouse tissue WGBS data analyzed here comprise five organs of WT and BP mice as described previously [11]. In the present study, these datasets were reanalyzed to assess feature-level methylation differences, BP-hypermethylated DMR enrichment, and locus-level methylation patterns. Briefly, genomic DNA (1 μg) was extracted using phenol: chloroform: isoamyl alcohol (25:24:1) and used for library preparation with the EZ DNA Methylation-Gold Kit (Zymo Research, Cat# D5006). WGBS libraries were constructed through the following steps: DNA fragmentation, end repair, dA-tailing, ligation of methylated adapters, size selection of 350–500 bp fragments, bisulfite conversion, and PCR amplification. Library quality was assessed using a Qubit 3.0 Fluorometer (Life Technologies) for quantification and an Agilent 2100 Bioanalyzer (Agilent Technologies) for fragment size distribution. Libraries were sequenced on an Illumina HiSeq X10 platform to generate 150-bp paired-end reads.

### WGBS-seq analysis

Whole-genome methylation sequencing data were aligned to the mouse GRCm39 reference genome using Bismark [28]. After deduplication of the BAM files, methylation sites were extracted, and CpG methylation levels were calculated as the proportion of methylated reads among total reads covering each CpG site. Global methylation levels were calculated as the mean methylation level across all covered CpG sites. Differentially methylated regions were identified by comparing BP and WT samples within each tissue. Genomic feature annotations, including coding sequence, exons, transposable-element subclasses, TSSs, UTRs and imprinted control regions/germline DMRs, were used to summarize feature-level methylation differences and DMR enrichment. CpG methylation and coverage tracks at the Mcts2/H13 gDMR were generated from Bismark CpG methylation coverage files. The analyzed Mcts2/H13 gDMR interval corresponded to chr2:152528194-152528847 in GRCm39 and was redefined according to the public parental methylome annotation; the displayed genomic window was extended to include the surrounding H13 and Mcts2 loci. For each organ and genotype, CpG methylation levels were summarized from two biological replicates. Methylation level was displayed as vertical bars on a 0%–100% scale, and coverage was calculated as the total number of methylated plus unmethylated reads covering each CpG site. Coverage values were capped for visualization to avoid domination by isolated high-depth CpG sites. Quality control metrics including sequencing depth, mapping efficiency, and CpG coverage for each sample are provided in S2 Table.

### Gene-associated methylation analysis using public ESC, aESC, ICM, and mouse WGBS datasets

For the comparison shown in Fig 3, ES-f3 (GSM4603224, passage 8) was used as the WT ESC sample, and aESC methylation was calculated as the mean of aES2 and aES3 (GSM4603222 and GSM4603221, both passage 8). The ICM methylation dataset was GSM1386023 (ICM_MethylC-Seq; GSE56697), which was originally provided in mm9 coordinates and was converted to mm10 before comparison with ESC and aESC datasets.

Public WGBS datasets for ICM, WT ESCs, and androgenetic ESCs were used to compare gene-associated methylation differences across the four RNA-seq-defined gene classes. Gene-associated regions were defined using mouse gene coordinates, and CpG methylation values overlapping each gene region were aggregated. For each gene-associated region, methylation was calculated as the mean CpG methylation level across covered CpG sites. WT ESC methylation differences were calculated relative to ICM using ES-f3, and aESC methylation differences were calculated relative to ICM using the mean methylation level of aES2 and aES3. For mouse WGBS data, BP minus WT methylation differences were calculated for each gene-associated region in each of the five tissues using the mean of two BP and two WT biological replicates, and then summarized across tissues with valid methylation estimates. Dot-strip plots show individual gene-associated regions, with vertical solid lines indicating median ΔmCG and dashed lines indicating ΔmCG = 0.

### Element-level methylation difference and BP-hypermethylated DMR enrichment analysis

Element-level methylation was calculated for CDS, exon, LINE_L1, LINE_L2, LTR_ERV1, LTR_ERVK, LTR_ERVL, simple repeats, satellites, SINE_Alu, SINE_MIR, SINE_tRNA, TSS, 3′ UTR, 5′ UTR, and ICR regions using the corresponding BED annotations. For each sample and each genomic element class, CpG methylation was calculated as the total number of methylated reads divided by the total number of methylated plus unmethylated reads across all CpG sites overlapping that element class. For each organ, BP minus WT ΔmCG was calculated as the difference between the mean of two BP replicates and the mean of two WT replicates.

Differentially methylated regions were called independently for each organ using DSS from CpG-level methylated and total read counts. BP-hypermethylated DMRs were defined as DMRs with lower methylation in WT than BP, corresponding to diff.Methy ≤ −0.1. BP-hypermethylated DMRs from the five organs were merged to generate a non-redundant set of BP-hypermethylated regions. For each genomic element class, enrichment was calculated as the observed fraction of merged BP-hypermethylated DMR length overlapping the element divided by the expected fraction represented by that element among all selected genomic elements.

### RNA-seq library preparation

Total RNA was extracted from mouse embryos or organs using TRIzol reagent (Invitrogen) according to the manufacturer’s instructions. RNA libraries were prepared using the NEBNext Ultra RNA Library Prep Kit (NEB) and sequenced on an Illumina HiSeq X Ten platform to generate 150-bp paired-end reads.

### RNA-seq analysis

Transcriptome sequencing data were aligned to the mouse reference genome (GRCm39) using STAR. Gene expression levels for each sample were quantified using StringTie. Differential expression analysis between BP and control groups was performed for each tissue using DESeq2 [29–30]. Six biological replicates were analyzed for E12.5 embryos and four biological replicates were analyzed for term tissues. Differentially expressed genes (DEGs) were identified based on the following criteria: fold change ≥ 1.5 and adjusted *P*-value < 0.05. Genes with low expression were filtered prior to differential expression analysis. Specifically, genes were required to have FPKM >1 in at least three samples within either group to be considered expressed. Imprinted genes among the DEGs were annotated based on the list of mouse imprinted genes from the Geneimprint database. KEGG pathway enrichment analysis of DEGs was performed using clusterProfiler, with a significance threshold of adjusted *P*-value < 0.05. GO pathway enrichment analysis of DEGs was performed using clusterProfiler, with a significance threshold of adjusted *P*-value < 0.05. PCA dimensionality reduction was conducted using factoMineR, and the results were visualized with factoextra. Sample correlations were calculated using the psych package, and correlation heatmaps were generated with corrplot. Sequencing depth, mapping rate, and numbers of detected genes for each sample are summarized in S1 Table. All E12.5 whole-embryo RNA-seq samples, including WT, WT TC, and BP TC embryos, were prepared and sequenced in the same batch. For the nine-organ transcriptomic dataset from term samples, the original samples and additional biological replicates generated during revision were sequenced in two RNA-seq batches. Batch effects between the two RNA-seq batches were modeled using DESeq2 by incorporating batch as a covariate in the design formula. For exploratory analyses, including PCA, CV analysis, and correlation analysis, batch-adjusted expression values were generated before downstream visualization and comparison.

### E12.5 RNA-seq gene classification and expression perturbation analysis

For the E12.5 whole-embryo RNA-seq dataset, genes were first filtered based on expression abundance. Genes with FPKM > 1 in at least three samples within any of the three groups were retained for downstream analysis. Differential expression was analyzed using DESeq2 with group as the main variable. Pairwise comparisons were performed for WT TC versus WT, BP TC versus WT, and BP TC versus WT TC. Unless otherwise specified, genes were considered differentially expressed using an adjusted *P*-value < 0.1 and an absolute log2 fold-change > 0.2.

Expressed genes were classified into four categories according to their expression patterns across WT, WT TC, and BP TC embryos. Stable genes showed no significant group effect and small pairwise expression differences among the three groups. WT TC-shifted baseline genes were defined as genes for which WT TC and BP TC were both significantly shifted from WT in the same direction, while BP TC remained close to WT TC. BP-amplified genes were defined as genes for which WT TC and BP TC were both shifted from WT in the same direction, with BP TC showing a stronger deviation than WT TC. BP-specific genes were defined as genes significantly altered in BP TC relative to both WT and WT TC, while WT TC remained close to WT. Imprinted gene enrichment for each gene class was calculated as the observed fraction of imprinted genes divided by the expected fraction among all classified expressed genes, and statistical significance was assessed using a hypergeometric test.

For the expression deviation heatmap in Fig 2E, gene expression was transformed as log2(FPKM + 1), and each gene was centered by subtracting the mean value of the WT samples. The imprinted gene perturbation score was calculated from expressed genes present in the imprinted gene list by summarizing their deviation from the WT mean in each sample. The Euclidean distance to the WT centroid was calculated in PCA space using variance-stabilized expression values from all expressed genes; principal components explaining 80% of the cumulative variance were retained. Pathway perturbation scores were calculated from Reactome pathway activity scores inferred by GSVA, and each sample was summarized by its distance from the WT centroid across pathway scores.

### Gene expression variability analysis

The CV was calculated to quantify gene expression variability across biological replicates according to the formula: CV = *σ*/*μ*, where *σ* represents the standard deviation and *μ* represents the mean expression level of each gene. CV was calculated based on FPKM values for each gene across biological replicates. Within each group (WT, WT TC, and BP TC), genes were ranked in descending order of their CV and stratified into four quartile-based stability tiers: the top 25% were classified as highly variable, the 25%–50% quartile as moderately variable, the 50%–75% quartile as relatively stable, and the 75%–100% quartile as stable. An alluvial (Sankey-style) diagram was then generated using ggplot2 and ggalluvial to visualize, for each gene, the assigned stability tier across the three groups and the transitions between tiers.

### GSVA and module score analysis

After differential expression analysis across E12.5 groups, we performed GO enrichment analysis with clusterProfiler and selected three representative GO terms (GO:0002181, GO:0045721, and GO:0006958). We then applied GSVA to compute enrichment scores for these gene sets in each sample, thereby characterizing pathway-level activity and highlighting differences in the activation patterns of these biological processes across samples and between groups.

### Metabolomics

The extraction of the Liver metabolome is based on previously published literature [31]. Liver samples were ground using a tissue grinder (5,000 rpm, 10 s, 2 cycles, 5 s pause between cycles). The homogenized samples were incubated with shaking at 1,500 rpm for 30 min at 4 °C. After incubation, samples were centrifuged at 12,000 rpm for 10 min at 4 °C, and the supernatant was transferred to 1.5 mL centrifuge tubes. The samples were then dried using a centrifugal concentrator (Genevac miVac, Tegent Scientific, UK). The dried extracts were reconstituted in 100 µL of 1% acetonitrile, and the supernatant was collected for LC–MS analysis. Chromatographic separation was performed using an ACQUITY UPLC HSS T3 column (1.8 μm, 2.1 × 100 mm; Waters, Dublin, Ireland). An ultra-high-performance liquid chromatography system (Agilent 1290 II, Agilent Technologies, Germany) coupled with a high-resolution mass spectrometer (TripleTOF 5600 Plus, AB Sciex, Singapore) was employed. Analyses were conducted in both positive and negative electrospray ionization (ESI±) modes under the following conditions: curtain gas = 35, ion spray voltage = +5,500 V (positive mode)/−4,500 V (negative mode), temperature = 450 °C, ion source gas 1 = 50, ion source gas 2 = 50.

### Quantification and statistical analysis

Unless otherwise stated, statistical analyses were performed using Student *t* test. RNA-seq differential expression analysis was performed using DESeq2 with Benjamini-Hochberg correction for multiple testing. Enrichment analyses were conducted using clusterProfiler.

## Supporting information

S1 Fig Placental efficiency and transcriptomic stability analysis across WT, WT TC, and BP TC groups. **(A)** Placental weight normalized to the mean fetal weight in WT, 7KO, 10KO, 19KO, and 20KO mice. Each dot represents one placenta. **(B)** Quantification of Euclidean distances in PCA space among E12.5 embryos from WT, WT TC, and BP TC groups, based on the PCA shown in D. Each dot represents an individual embryo. Boxes indicate the median and interquartile range. **(C)** Pairwise correlation analysis of RNA-seq transcriptomes from E12.5 embryos. Heatmap showing Pearson correlation coefficients between RNA-seq samples from WT, WT TC, and BP TC groups at E12.5. Samples are ordered by genotype as indicated. Color scale represents correlation strength. **(D)** Gene set enrichment analysis (GSVA)-based module scores for selected Gene Ontology (GO) biological processes in individual E12.5 embryos from WT, WT TC, and BP TC groups. Three representative GO terms associated with essential cellular and metabolic activities—cytoplasmic translation, complement activation, and glucose metabolic process—are shown. Each dot represents the GSVA module score of an individual embryo for the indicated pathway. The dotted line denotes a module score of zero. **(E)** Probability density distributions of the CV for all expressed genes across six biological replicates in WT, WT TC, and BP TC E12.5 embryos. CV values were log-transformed as log10(CV + 1). Dashed vertical lines indicate the 25%, 50% (median), and 75% percentiles of the CV distribution for each group. **(F)** Sankey diagram showing gene stability state transitions across groups based on CV quartiles. Genes were classified into four stability categories according to their coefficient of variation (CV) quartiles: Very stable, Stable, Variable, and Highly variable. The Sankey diagram illustrates the correspondence and transitions of gene stability states across the WT, WT TC, and BP TC groups. Horizontal bars represent the number of genes in each stability category within a given group, while the connecting flows indicate how individual genes change their stability classification between groups. The width of each flow is proportional to the number of genes, and the color gradient from red to blue reflects increasing variability (from low to high CV). **(G)** Distribution of stability states of imprinted genes across groups. Pie charts show the proportion of imprinted genes classified into four stability categories (Very stable, Stable, Variable, and Highly variable) based on coefficient of variation (CV) quartiles in the WT, WT TC, and BP TC groups. Colors correspond to different stability states, illustrating differences in the stability composition of imprinted genes among the three groups. Data are mean ± SEM; *p < 0.05, **p < 0.01, ***p < 0.001, ****p < 0.0001. The data underlying this Figure can be found in S6 Data.
(TIF)

S2 Fig Expression variability of representative high-variability genes is independent of sequencing depth. Four representative genes (Egr1, Tmsb10b, Xlr3a, and Xlr3b), ranked among the top four by coefficient of variation (CV) in the BP group, are shown. Upper panels display gene expression levels (FPKM) across WT, WT TC, and BP TC samples. Lower panels show gene expression levels plotted against sequencing depth, measured as total read counts per sample. No clear linear relationship between expression and total reads is observed, indicating that the high expression variability of these genes is not driven by sequencing depth but reflects intrinsic biological variation among samples. The data underlying this Figure can be found in S6 Data.
(TIF)

S3 Fig Transcriptomic PCA of multiple organs in WT and BP mice and statistical analysis of CV values. **(A)** Principal component analysis (PCA) of RNA-seq transcriptomes from multiple organs of WT and BP mice collected at term. PCA was performed separately for each tissue, including kidney, diaphragm, heart, intestine, lung, muscle, spleen, and stomach. Each dot represents an individual biological replicate (*n* = 4 per group). Samples are colored and labeled according to genotype (WT or BP). The percentage of variance explained by Dim1 and Dim2 is indicated on each axis. **(B)** Probability density distributions of log₁₀(CV_BP/CV_WT) for all expressed genes across multiple organs, including diaphragm, kidney, heart, intestine, liver, muscle, spleen, and stomach. Gene-wise coefficients of variation (CV) were calculated across four biological replicates for each genotype. Positive values indicate higher expression variability in BP relative to WT, whereas negative values indicate lower variability. Numbers indicate the total number of genes included for each tissue. The data underlying this Figure can be found in S6 Data.
(TIF)

S4 Fig Association between expression variability and differential gene expression across multiple organs. **(A)** Scatter plot showing the relationship between gene expression fold change (log2 fold change) and expression variability change (log10(CV_BP/CV_WT)) in multiple organs, including diaphragm, kidney, heart, intestine, liver, muscle, spleen, and stomach. Each dot represents an individual differentially expressed gene. Imprinted genes with higher CV in BP are highlighted and labeled. The dashed line represents the threshold for differential expression (log2 fold change ≥ 1.5), and the full horizontal line indicates the threshold for higher CV in BP relative to WT (log10(CV_BP/CV_WT) > 0). **(B)** KEGG pathway enrichment analysis of non-imprinted genes positively or negatively correlated with imprinted gene expression in multiple organs, including diaphragm, kidney, heart, intestine, liver, muscle, spleen, and stomach. Non-imprinted genes were grouped based on their correlation with imprinted genes, and subjected to KEGG enrichment analysis. Positively correlated genes are shown in red and negatively correlated genes in blue. The x-axis indicates −log(*P*-value). The data underlying this Figure can be found in S6 Data.
(TIF)

S5 Fig Expression levels of representative imprinting genes across technical replicates in multiple organs. Several imprinting genes that are robustly expressed across all nine organs were selected for analysis. Gene expression levels are shown for BP and WT samples across technical replicates for each tissue. Consistent expression patterns across replicates indicate that the observed variability is not driven by sequencing-related technical variation, supporting a biological basis for the differential expression variability observed across organs. The data underlying this Figure can be found in S6 Data.
(TIF)

S6 Fig Global DNA methylation levels across mouse tissues in WT and BP mice. **(A)** Global mCG levels across five mouse tissues in WT and BP samples. Each dot represents one WGBS sample. The data underlying this Figure can be found in S6 Data.
(TIF)

S7 Fig Metabolic pathway disruption and skeletal morphology changes in BP mice. **(A)** Metabolite set enrichment analysis of differential metabolites. Enriched metabolic pathways are shown based on metabolite set analysis, with the x-axis indicating −log₁₀(*P*-value). Pathways related to energy metabolism, mitochondrial function, and lipid metabolism are among the significantly enriched metabolite sets. **(B)** Quantification of selected metabolites showing significant differences between WT and BP samples. Metabolite levels are shown relative to WT. Data are presented as mean ± SD, with individual data points representing biological replicates. **(C)** Heatmap of expression levels of key tricarboxylic acid (TCA) cycle-related enzyme genes. Gene expression was derived from RNA-seq data and visualized for BP and WT samples. Selected genes encode enzymes involved in central carbon metabolism and were chosen based on the transcriptomic analyses shown in Fig 5. Colors indicate scaled expression levels across samples, with red representing higher expression and blue representing lower expression. **(D)** Representative magnetic resonance imaging (MRI) of skeletons from 6-week-old WT and BP mice. Whole-body skeletal images reveal differences in axial skeletal morphology between genotypes, as indicated by arrows. Images shown are representative of each group. Scale bar, 1 cm. Data are mean ± SEM; *p < 0.05, **p < 0.01, ***p < 0.001. The data underlying this Figure can be found in S6 Data.
(TIF)

S1 Table RNA-seq sequencing summary. Sequencing depth, mapping rate, and numbers of detected genes for each RNA-seq sample.
(XLSX)

S2 Table WGBS sequencing summary. Sequencing depth, mapping efficiency, and CpG coverage metrics for each WGBS sample.
(XLSX)

S1 Data Underlying data for Fig 1. (XLSX)

S2 Data Underlying data for Fig 2. (XLSX)

S3 Data Underlying data for Fig 3. (XLSX)

S4 Data Underlying data for Fig 4. (XLSX)

S5 Data Underlying data for Fig 5. (XLSX)

S6 Data Underlying data for Supplementary Figures. (XLSX)

S1 Code Custom code used for data processing and figure generation. (MD)

## Abbreviations

adESC: androgenetic diploid embryonic stem cells
ahESCs: androgenetic haploid embryonic stem cells
BP: bipaternal
CV: coefficient of variation
DEGs: differentially expressed genes
ICSI: intracytoplasmic sperm injection
PCA: principal component analysis
QMR: quantitative magnetic resonance
TC: tetraploid complementation
TCA: tricarboxylic acid
WT: wild-type
