# Supplementary figures and images for: Engineered bipaternal mice reveal the consequences of life without a maternal genomic contribution
Source: PLoS Biol. 2026 Jun 25;24(6):e3003871. doi: 10.1371/journal.pbio.3003871 (PMC13298975; doi:10.1371/journal.pbio.3003871)

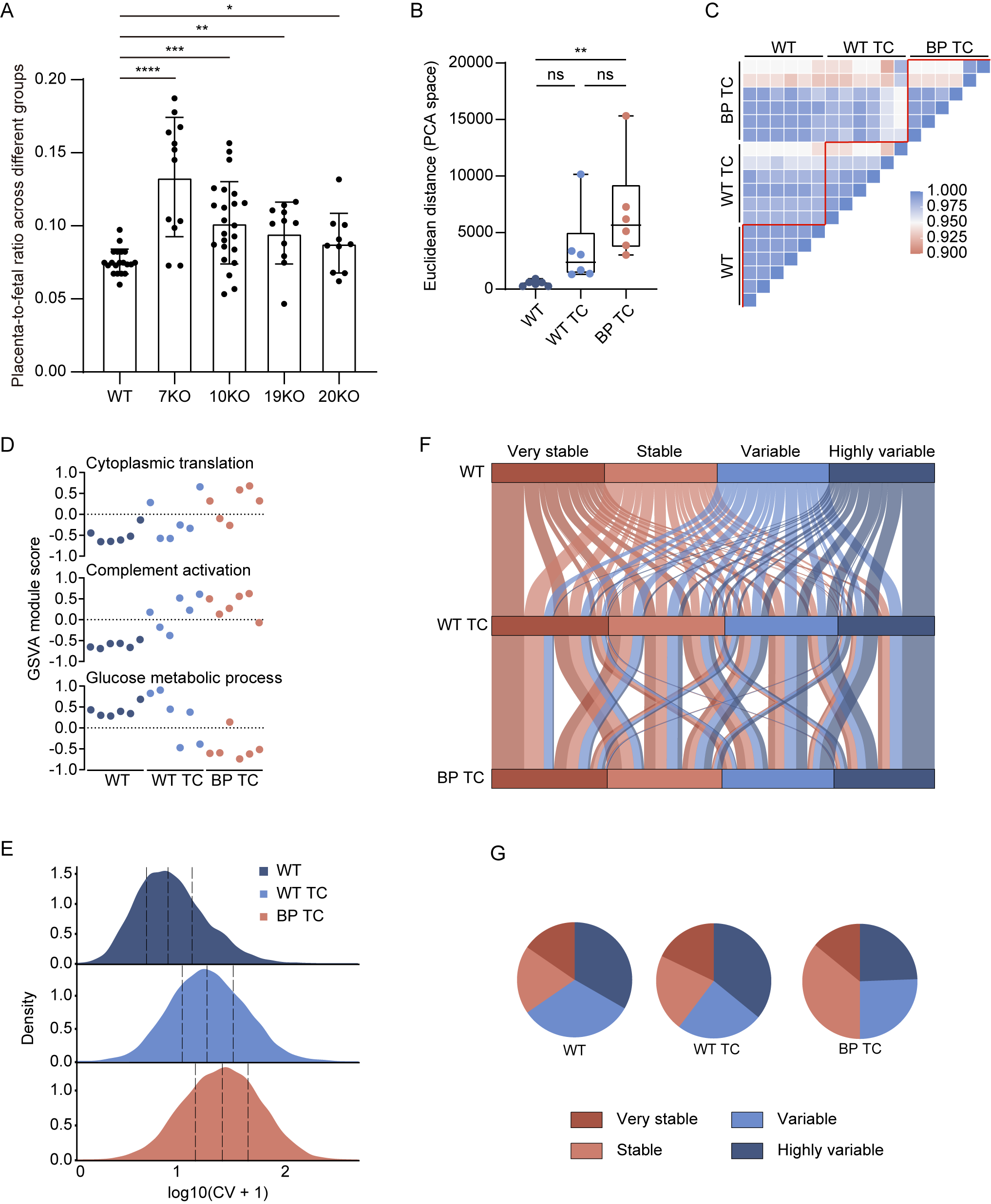

Supplement: S1 Fig — (A) Placental weight normalized to the mean fetal weight in WT, 7KO, 10KO, 19KO, and 20KO mice. Each dot represents one placenta. (B) Quantification of Euclidean distances in PCA space among E12.5 embryos from WT, WT TC, and BP TC groups, based on the PCA shown in D. Each dot represents an individual embryo. Boxes indicate the median and interquartile range. (C) Pairwise correlation analysis of RNA-seq transcriptomes from E12.5 embryos. Heatmap showing Pearson correlation coefficients between RNA-seq samples from WT, WT TC, and BP TC groups at E12.5. Samples are ordered by genotype as indicated. Color scale represents correlation strength. (D) Gene set enrichment analysis (GSVA)-based module scores for selected Gene Ontology (GO) biological processes in individual E12.5 embryos from WT, WT TC, and BP TC groups. Three representative GO terms associated with essential cellular and metabolic activities—cytoplasmic translation, complement activation, and glucose metabolic process—are shown. Each dot represents the GSVA module score of an individual embryo for the indicated pathway. The dotted line denotes a module score of zero. (E) Probability density distributions of the CV for all expressed genes across six biological replicates in WT, WT TC, and BP TC E12.5 embryos. CV values were log-transformed as log10(CV + 1). Dashed vertical lines indicate the 25%, 50% (median), and 75% percentiles of the CV distribution for each group. (F) Sankey diagram showing gene stability state transitions across groups based on CV quartiles. Genes were classified into four stability categories according to their coefficient of variation (CV) quartiles: Very stable, Stable, Variable, and Highly variable. The Sankey diagram illustrates the correspondence and transitions of gene stability states across the WT, WT TC, and BP TC groups. Horizontal bars represent the number of genes in each stability category within a given group, while the connecting flows indicate how individual genes change [file pbio.3003871.s001.tif]

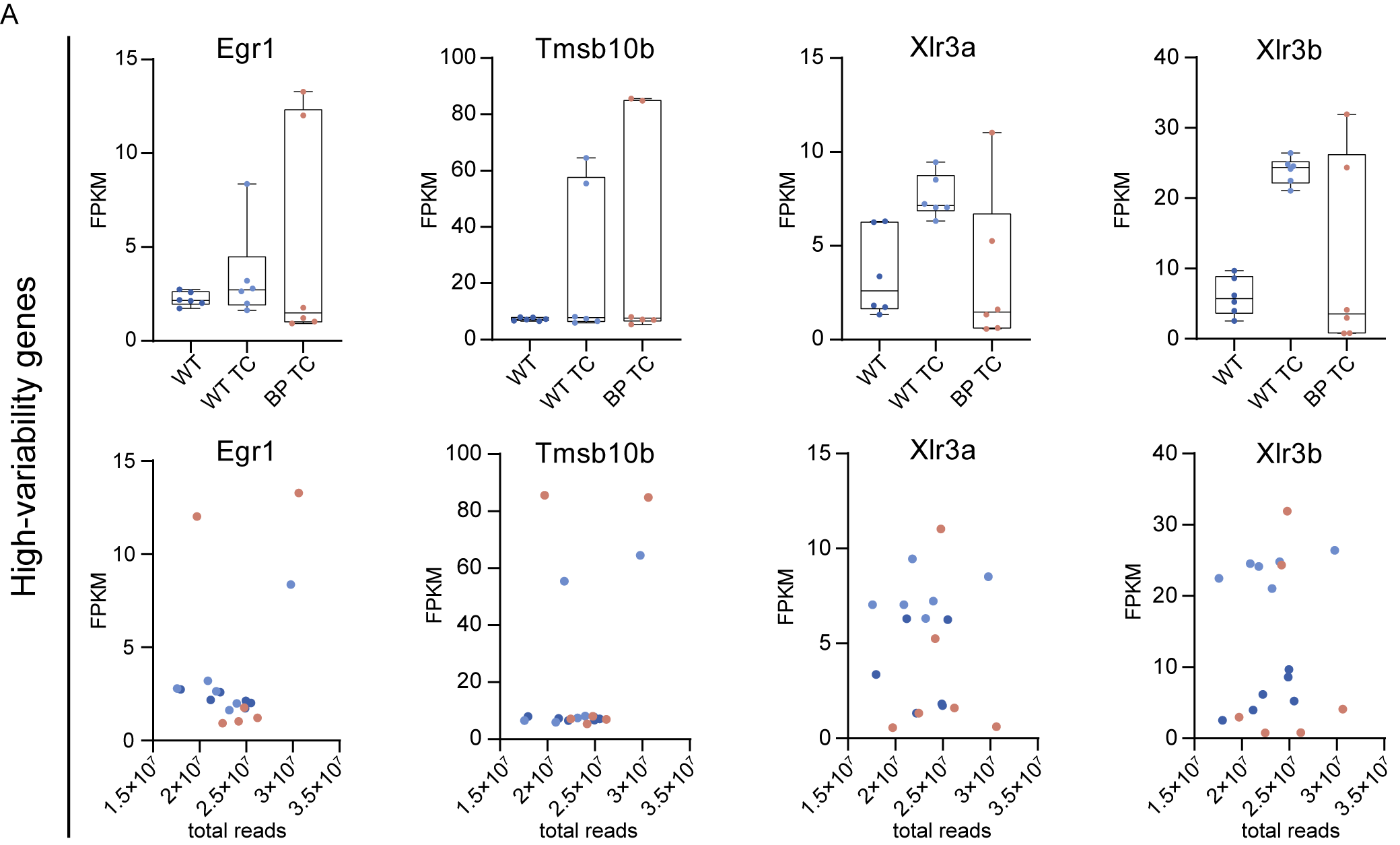

Supplement: S2 Fig — Four representative genes (Egr1, Tmsb10b, Xlr3a, and Xlr3b), ranked among the top four by coefficient of variation (CV) in the BP group, are shown. Upper panels display gene expression levels (FPKM) across WT, WT TC, and BP TC samples. Lower panels show gene expression levels plotted against sequencing depth, measured as total read counts per sample. No clear linear relationship between expression and total reads is observed, indicating that the high expression variability of these genes is not driven by sequencing depth but reflects intrinsic biological variation among samples. The data underlying this Figure can be found in S6 Data. (TIF) [file pbio.3003871.s002.tif]

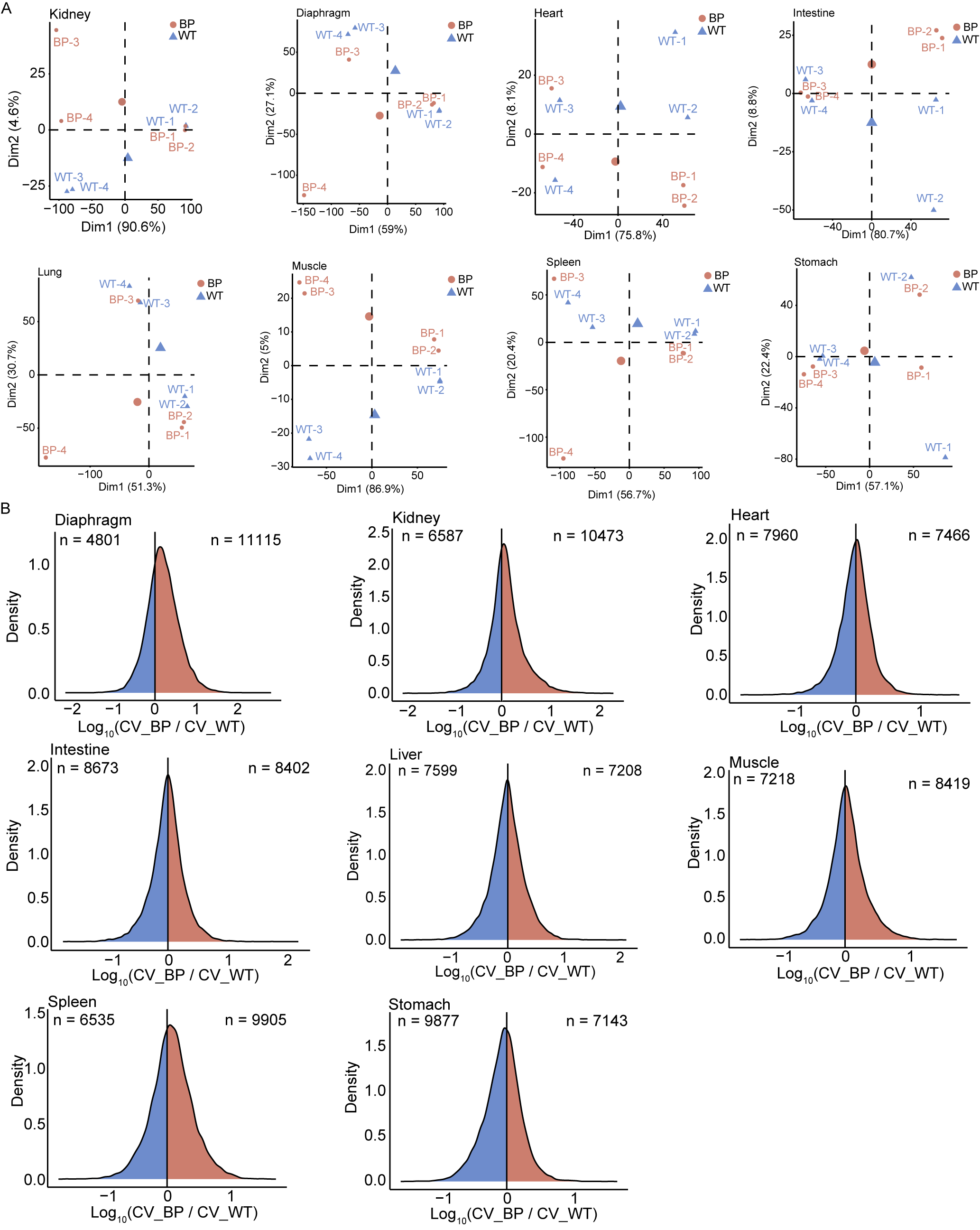

Supplement: S3 Fig — (A) Principal component analysis (PCA) of RNA-seq transcriptomes from multiple organs of WT and BP mice collected at term. PCA was performed separately for each tissue, including kidney, diaphragm, heart, intestine, lung, muscle, spleen, and stomach. Each dot represents an individual biological replicate (n = 4 per group). Samples are colored and labeled according to genotype (WT or BP). The percentage of variance explained by Dim1 and Dim2 is indicated on each axis. (B) Probability density distributions of log₁₀(CV_BP/CV_WT) for all expressed genes across multiple organs, including diaphragm, kidney, heart, intestine, liver, muscle, spleen, and stomach. Gene-wise coefficients of variation (CV) were calculated across four biological replicates for each genotype. Positive values indicate higher expression variability in BP relative to WT, whereas negative values indicate lower variability. Numbers indicate the total number of genes included for each tissue. The data underlying this Figure can be found in S6 Data. (TIF) [file pbio.3003871.s003.tif]

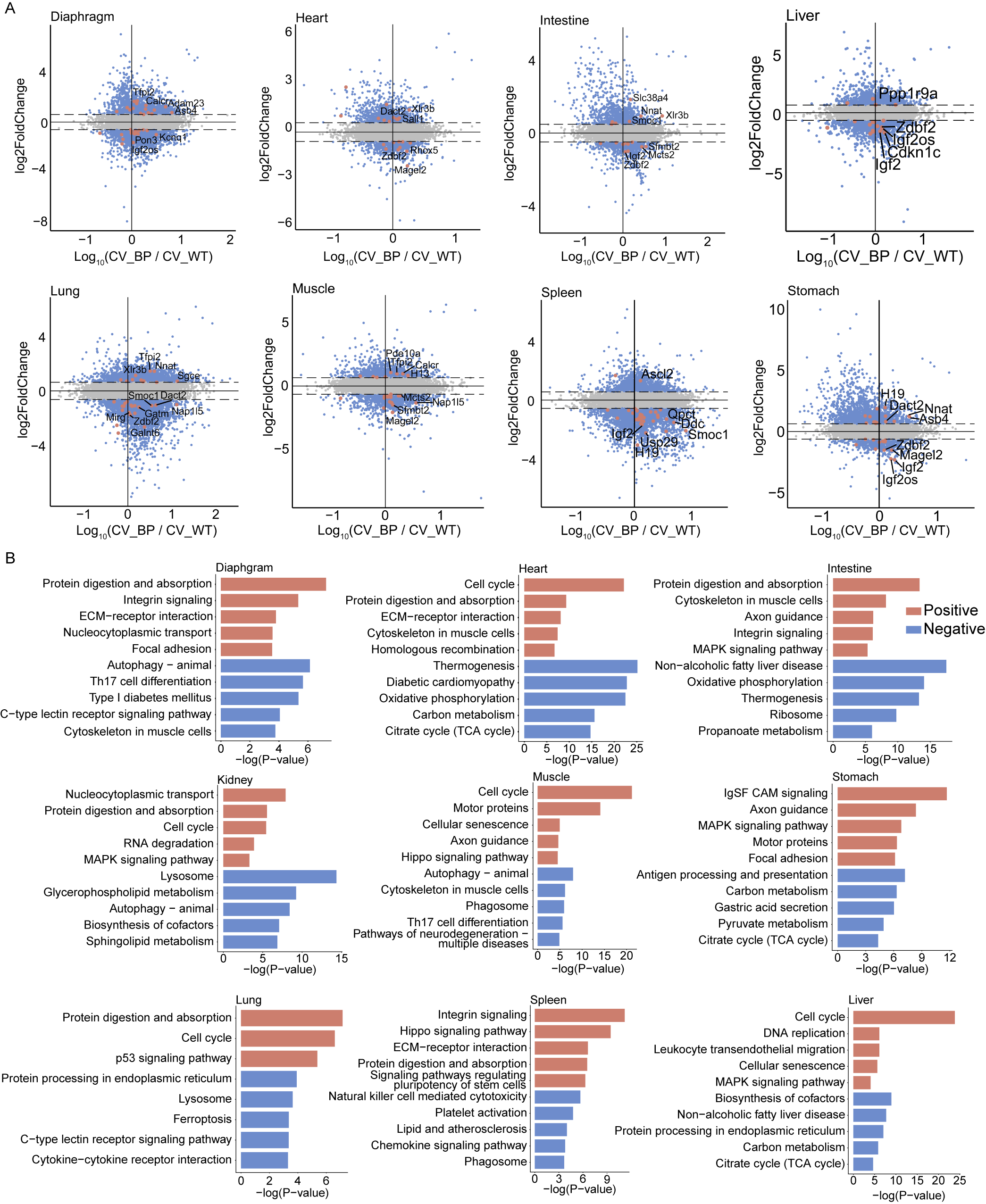

Supplement: S4 Fig — (A) Scatter plot showing the relationship between gene expression fold change (log2 fold change) and expression variability change (log10(CV_BP/CV_WT)) in multiple organs, including diaphragm, kidney, heart, intestine, liver, muscle, spleen, and stomach. Each dot represents an individual differentially expressed gene. Imprinted genes with higher CV in BP are highlighted and labeled. The dashed line represents the threshold for differential expression (log2 fold change ≥ 1.5), and the full horizontal line indicates the threshold for higher CV in BP relative to WT (log10(CV_BP/CV_WT) > 0). (B) KEGG pathway enrichment analysis of non-imprinted genes positively or negatively correlated with imprinted gene expression in multiple organs, including diaphragm, kidney, heart, intestine, liver, muscle, spleen, and stomach. Non-imprinted genes were grouped based on their correlation with imprinted genes, and subjected to KEGG enrichment analysis. Positively correlated genes are shown in red and negatively correlated genes in blue. The x-axis indicates −log(P-value). The data underlying this Figure can be found in S6 Data. (TIF) [file pbio.3003871.s004.tif]

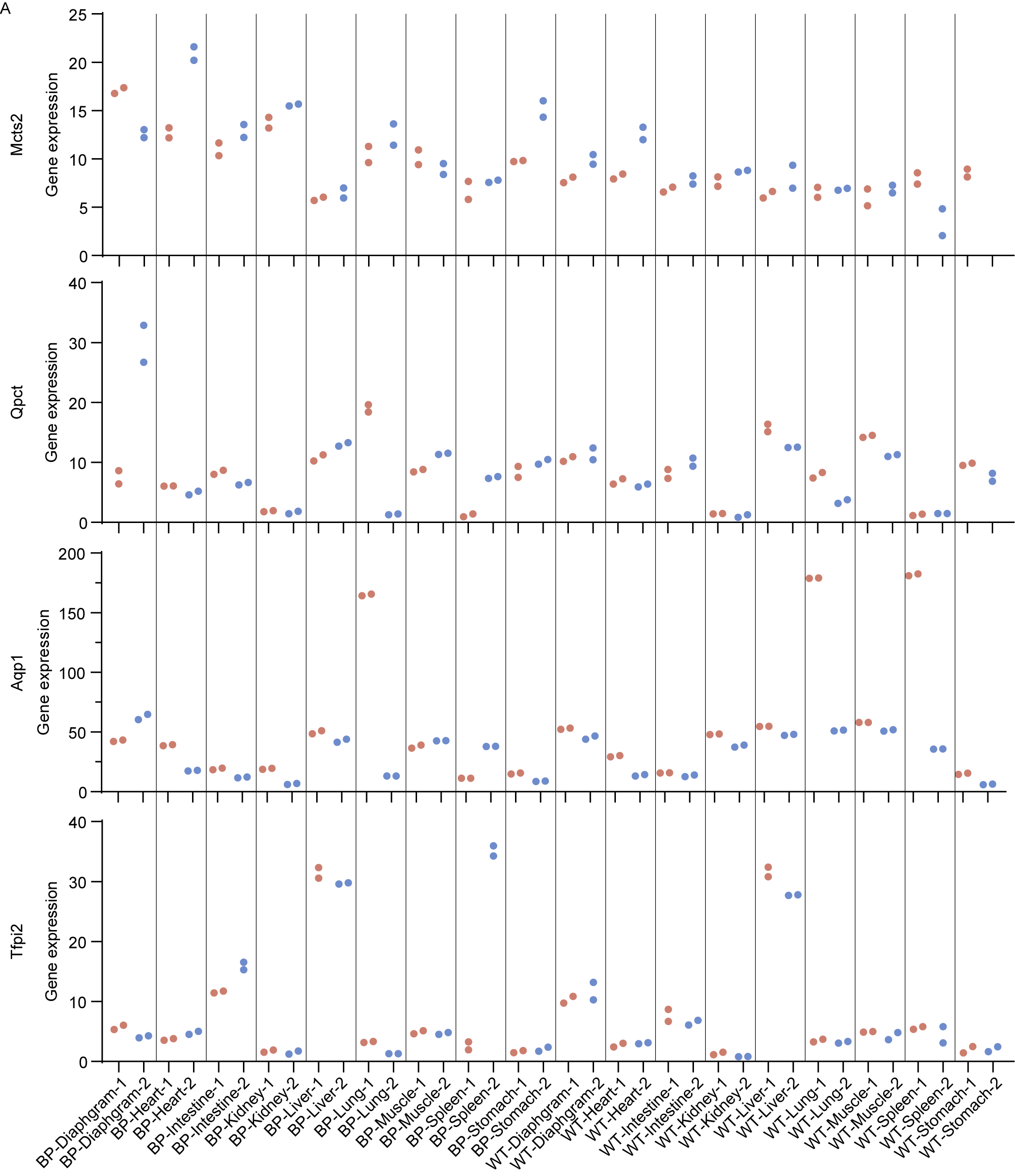

Supplement: S5 Fig — Several imprinting genes that are robustly expressed across all nine organs were selected for analysis. Gene expression levels are shown for BP and WT samples across technical replicates for each tissue. Consistent expression patterns across replicates indicate that the observed variability is not driven by sequencing-related technical variation, supporting a biological basis for the differential expression variability observed across organs. The data underlying this Figure can be found in S6 Data. (TIF) [file pbio.3003871.s005.tif]

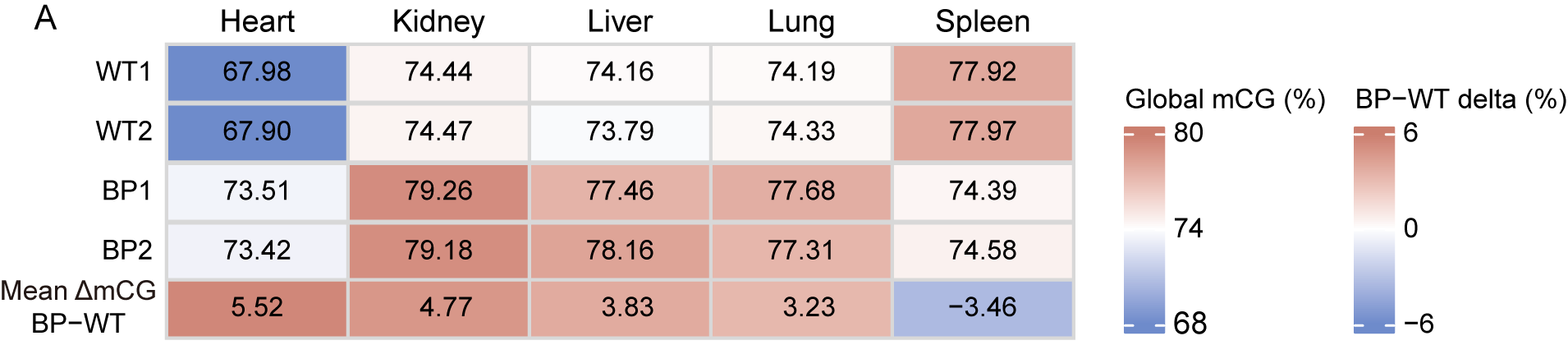

Supplement: S6 Fig — (A) Global mCG levels across five mouse tissues in WT and BP samples. Each dot represents one WGBS sample. The data underlying this Figure can be found in S6 Data. (TIF) [file pbio.3003871.s006.tif]

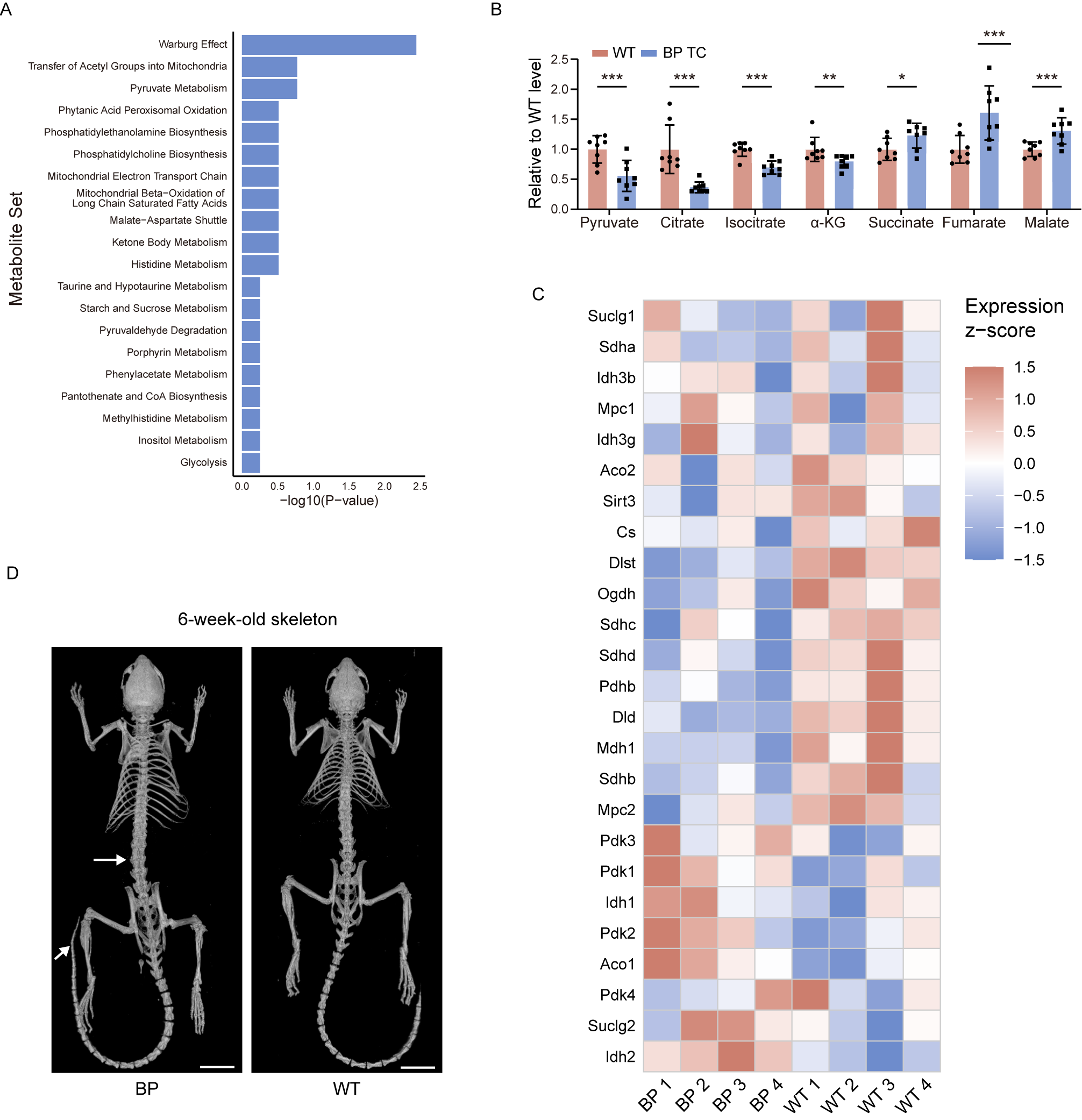

Supplement: S7 Fig — (A) Metabolite set enrichment analysis of differential metabolites. Enriched metabolic pathways are shown based on metabolite set analysis, with the x-axis indicating −log₁₀(P-value). Pathways related to energy metabolism, mitochondrial function, and lipid metabolism are among the significantly enriched metabolite sets. (B) Quantification of selected metabolites showing significant differences between WT and BP samples. Metabolite levels are shown relative to WT. Data are presented as mean ± SD, with individual data points representing biological replicates. (C) Heatmap of expression levels of key tricarboxylic acid (TCA) cycle-related enzyme genes. Gene expression was derived from RNA-seq data and visualized for BP and WT samples. Selected genes encode enzymes involved in central carbon metabolism and were chosen based on the transcriptomic analyses shown in Fig 5. Colors indicate scaled expression levels across samples, with red representing higher expression and blue representing lower expression. (D) Representative magnetic resonance imaging (MRI) of skeletons from 6-week-old WT and BP mice. Whole-body skeletal images reveal differences in axial skeletal morphology between genotypes, as indicated by arrows. Images shown are representative of each group. Scale bar, 1 cm. Data are mean ± SEM; *p < 0.05, **p < 0.01, ***p < 0.001. The data underlying this Figure can be found in S6 Data. (TIF) [file pbio.3003871.s007.tif]
