# Supplementary material for: Engineered bipaternal mice reveal the consequences of life without a maternal genomic contribution
Source: PLoS Biol. 2026 Jun 25;24(6):e3003871. doi: 10.1371/journal.pbio.3003871 (PMC13298975; doi:10.1371/journal.pbio.3003871)
Supplement: Attachment — Submitted filename: Point-to-point response.pdf [file pbio.3003871.s017.pdf]

POINT-TO-POINT RESPONSE

We sincerely thank the reviewers for their careful reading of our manuscript and for their thoughtful and constructive critiques. These comments led us to re-evaluate both the evidential basis and the interpretive scope of the study, and resulted in major improvements throughout the revised manuscript. In response, we carried out extensive additional experiments and analyses, including expanded biological replication, improved control frameworks, and new placental and embryonic characterization, while also substantially clarifying the methodological presentation and reorganizing the Results. Just as importantly, we revised the framing of the study so that the conclusions more closely match what the data can firmly support. As a result, the revised manuscript is not simply a narrower version of the original submission, but a substantially strengthened and more rigorously supported study. We respond to each comment in detail below.

REVIEWERS' COMMENTS:

Reviewer #1:

In this manuscript, the authors extended their similar earlier studies. Interestingly, they now derived bipaternal neonatal mice at a much higher frequency than before. These animals were analysed at day-1after birth for their gene expression and DNA methylation profiles. The interesting approach taken was the use of early tetraploid embryos, which were fused with bipaternal embryos, which were obtained by injection of sperm and a nucleus from previously-derived haploid ESCs with 20 ICR deletions. Interestingly, since the extra-embryonic development was now supported by the tetraploid cells predominantly, the authors found as could be expected that the development of the embryonic proceeded much better, often to birth. As such, this finding is entirely in line with the major importance of imprinted gene expression in the extra-embryonic lineages. Whether, besides the technology improvement and the observed enhanced development, any other conclusions can be drawn from this study is unclear to this reviewer. In particular, given the many limitations of the

genetic approach taken, and the diverse manipulations required, this study does not allow one to conclude that the maternal and paternal genomes provide non-redundant contributions beyond imprinting. This substantially reduces the interest of this study.

Response:

We thank the reviewer for the careful reading of our manuscript and for the thoughtful evaluation of this study. We are grateful that the reviewer recognized the use of tetraploid complementation as an interesting experimental approach, and noted that the improved developmental outcome observed in this system is consistent with the well-established importance of imprinted gene regulation in extraembryonic lineages.

At the same time, the review also raised several questions that drew our attention to aspects of the study that merited closer examination. Revisiting these points during revision led us to reconsider several observations from the original submission and to examine them in greater detail. Following these comments, we carried out additional experiments and expanded a number of analyses in order to investigate bipaternal development more comprehensively.

These additional data allowed us to reassess several observations reported in the original manuscript and to place them within a clearer developmental context. On this basis, the manuscript has also been revised to define more explicitly the scope of the study and the framework within which these observations can be interpreted. The revised version therefore focuses more directly on the developmental and physiological characteristics observed in bipaternal mice, while keeping the interpretation of these findings within the limits of the present experimental system.

We respond to the reviewer's specific comments in detail below.

Main points :

-A major flaw in the reasoning is that 'imprinting correction' is not achieved by the deletion of the imprinting control regions (ICRs). Given the diverse regulatory functions of the ICRs, both as repressors and activators of gene expression in cis, the

deletion ICRs is expected to often induce aberrant expression of the close-by imprinted genes. This is indeed seen, with many imprinted genes showing highly abnormal experssion levels in the BP mice. For instance, deletion of the ICR of the Igf2-H19 domain in the ESC-derived genome removes its insulator function, similarly as on the sperm-derived genome in which this function is inactivated by DNA methylation. At the ICR of the Dlk1-Dio3 domain, similarly, the deletion akes away also its essential enhancer function, which is repressed also on the sperm-derived genome (by DNA methylation).

Response:

We thank the reviewer for this thoughtful discussion of the regulatory complexity of imprinting control regions (ICRs). As noted by the reviewer, these elements can serve multiple cis-regulatory roles, including insulator and enhancer activities, and their removal can therefore influence the expression of nearby imprinted genes in ways that differ from the epigenetic configurations established during normal gametogenesis.

These considerations also underscore an important feature of the experimental system used in the present study. The bipaternal configuration generated through targeted modifications at multiple parent-of-origin regulatory regions represents an experimentally engineered developmental setting rather than a direct reconstruction of the physiological imprinting program. Consequently, transcriptional states observed in this model likely reflect both the regulatory consequences of these modifications and the broader developmental context of bipaternal genomic composition.

Within this setting, the system provides a tractable context in which development proceeds in the absence of maternal genomic contribution. This configuration offers an opportunity to examine how mammalian developmental and physiological processes respond under such asymmetric genomic conditions.

From this perspective, the model serves as an experimental entry point for exploring a broader biological question—why mammalian, and more generally vertebrate, development so consistently depends on contributions from both parental genomes. Although the experimental configuration is necessarily simplified relative to natural

developmental processes, it allows certain aspects of this question to be examined in a controlled experimental framework.

-Imprinted genes are highly interconnected in gene expression networks (as described by Laurent Journot and his co-workers and others in the field), and this may well explain why so many of the imprinted genes showed aberrant expression in the BP animals.

Response:

We thank the reviewer for raising this point regarding the interconnected nature of imprinted genes. During revision, we examined the literature on regulatory relationships among imprinted loci more carefully, including the work from Laurent Journot and colleagues describing coordinated transcriptional networks of imprinted genes (Varrault et al., 2006; Al Adhami et al., 2015). We then re-evaluated the expression of several loci highlighted in these studies within our own datasets. In particular, we revisited the expression of Plagl1/Zac1, which has been discussed as a key node within imprinted gene networks. Re-examination of the datasets from bipaternal mice generated in our previous work indicated that the expression of this locus remains largely within the normal range. This observation suggested that the transcriptional alterations observed in bipaternal animals may not be readily captured by a simple disruption of a coordinated imprinted gene regulatory program.

To examine this issue more carefully, we extended the transcriptomic analysis in the revised study. In the original manuscript, bulk RNA-seq had been performed across eight tissues, each with two biological replicates. During revision, the number of biological replicates was increased to four per tissue, allowing a more reliable assessment of transcriptional patterns across organs and among individuals. Analysis of the expanded datasets showed that differential expression of both imprinted and non-imprinted genes remains detectable across tissues. At the same time, increasing the number of biological replicates revealed a feature that had been less apparent in the original analysis: despite identical genetic modifications, transcriptional profiles of

bipaternal animals show substantial divergence among individuals across multiple tissues. In other words, while expression differences can be detected at the level of gene lists, the overall transcriptomic profiles show a reduced degree of convergence among individuals.

In light of these observations, the revised manuscript presents the transcriptional alterations more cautiously as characteristics of the organ transcriptomes in bipaternal mice, rather than attributing them primarily to a specific imprinted gene network mechanism. At the same time, regulatory relationships among imprinted genes may still contribute to these patterns.

The expanded transcriptomic analyses across organs are presented below.

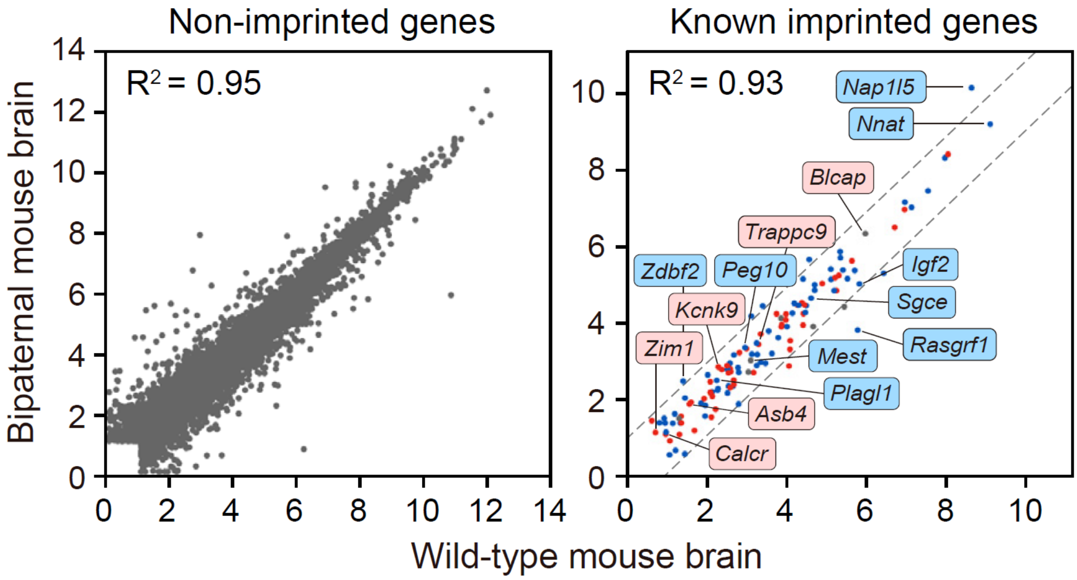

Panel 1. Evidence for largely restored Plagl1/Zac1 expression in bi-paternal mice. Previously reported brain expression data from the 20KO bi-paternal model showed that **Plagl1/Zac1**, a key node in imprinted gene networks, was largely restored toward the wild-type range. This observation motivated a broader re-evaluation of whether transcriptional abnormalities in BP mice could be explained by a simple disruption of an imprinted gene regulatory program.

Reproduced from Li et al., Cell Stem Cell, 2025

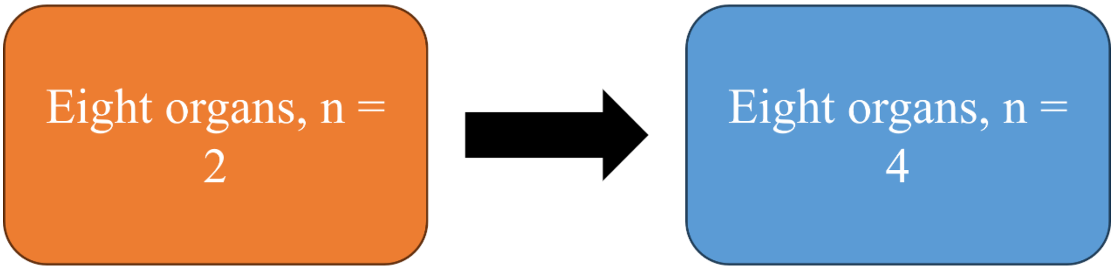

Panel 2. Expansion of organ transcriptomic profiling in the revised study.

In the original analysis, bulk RNA-seq was performed across eight tissues with two biological replicates per tissue. In the revised study, the number of biological replicates was increased to four per tissue, enabling a more reliable assessment of transcriptional patterns across organs and among individuals.

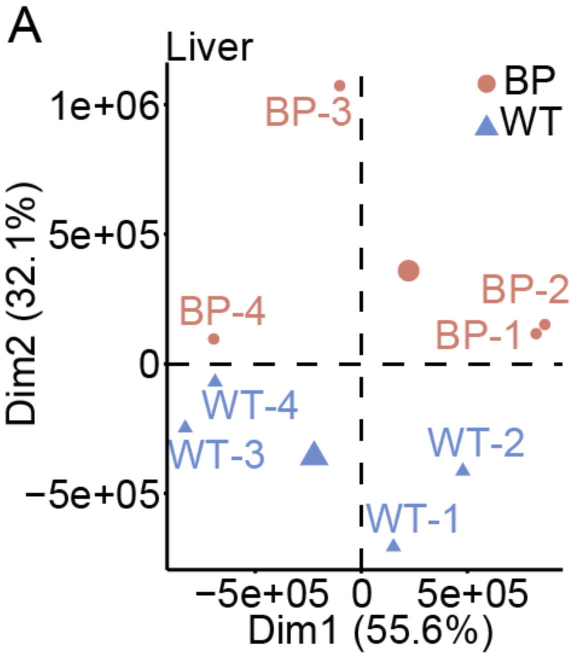

Panel 3. Representative PCA showing broader transcriptomic spread in a BP organ. Principal component analysis of a representative organ shows that BP samples occupy a broader transcriptional space than WT samples, consistent with reduced convergence among individual organ transcriptomes.

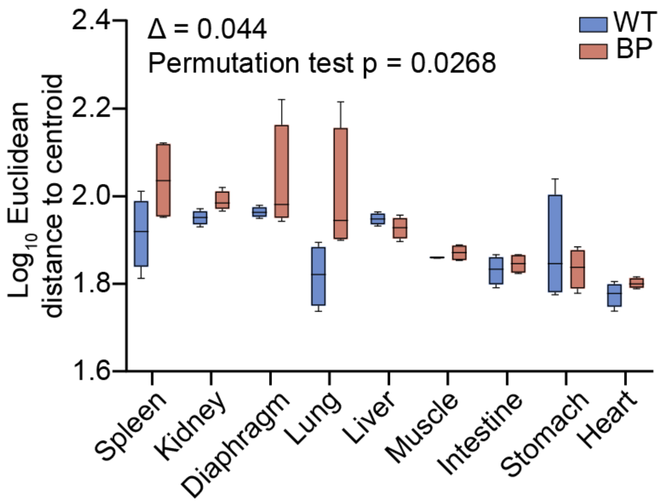

Panel 4. Broader dispersion of BP organ transcriptomes across multiple tissues. Quantification of Euclidean distance to the group centroid in PCA space shows that BP samples occupy a wider transcriptional space than WT controls across organs, indicating reduced convergence among organ transcriptomes.

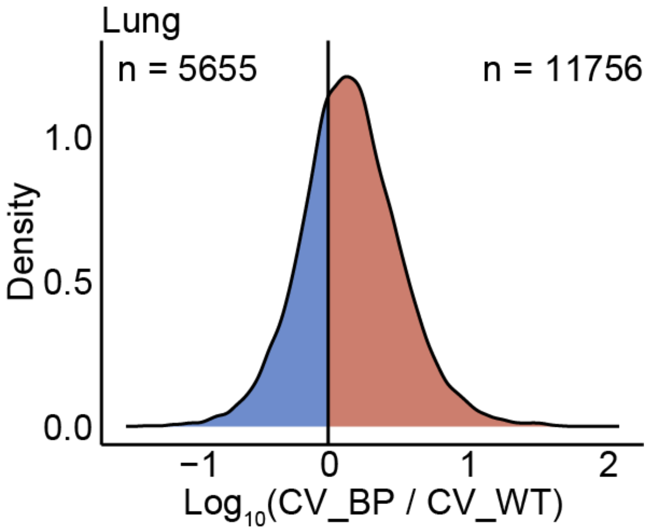

Panel 5. Representative distribution of gene-expression variability in BP tissue. A representative organ shows a rightward shift in gene-wise CV ratios in BP relative to WT, indicating broader expression ranges across a substantial fraction of the transcriptome.

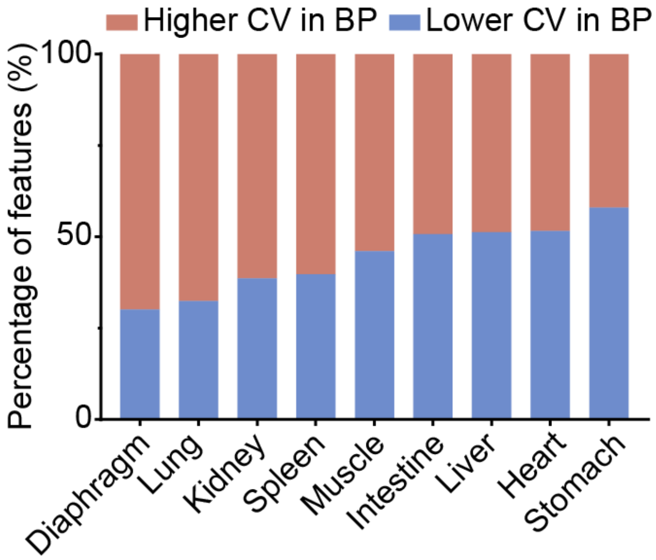

Panel 6. Broader gene-expression variability across multiple organs in BP mice.

Across organs, a substantial fraction of expressed genes shows higher CV in BP relative to WT, indicating that broader expression ranges extend across a large portion of the transcriptome rather than being restricted to a small set of loci.

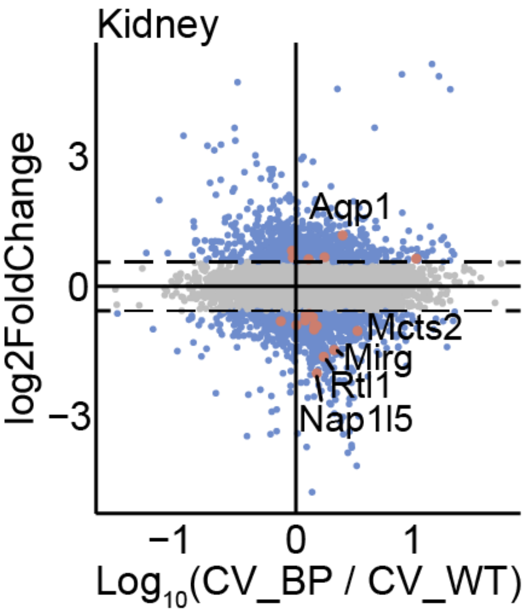

Panel 7. Imprinted loci remain involved within a broader variable transcriptional landscape.

Representative analysis shows that imprinted genes can still display altered expression and increased variability in BP mice, but these changes occur within a broader organ

transcriptomic context rather than as a uniform signature of a single imprinted-gene network mechanism.

In revisiting this aspect of the study, we first returned to the literature on imprinted gene regulatory networks and re-examined a key network node, Plagl1/Zac1, using previously published data from our 20KO bi-paternal model (Panel 1). We then expanded the organ transcriptomic analysis in the revised study from eight tissues with two biological replicates per tissue to eight tissues with four biological replicates per tissue (Panel 2). Representative PCA analyses showed broader transcriptomic spread in BP organs (Panel 3), and cross-organ quantification further showed that BP samples occupied a wider transcriptional space than WT controls (Panel 4). Gene-wise analyses likewise revealed broader expression variability in BP tissues, both in representative organs (Panel 5) and across organs overall (Panel 6). Finally, representative organ-level analysis showed that imprinted genes remained involved, but their altered expression occurred within a broader variable transcriptomic landscape rather than as a uniform signature of a single imprinted-gene network mechanism (Panel 7). These expanded organ transcriptomic analyses are presented below and are described in the revised Results section "Organ-specific transcriptional organization across multiple tissues in BP mice," particularly in the analyses corresponding to Fig. 3B–E and Fig. S5.

-Imprinted gene expresion evolved mostly relative to the placenta and the brain. About half the imprinted genes are imprinted and important specifically in the brain. For obvious reasons the authors did not explore gene expresion in the placenta, but why did they not continue brain tissues, or total brain ? This is unfortunate.

Response:

We thank the reviewer for raising this important point regarding the choice of tissues for evaluating imprinting-related effects. We agree that both the placenta and the brain represent particularly informative contexts for imprinting biology, and the reviewer's comment led us to reconsider more carefully what material would be most appropriate

for a more focused analysis in the present study.

In the case of the placenta, however, the use of tetraploid complementation means that the extraembryonic compartment is derived predominantly from wild-type tetraploid cells, and is therefore not the most appropriate tissue for assessing transcriptional consequences of the bipaternal embryonic genome itself. This consideration led us to examine more closely how best to address the reviewer’s concern within the limits of the current model. We reasoned that mid-gestation embryos provide a particularly suitable material in this setting, because they include the developing brain while also capturing a broader range of embryonic tissues. In this sense, they provide a more comprehensive developmental readout that is also closely related to the organ-level analyses presented later in the manuscript. In addition, because this stage precedes later organ maturation and postnatal physiological divergence, it is likely to reflect imprinting-related developmental regulation more directly, with less superimposed influence from secondary downstream differences. This is also the developmental period during which imprinting-dependent expression and regulation are most broadly active before birth (e.g. Ferguson-Smith, 2011; Tucci et al., 2019).

Guided by these considerations, we extended the revised study by establishing an expanded E12.5 embryonic dataset with six biological replicates. This allowed us to examine developmental transcriptional states under the bipaternal condition using a material that addresses the reviewer’s concern directly, while also providing a broader and developmentally more informative view than analysis of brain tissue alone. The corresponding results are presented below.

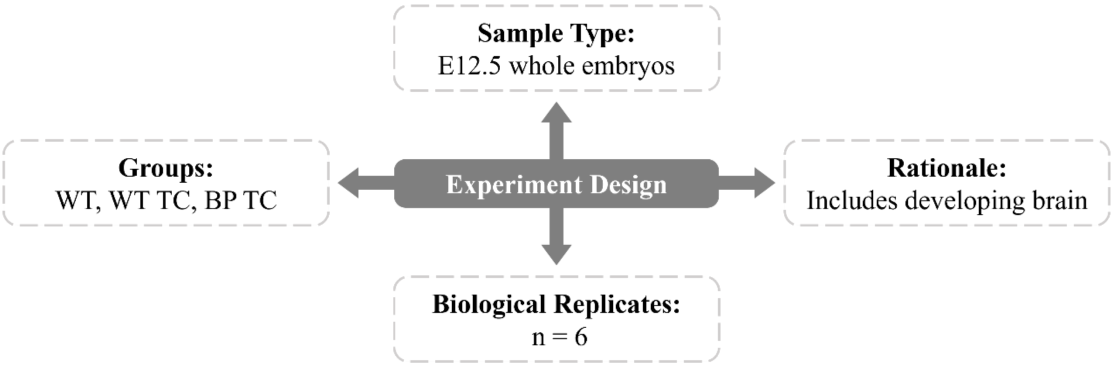

Panel 1. Expanded embryonic transcriptomic analysis using E12.5 embryos.

To address the reviewer's suggestion that imprinting-related effects should be examined in a developmentally informative tissue context, we established an expanded E12.5 embryonic RNA-seq dataset with six biological replicates per group. E12.5 embryos include the developing brain while also providing a broader embryonic tissue context in which imprinting-dependent regulation remains widely active before birth.

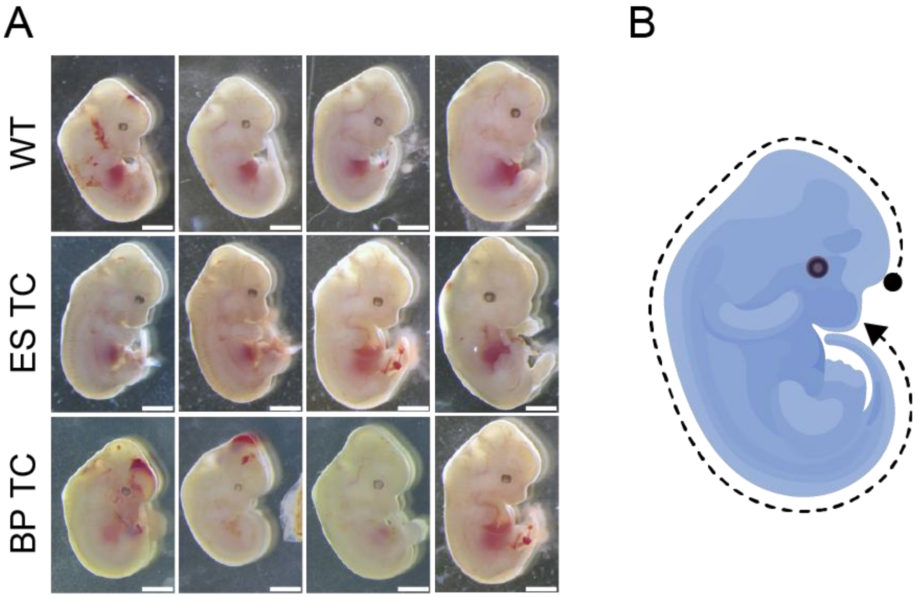

Panel 2. E12.5 embryos provide a developmentally informative material for transcriptomic analysis.

Representative images of E12.5 embryos and the body-length measurement strategy used in the revised study. This stage captures developmental variation before later organ maturation and postnatal physiological divergence.

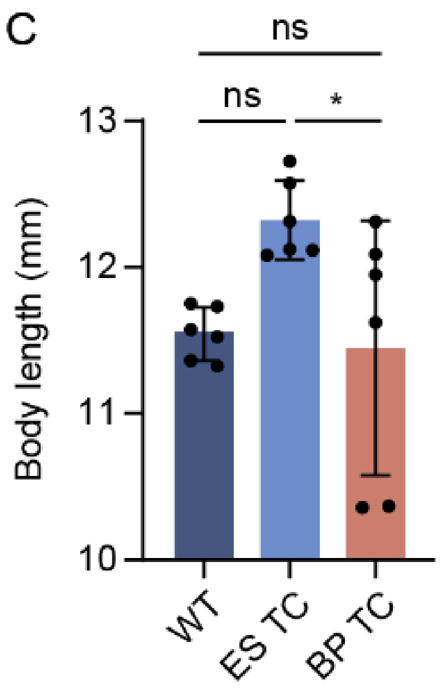

Panel 3. Morphological variation is already evident in BP embryos at E12.5. Quantification of body length at E12.5 shows that BP embryos display a broader developmental range than controls, indicating that divergence is already present at the embryonic stage.

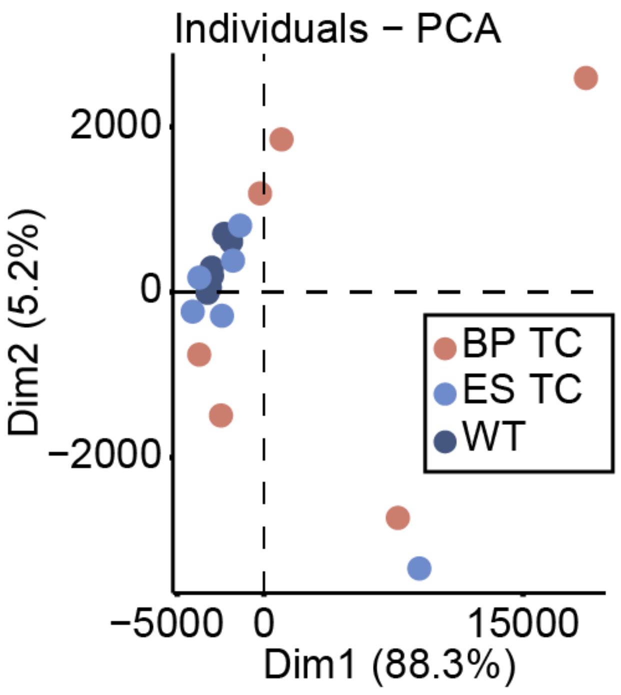

Panel 4. BP embryos occupy a broader transcriptional space at E12.5.

PCA of whole-embryo transcriptomes at E12.5 shows that BP embryos extend across a broader region of transcriptional space than WT and WT TC controls, indicating greater divergence among embryos.

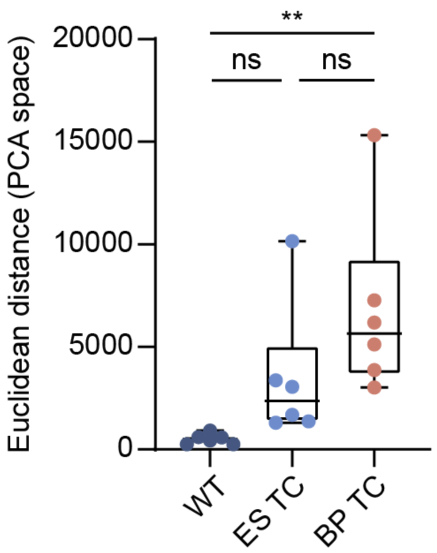

Panel 5. Quantitative analysis confirms increased embryonic transcriptomic divergence in BP mice.

Pairwise Euclidean distance analysis shows significantly greater within-group transcriptional distances among BP embryos than among WT embryos, whereas WT TC embryos do not show a comparable increase.

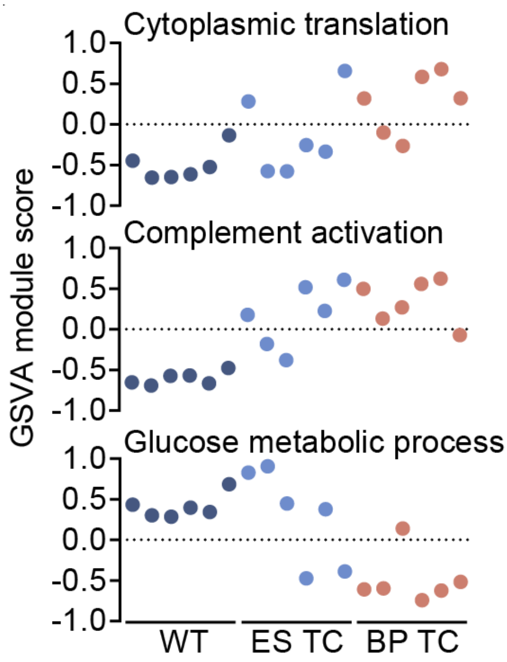

Panel 6. Developmentally relevant transcriptional programs show broader distributions in BP embryos.

Representative GSVA module scores illustrate that biological processes associated with essential cellular and metabolic activities vary more broadly among BP embryos than among controls.

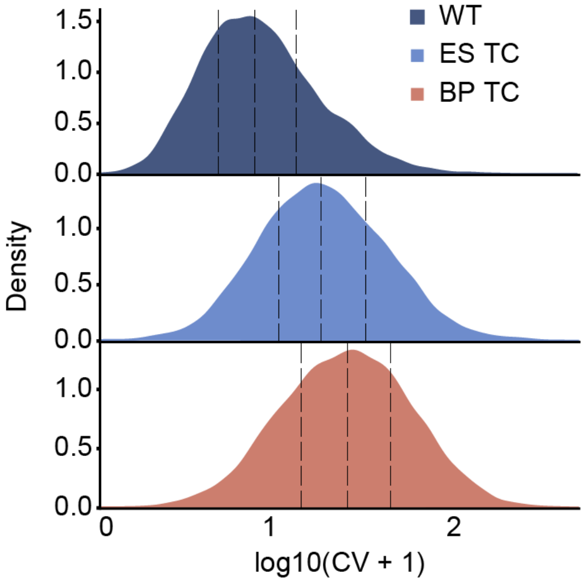

Panel 7. BP embryos show a broader distribution of gene-expression variability.

Gene-wise CV analysis across six biological replicates shows that BP embryos have the

highest overall variability distribution, indicating an expanded range of transcriptional states at the embryonic stage.

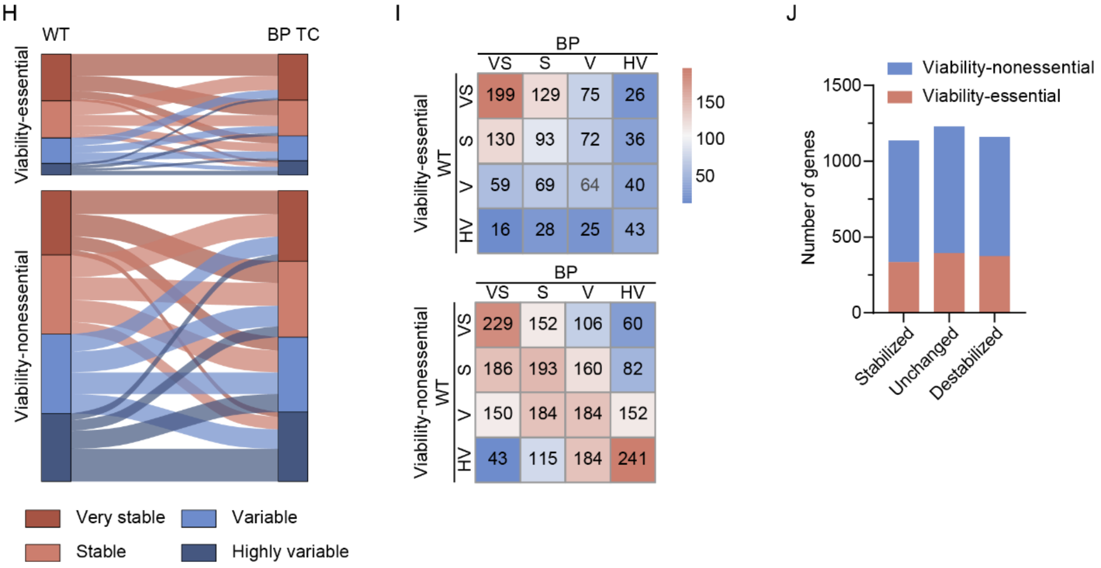

Panel 8. Transcriptional divergence in BP embryos extends to developmentally important genes.

Analysis of gene-stability transitions shows that the altered transcriptional state in BP embryos is not restricted to peripheral genes but also affects subsets of genes important for embryonic development.

To address the reviewer's concern that imprinting-related effects might be more appropriately examined in the brain or placenta, we revisited tissue choice in the context of the present experimental system. Because the placenta in tetraploid-complemented conceptuses is derived predominantly from wild-type tetraploid cells, it is not the most appropriate tissue for assessing transcriptional consequences of the bipaternal embryonic genome itself. We therefore established an expanded RNA-seq dataset using whole E12.5 embryos, with six biological replicates per group (Panel 1). This material includes the developing brain while also providing a broader embryonic context in which imprinting-dependent regulation remains widely active before birth. Representative images of E12.5 embryos and the body-length measurement strategy used in the revised study are shown in Panel 2. Quantification of embryonic body length

demonstrates that BP embryos already display a broader developmental range than controls at this stage (Panel 3). PCA of whole-embryo transcriptomes further shows that BP embryos occupy a broader transcriptional space than WT and WT TC controls (Panel 4), and this is confirmed quantitatively by pairwise Euclidean distance analysis (Panel 5). Representative pathway-level module scores indicate that core biological processes vary more broadly among BP embryos than among controls (Panel 6). Gene-wise CV analysis across six biological replicates shows that BP embryos have the broadest overall variability distribution, consistent with an expanded range of transcriptional states at the embryonic stage (Panel 7). Finally, stability-transition analysis shows that altered transcriptional states in BP embryos are not restricted to peripheral genes but also involve subsets of genes important for embryonic development (Panel 8). Together, these expanded embryonic analyses provide a developmentally informative assessment of transcriptional states under the bipaternal condition and are described in the revised Results section **"Broader developmental and transcriptional states in BP embryos during mid-gestation"** (corresponding to **Fig. 2A–J** in the revised manuscript).

-Tetraploid complementation is a very interesting approach, which is however expected to give variation between the obtained because of variable contributions of the diploid cells to the embryonic and extra-embryonic compartments. This may explain in part the considerable differences in gene expresion observed between the two BP animals.

Response:

We thank the reviewer for raising this important point regarding potential sources of variation in the tetraploid complementation system. This comment was particularly helpful because it drew our attention to the extent of divergence between individual BP samples in the original analysis and to the need to consider more carefully how the embryonic and extraembryonic compartments should be interpreted in this setting.

As described in the preceding responses, we first revisited the transcriptional differences observed between BP animals themselves. This led us to substantially

expand the transcriptomic analyses at both the organ and embryonic levels. By increasing the number of biological replicates in the multi-organ dataset and by establishing an expanded E12.5 embryonic dataset, we were able to examine more rigorously whether the differences initially observed between BP samples were best interpreted as stable group-level expression changes or as part of a broader reduction in convergence among individual BP transcriptomes. These expanded analyses showed that the divergence among BP samples is not limited to two individual animals, but instead reflects a broader developmental and transcriptional feature of the bipaternal condition.

At the same time, the reviewer's comment also led us to examine more closely the developmental context of tetraploid complementation itself. In the framework used here, ESC-derived cells form the embryo proper, whereas tetraploid cells contribute predominantly to extraembryonic tissues. We therefore considered it important to distinguish more carefully between variability that might arise in the extraembryonic context and the transcriptional differences observed in the embryonic and postnatal tissues analyzed in this study. This, in turn, led us to extend the placental analyses in the revised manuscript, including direct evaluation of placental contribution patterns and more detailed examination of whether BP ESCs might retain features relevant to extraembryonic contribution and broader developmental potency.

Taken together, these additional analyses allowed us to place the observed BP transcriptomic differences in a more appropriate developmental framework. In the revised manuscript, the resulting picture is no longer based simply on differences between a small number of BP samples, but on expanded analyses showing that BP conceptuses retain broader developmental and transcriptional heterogeneity even within the tetraploid complementation setting, while residual variation in the placental context may still contribute to this phenotype. The corresponding placental analyses are presented below.

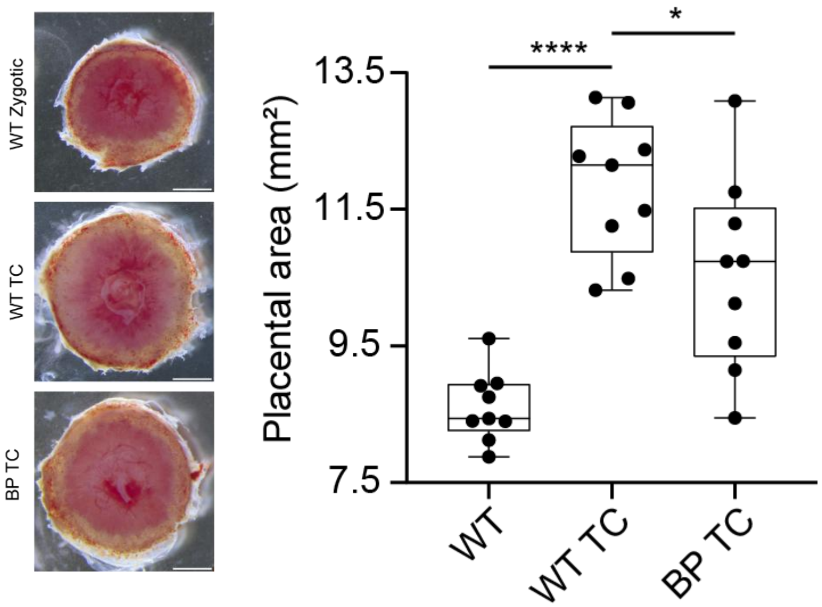

Panel 1. Placental abnormalities are alleviated but not eliminated under tetraploid complementation.

Representative E12.5 placentas and quantitative analysis show that BP ESC-derived conceptuses display smaller placentas than WT ESC TC conceptuses, but complete normalization is not achieved.

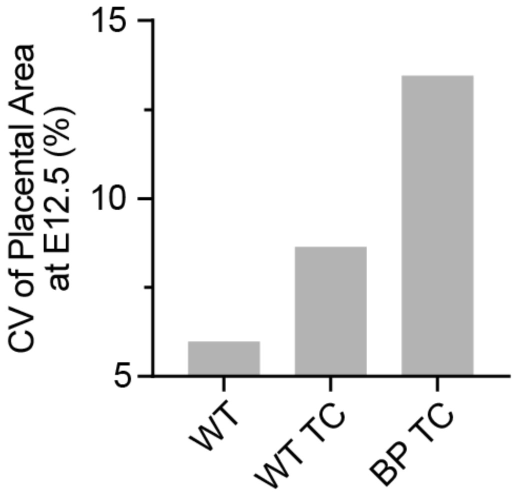

Panel 2. Placental size remains more heterogeneous in BP conceptuses under tetraploid complementation.

Coefficient-of-variation analysis shows a broader distribution of placental size in BP ESC-derived conceptuses than in WT ESC TC controls, indicating that residual

heterogeneity remains in the placental context.

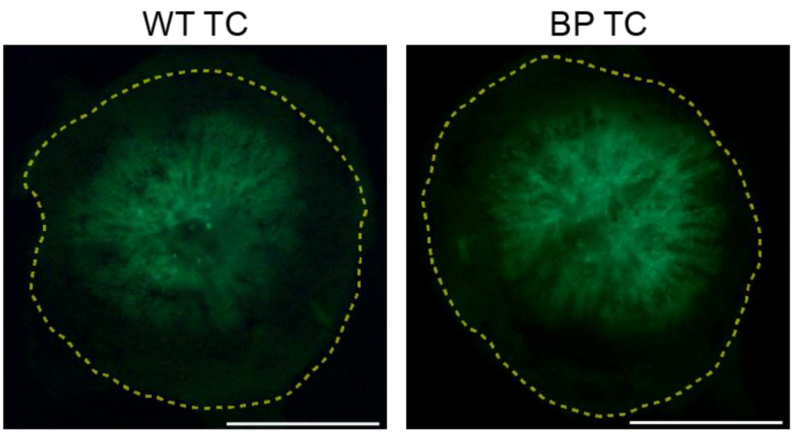

Panel 3. ESC-derived contribution to placental tissue was examined directly in WT TC and BP TC conceptuses.

Fluorescence imaging of E12.5 placentas shows GFP-positive ESC-derived cells on the fetal side of placentas in both WT ESC TC and BP ESC TC conceptuses.

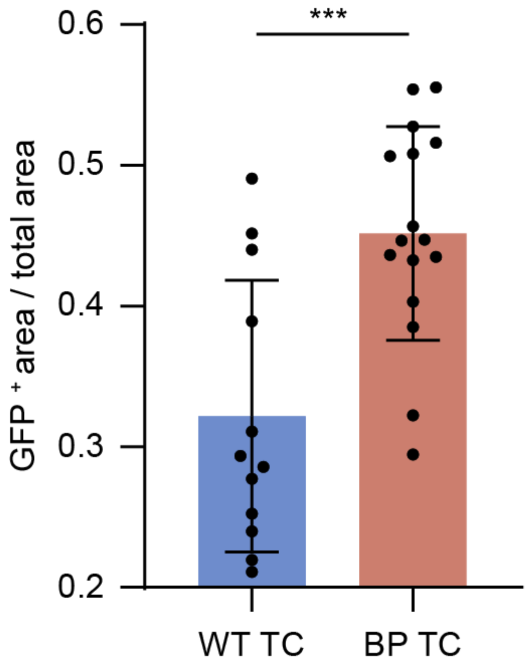

Panel 4. BP ESC-derived placentas show increased GFP-positive contribution relative to WT ESC TC controls.

Quantification of GFP-positive area relative to total placental area shows that ESC-

derived contribution is significantly higher in placentas associated with BP ESC-derived conceptuses than in those derived from WT ESCs.

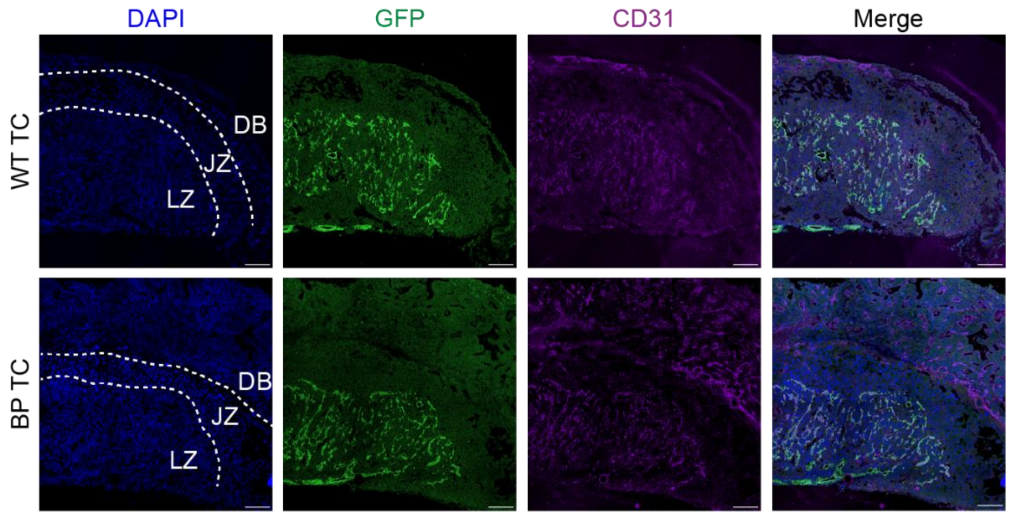

Panel 5. ESC-derived cells in BP placentas are restricted mainly to fetal endothelial components.

Immunofluorescence analysis shows strong overlap between GFP and CD31, indicating that ESC-derived cells detected in BP placentas are localized predominantly to endothelial cells within the labyrinth vasculature rather than being broadly distributed across trophoblast compartments.

To address the reviewer's concern that variability in the tetraploid complementation system might contribute to the differences observed between BP animals, we further examined the placental context in the revised study. Representative E12.5 placentas and quantitative analysis showed that placental abnormalities in BP ESC-derived conceptuses were alleviated but not fully normalized under tetraploid complementation (Panel 1), and coefficient-of-variation analysis indicated that placental size remained more heterogeneous in BP conceptuses than in WT ESC TC controls (Panel 2). We therefore examined ESC-derived contribution to placental tissues directly. Fluorescence imaging revealed GFP-positive ESC-derived cells on the fetal side of placentas in both WT ESC TC and BP ESC TC conceptuses (Panel 3), and

quantification showed that ESC-derived contribution was increased in BP placentas relative to WT ESC TC controls (Panel 4). However, immunofluorescence analysis showed that these GFP-positive ESC-derived cells were localized predominantly to CD31-positive endothelial cells within the labyrinth vasculature rather than being broadly distributed across trophoblast compartments (Panel 5). Together, these analyses indicate that residual heterogeneity persists in the placental context under tetraploid complementation and may still contribute to developmental variation in BP conceptuses, while the placental ESC-derived contribution observed here is restricted in anatomical distribution. These analyses are described in the revised Results section **"Tetraploid complementation improves the generation of bi-paternal mice and reveals early variability in placental development"** (corresponding to **Fig. 1D–I** in the revised manuscript).

-Overall, it is unclear to what extent the aberant expression of genes in the bipaternal animals is caused by the extensive manipulations that were used to generate them (oocyte derivation, embryo fusion and in vitro culture, injection of enucleated oocytes, etc). Unless this reviewer is mistaken, the control WT animals did not undergo all these in vitro manipulations, which are known from the literature to affect imprinted gene expression, particularly in the mouse. Therefore, the comparison between the BP and the WT does not allow one to draw conclusions.

<u>Response</u>:

We thank the reviewer for raising this important concern regarding the potential contribution of extensive in vitro manipulation to the transcriptional abnormalities observed in BP animals. We fully agree that, in this type of system, it is essential to distinguish effects associated with the bipaternal genomic condition from those that might arise from ESC derivation, culture, embryo manipulation, or tetraploid complementation itself.

This point became an important part of the revision. To address it more directly, we introduced an additional control group in the revised study using **wild-type ESC-**

derived tetraploid-complemented conceptuses and embryos. These controls underwent the same general experimental framework as the BP ESC-derived samples, including ESC culture and tetraploid complementation, while retaining a wild-type genotype. This allowed us to evaluate more directly whether the developmental and transcriptional features observed in BP mice could be explained primarily by the manipulation procedures themselves.

As now shown in the revised manuscript, these WT ESC-derived controls did not exhibit the same degree of developmental or transcriptomic divergence as the BP samples. This was evident both in the placental and embryonic analyses and, in particular, in the expanded E12.5 transcriptomic dataset, where WT TC embryos remained much closer to naturally fertilized WT controls than to BP TC embryos. These comparisons indicate that the broader divergence observed under the BP condition cannot be accounted for simply by the in vitro procedures alone.

For this reason, the revised study no longer depends only on comparisons between BP animals and naturally derived WT controls, but includes a more stringent procedural control framework as part of the central developmental analysis. The corresponding results are presented in the revised Results sections **"Tetraploid complementation improves the generation of bi-paternal mice and reveals early variability in placental development"** and **"Broader developmental and transcriptional states in BP embryos during mid-gestation"** (Fig. 1D–F; Fig. 2C–J).

-As concerns the haploid ESC line in which the authors had deleted the 20 ICRs, it is not shown to what extent these cells retain normal methylation levels, for instance at the secondary somatic DMRs, or genome-wide. Did their complex derivation and culturing under 2i conditions not affect these methylation patterns ?

Response:

We thank the reviewer for raising this point. The methylation state of the haploid ESC line used in this system is indeed central to understanding how the bipaternal model is established.

This issue was examined in our earlier study (Cell Stem Cell, 2018), on which the present work is built. In that study, androgenetic haploid ESCs cultured under naive 2i conditions progressively underwent extensive DNA hypomethylation and loss of imprinting, and acquired a PGC-like methylation profile. Imprinted regions that normally undergo demethylation during germline reprogramming showed correspondingly low methylation levels in cultured haploid ESCs, consistent with broader features of germline epigenetic resetting (Hackett et al., 2013). Similar global hypomethylation under naive culture conditions has also been described in diploid ESCs and related pluripotent states (Leitch et al., 2013). In our previous work, this methylation pattern was observed across the ahESC clones analyzed and in the embryos and bipaternal mice derived from them, which is why it became the epigenetic background on which the engineering strategy was developed.

In this sense, the methylation changes highlighted by the reviewer are part of the established context from which the bipaternal system is constructed. The engineered configuration used here depends on the fact that cultured ahESCs no longer retain an intact sperm-like imprinting state, thereby allowing defined deletions of parent-of-origin regulatory regions to function as an artificial replacement for aspects of the normal imprinting program.

Overall, we are grateful for the reviewer's comments, which led us to clarify several important aspects of the study more explicitly, including the interpretation of transcriptional alterations in relation to imprinted-gene networks, the need for expanded organ-level transcriptomic analyses with increased biological replication, the value of an additional embryonic-stage dataset, and the role of the placental context under tetraploid complementation. Together, these comments and the corresponding revisions substantially strengthened both the evidential basis and the overall framing of the manuscript.

Reviewer #2:

In this manuscript the authors provide a follow up to their impressive earlier experiments demonstrating that targeted deletion of 20 imprinting control regions enabled survival of bipaternal mice until adulthood. These important experiments demonstrated that the block to uniparental development largely results from imprinted genes. However, the bipaternal mice had substantial placental abnormalities, which limited survival. Here tetraploid aggregation was used to (largely) rescue the placental defects. Although survival was increased, the offspring still exhibited numerous transcriptional, DNA methylation and phenotypic abnormalities relative to wildtype mice. The authors conclude mammals exhibit parental asymmetry in addition to strictly imprinted genes, resulting in abnormal phenotypes. This is a very reasonable conclusion, given the data. Moreover, the additional phenotyping of the bipaternal mice is extremely informative. Nevertheless, given that imprinted gene

expression is still not completely normalized, it is difficult to exclude their role in the asymmetry.

Response:

We thank the reviewer for the careful reading of our manuscript and for the thoughtful evaluation of this study. We are grateful that the reviewer recognized both the significance of the earlier bipaternal work and the informative value of the additional phenotypic analyses presented here. We also appreciate the reviewer's important reservation that, because imprinted gene expression is still not completely normalized, the contribution of residual imprinting defects to the observed phenotypes cannot be readily excluded.

These comments were highly influential in shaping the present revision. In returning to the study with these points in mind, we re-examined not only the interpretation of the original findings, but also the extent of experimental support needed for the manuscript as a whole. This led us to carry out substantial additional experimental work and to incorporate multiple new analyses, with the aim of reassessing bipaternal development across a broader developmental and physiological framework.

As a result, the present manuscript is not simply a more narrowly worded version of the

original submission. It has been extensively expanded and reorganized on the basis of these additional experiments and analyses. The revised version now places greater emphasis on the developmental and physiological features that can be robustly characterized in bipaternal mice after placental defects are largely alleviated by tetraploid complementation, while also making the interpretive limits of the current model more explicit. This broader re-examination allowed us to retain what is most informative in the system while avoiding conclusions that extend beyond what the expanded data can support.

We respond to the reviewer's specific comments in detail below.

Comments:

1. I am a bit confused about the controls (WT) for BP experiments. Shouldn't biparental ES cells be used as the control if the goal is to determine if genes other than imprinted genes confer parental asymmetry? Otherwise, the BP embryos have the tetraploid placentas, which could be causing some of the post implantation phenotypic differences (metabolomics).

<u>Response</u>:

We thank the reviewer for this important comment. We agree that the choice of control is central to how the BP phenotypes should be interpreted, particularly when the aim is to distinguish effects associated with the bipaternal genomic condition from those potentially introduced by ESC derivation, tetraploid complementation, and the residual placental context. This concern prompted us to re-examine the experimental framework more rigorously and to revise both the analysis and the overall interpretation accordingly. In the revised manuscript, we no longer rely only on the original comparison with naturally fertilized WT animals. Instead, we introduced an additional procedure-matched control using wild-type ESC-derived tetraploid-complemented embryos/conceptuses, so that the BP condition could be evaluated against a control generated under the same general experimental framework. In parallel, we substantially expanded the embryonic analysis at mid-gestation and increased biological replication

in the postnatal organ-level analyses, allowing the comparison to be made on a broader and more robust basis than in the original version.

These additions changed the way we interpret the dataset. The revised comparisons indicate that the tetraploid-complementation framework itself can introduce a limited degree of variation, particularly at the placental level, but that this alone does not account for the broader developmental and transcriptional spread observed under the BP condition. At the same time, the new data also made us more cautious in how we describe this aspect of the study. We therefore do not present the observed variation as definitive proof that parental asymmetry per se is the direct mechanistic source of the broader distributions, nor do we claim that residual placental or residual imprinting-related influences have been completely excluded, especially for later postnatal phenotypes. Rather, the revised manuscript supports a more restrained conclusion: under a substantially improved control framework, BP conceptuses and embryos remain incompletely normalized and continue to occupy a broader range of developmental and transcriptional states than control embryos. This is the level at which we now frame the evidence throughout the revised title, abstract, Results, and Discussion.

Taken together, we believe this revision directly addresses the reviewer's concern in a more rigorous way than before. By adding a procedure-matched control, extending the analysis to a developmentally informative embryonic stage, and re-evaluating the data under increased replication, the revised manuscript is better positioned to distinguish procedure-associated effects from BP-associated developmental differences, while also presenting the interpretation within the limits of what the current evidence can firmly support.

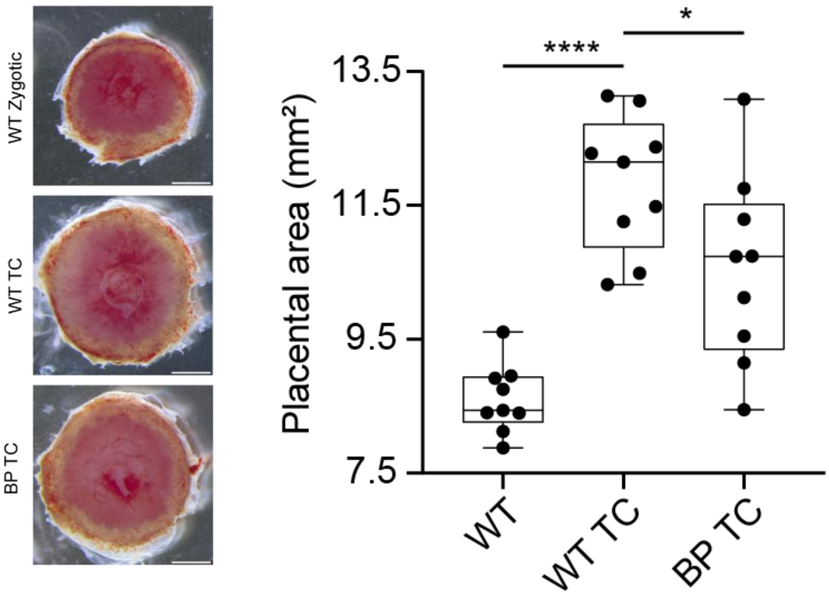

Panel 1. Placental abnormalities are alleviated but not eliminated under tetraploid complementation.

Representative E12.5 placentas and quantitative analysis show that BP ESC-derived conceptuses display smaller placentas than WT ESC TC conceptuses, but complete normalization is not achieved.

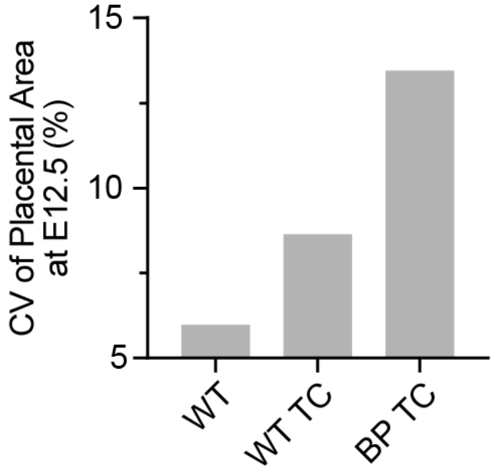

Panel 2. Placental size remains more heterogeneous in BP conceptuses under tetraploid complementation.

Coefficient-of-variation analysis shows a broader distribution of placental size in BP ESC-derived conceptuses than in WT ESC TC controls, indicating that residual heterogeneity remains in the placental context.

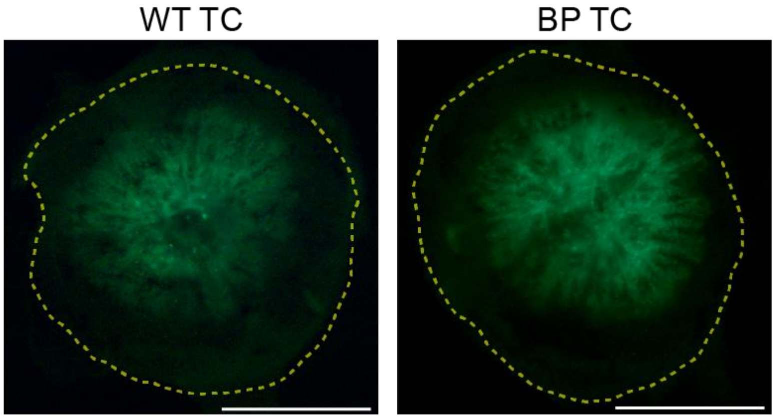

Panel 3. ESC-derived contribution to placental tissue was examined directly in WT TC and BP TC conceptuses.

Fluorescence imaging of E12.5 placentas shows GFP-positive ESC-derived cells on the fetal side of placentas in both WT ESC TC and BP ESC TC conceptuses.

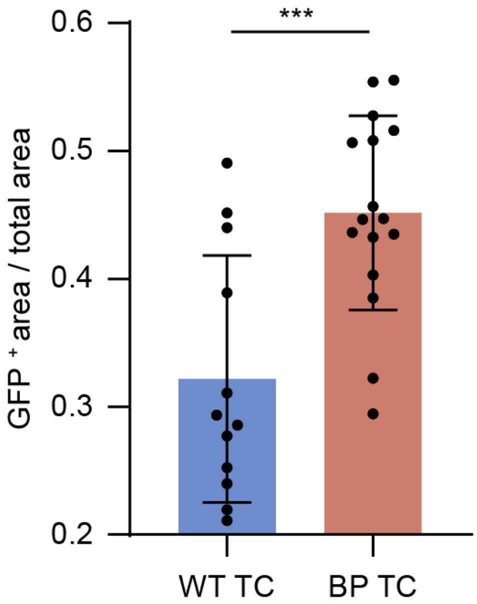

Panel 4. ESC-derived contribution is increased in BP-associated placentas.

Quantification of GFP-positive area demonstrates that placentas associated with BP ESC-derived conceptuses contain a significantly greater ESC-derived component than WT ESC TC placentas.

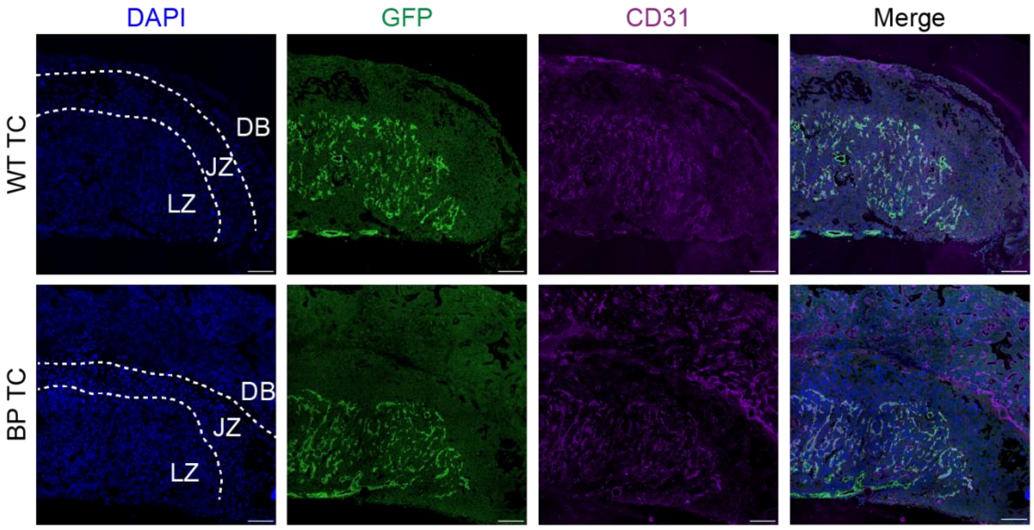

Panel 5. ESC-derived placental contribution is anatomically restricted to the fetal endothelial compartment.

Immunofluorescence analysis shows that GFP-positive ESC-derived cells in BP-associated placentas are localized predominantly within CD31-positive endothelial cells of the labyrinth vasculature, rather than being broadly distributed across trophoblast compartments.

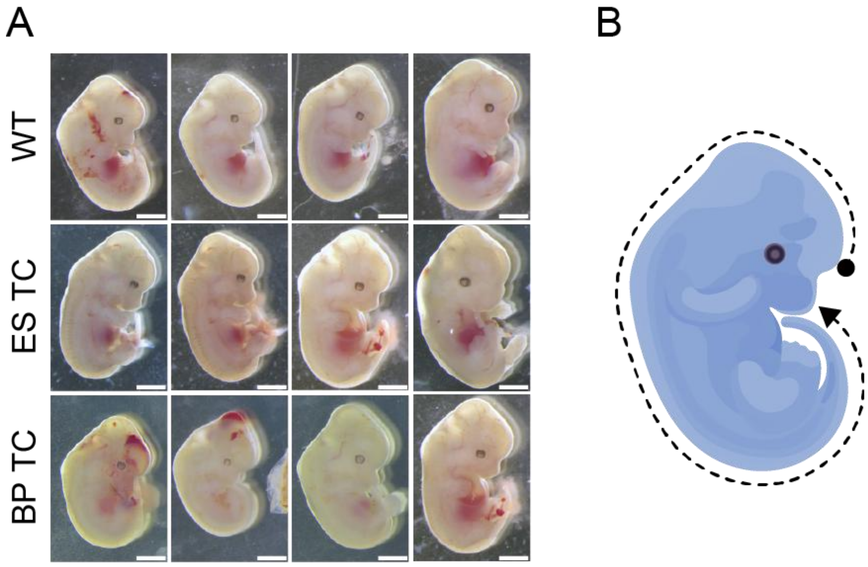

Panel 6. E12.5 embryos provide a developmentally informative material for transcriptomic analysis.

Representative images of E12.5 embryos and the body-length measurement strategy used in the revised study. This stage captures developmental variation before later organ

maturation and postnatal physiological divergence.

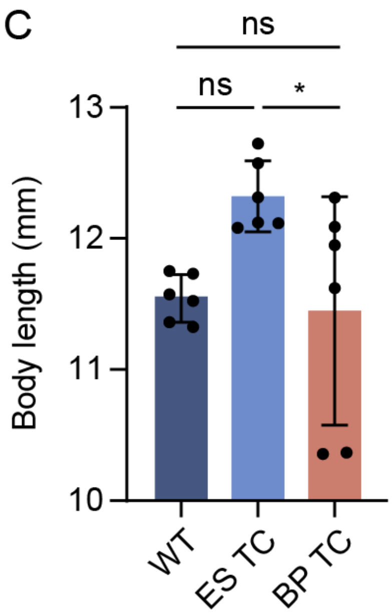

Panel 7. Morphological variation is already evident in BP embryos at E12.5.

Quantification of embryonic body length shows that BP embryos display a broader developmental range than controls, indicating that divergence is already present at the embryonic stage.

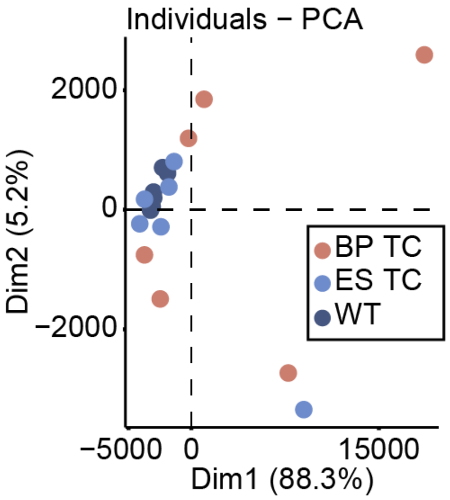

Panel 8. BP embryos occupy a broader transcriptional space at E12.5.

PCA of whole-embryo transcriptomes shows that BP embryos extend across a broader region of transcriptional space than WT and WT TC controls, indicating greater

divergence among embryos.

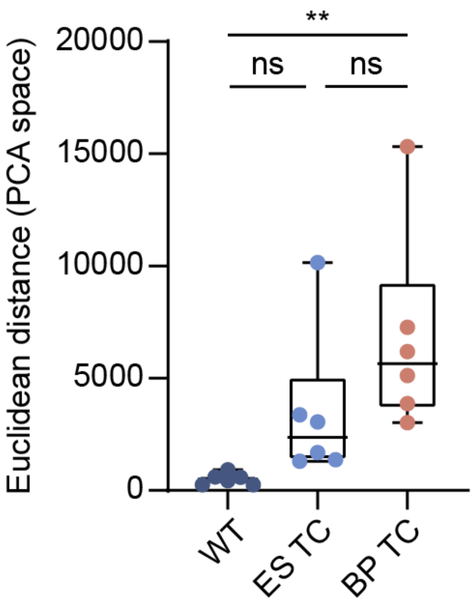

Panel 9. Quantitative analysis confirms increased transcriptomic divergence among BP embryos.

Pairwise Euclidean distance analysis shows that BP embryos exhibit greater within-group transcriptional divergence than WT embryos, whereas WT TC embryos do not show a comparable increase.

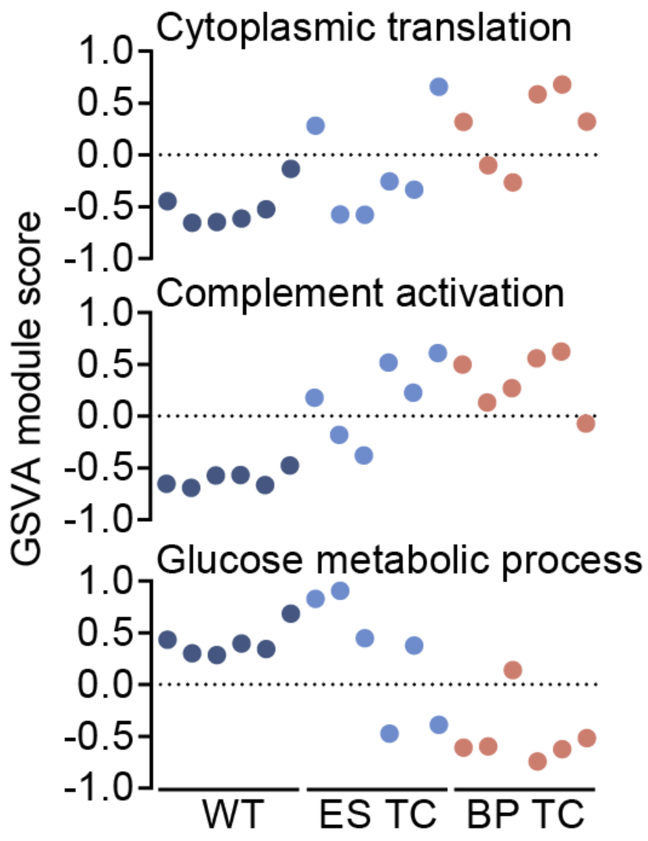

Panel 10. Developmentally relevant transcriptional programs show broader distributions in BP embryos.

Representative pathway-level module scores illustrate that core biological processes vary more broadly among BP embryos than among controls, indicating that embryonic divergence extends beyond global transcriptome structure.

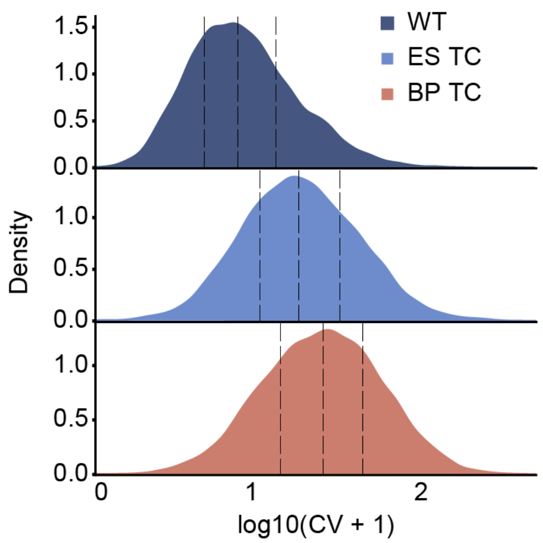

Panel 11. BP embryos show a broader distribution of gene-expression variability. Gene-wise CV analysis across six biological replicates shows that BP embryos have the broadest overall variability distribution, indicating an expanded range of transcriptional states at the embryonic stage.

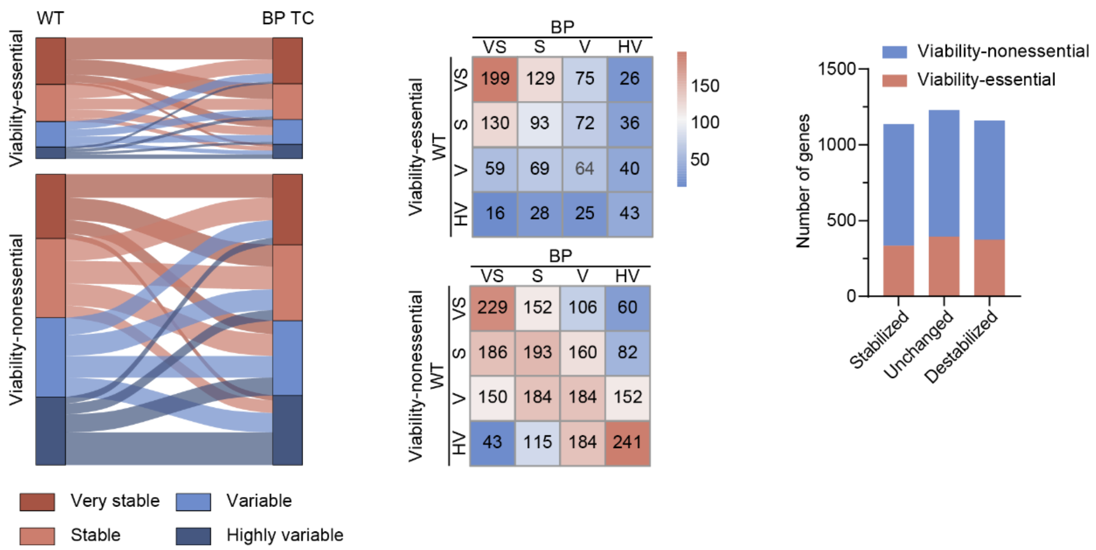

Panel 12. Transcriptional divergence in BP embryos extends to developmentally important genes.

Stability-transition analysis shows that altered transcriptional states in BP embryos are not restricted to peripheral genes but also involve subsets of genes important for embryonic development.

To address the reviewer's concern that variability in the tetraploid complementation

system might contribute substantially to the differences observed in BP animals, we re-examined this issue in a stepwise manner in the revised study. Representative E12.5 placentas and quantitative analysis showed that placental abnormalities in BP ESC-derived conceptuses were alleviated but not fully normalized under tetraploid complementation (Panel 1), and coefficient-of-variation analysis indicated that placental size remained more heterogeneous in BP conceptuses than in WT ESC TC controls (Panel 2). We therefore examined ESC-derived contribution to placental tissues directly. Fluorescence imaging revealed GFP-positive ESC-derived cells on the fetal side of placentas in both WT ESC TC and BP ESC TC conceptuses (Panel 3), and quantification showed that ESC-derived contribution was increased in BP placentas relative to WT ESC TC controls (Panel 4). However, immunofluorescence analysis showed that these GFP-positive ESC-derived cells were localized predominantly to CD31-positive endothelial cells within the labyrinth vasculature rather than being broadly distributed across trophoblast compartments (Panel 5). These placental analyses indicated that residual heterogeneity persists in the placental context under tetraploid complementation and may still contribute to developmental variation in BP conceptuses, while the ESC-derived placental contribution observed here remains anatomically restricted.

We then asked whether BP embryos still exhibited broader developmental and transcriptional divergence under this improved control framework. Representative E12.5 embryos and the body-length measurement strategy are shown in Panel 6. Quantification of embryonic body length showed that BP embryos already displayed a broader developmental range at E12.5 than controls, whereas WT ESC TC embryos remained largely comparable to naturally fertilized WT embryos (Panel 7). We therefore performed bulk RNA sequencing on whole E12.5 embryos. Principal component analysis showed that BP embryos occupied a broader transcriptional space than WT and WT ESC TC controls (Panel 8), and this pattern was confirmed quantitatively by pairwise Euclidean distance analysis, which showed greater within-group transcriptomic divergence among BP embryos without a comparable increase in WT ESC TC embryos (Panel 9). To determine whether this broader divergence

extended beyond global transcriptome structure, we further examined pathway-level transcriptional organization. Representative GSVA module scores showed that core biological processes varied more broadly among BP embryos than among controls (Panel 10). Gene-wise CV analysis across six biological replicates likewise showed that BP embryos exhibited the broadest overall variability distribution, whereas WT ESC TC embryos displayed only a modest rightward shift relative to WT embryos (Panel 11). Finally, analyses of essential and non-essential gene sets showed that this broader variability in BP embryos was not restricted to peripheral genes, but also involved subsets of genes important for embryonic development (Panel 12). Together, these analyses indicate that, under the same tetraploid-complementation framework, BP embryos remain incompletely normalized and occupy a broader developmental and transcriptional range than control embryos, whereas the procedural framework itself introduces only a more limited degree of variability.

These analyses are described in the revised Results sections "**Tetraploid complementation improves the generation of bi-paternal mice and reveals early variability in placental development**" and "**Broader developmental and transcriptional states in BP embryos during mid-gestation**" (corresponding to Fig. 1D–I and Fig. 2A–J in the revised manuscript). Consistent with these additions, the revised manuscript has also been adjusted at the interpretive level, so that the broader distributions observed in BP embryos are presented more cautiously throughout the revised title, abstract, Results, and Discussion.

2.There needs to be better characterization of the offspring. For example, Fig S1A shows fetuses and placenta. Were they weighed? A fetal/placenta ratio could be useful for assessing rescue with the tetraploid blastocysts.

Response:

We thank the reviewer for this helpful suggestion. Yes, fetal and placental weights were quantified in the revised study, and placental weight was further evaluated relative to fetal growth by normalizing placental weight to the mean fetal weight of each group.

As shown in Fig. S1A, this normalized placental-weight value progressively decreased with increasing extent of imprinting modification under tetraploid complementation, but remained significantly higher in 20KO BP conceptuses than in wild-type controls. Thus, tetraploid complementation substantially improved, but did not fully normalize, the fetal–placental relationship in BP conceptuses. This result also helped motivate the more detailed placental analyses presented in our response to Comment 1. These data have been incorporated into the revised Results section **"Tetraploid complementation improves the generation of bi-paternal mice and reveals early variability in placental development"** and are shown in Fig. S1A.

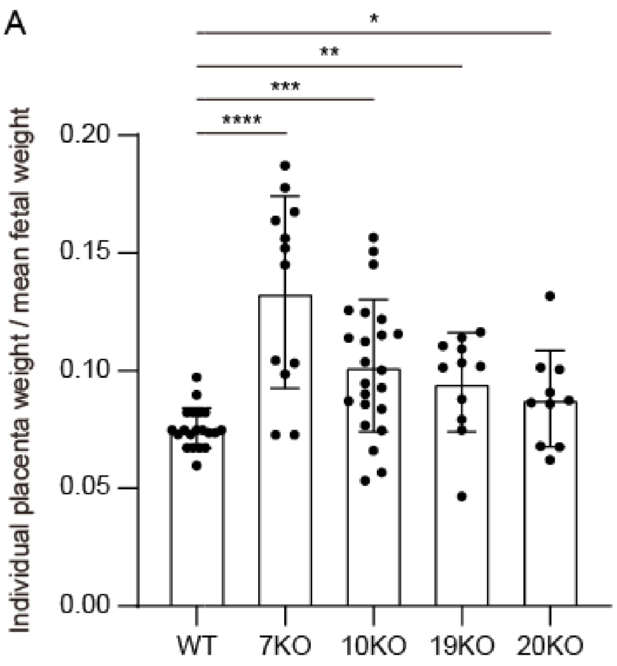

Fig. S1A. This analysis showed progressive improvement with increasing extent of imprinting modification, but the value in 20KO BP conceptuses remained significantly higher than that in wild-type controls. Placental weight normalized to the mean fetal weight of each group in WT, 7KO, 10KO, 19KO, and 20KO conceptuses generated under tetraploid complementation.

3.With respect to the RNAseq (Fig 1), it is somewhat surprising that the imprinted gene H19 exhibits such variable behavior. Did the authors filter genes by expression level? (failing to exclude lowly expressed genes could artificially overemphasize expression

changes).

Response:

We thank the reviewer for raising this important point. The variable behavior of H19 in the original RNA-seq presentation led us to re-check whether lowly expressed genes might have exaggerated some of the apparent expression changes. To minimize this possibility, genes were filtered by expression level before differential-expression analysis. In the E12.5 dataset, genes were retained only if they reached an FPKM value greater than 1 in at least 3 of the 6 biological replicates. The term-stage organ RNA-seq dataset was likewise filtered by minimum expression before downstream analysis, and these criteria are now stated explicitly in the revised Methods. These filtering steps were applied to reduce the possibility that very lowly expressed genes would artificially inflate fold changes or apparent variability. We therefore do not attribute the behavior of H19 simply to the inclusion of low-expression genes.

This comment also led us to reconsider how imprinted loci should be presented in the revised manuscript. Rather than emphasizing individual loci in isolation, the revised study places imprinted-gene behavior within the broader multi-organ transcriptomic framework. In this expanded analysis, imprinted genes that remained dysregulated in BP mice generally showed broader expression ranges across organs, together with wider transcriptomic dispersion at the organ level. We believe this framing is more appropriate for the current dataset and better reflects the level at which the evidence supports interpretation. These revisions are reflected in the revised Methods and in the Results section on multi-organ transcriptomic organization, particularly Fig. 3E and Fig. S4.

4.Figure 2B. The conclusion in the text is that BP mice are globally hypermethylated compared to WT. How was this experiment performed and where are the statistics? The methods state that whole-genome methylation sequencing data were aligned to the mouse. What does this mean?

Response:

We thank the reviewer for this important comment. This point led us to re-check both the analysis and the wording associated with this panel. After verification, we confirmed that the original statement reflected the direction shown in the original figure: BP samples displayed higher mean genome-wide methylation levels than WT in four of the five organs examined, with spleen as the exception. This pattern is also broadly compatible with the known difference between sperm- and oocyte-derived methylation states. At the same time, we agree that the original presentation was too compressed and that the wording was too categorical. In the revised manuscript, we therefore no longer use this panel to support an unqualified statement of "global hypermethylation," but instead describe it more cautiously as showing alterations in global DNA methylation levels across multiple tissues.

Whole-genome bisulfite sequencing was performed on liver, kidney, heart, spleen, and lung tissues from WT and BP mice. Reads were aligned to the mouse reference genome, methylated CpG sites were extracted, and the mean genome-wide methylation level was calculated for each sample and used for visualization in the revised figure. We agree that the original Methods wording ("aligned to the mouse") was too vague, and this has now been clarified. Regarding statistics, each organ in this WGBS dataset included only two biological replicates per genotype. We therefore do not interpret this panel as a strong organ-by-organ statistical demonstration, but rather as a descriptive overview of a modest, recurrent directional difference across multiple tissues. We believe this revised framing better reflects both the observed data and the limits of the experiment. These clarifications are reflected in the revised Results and Methods and in Fig. 3H of the revised manuscript.

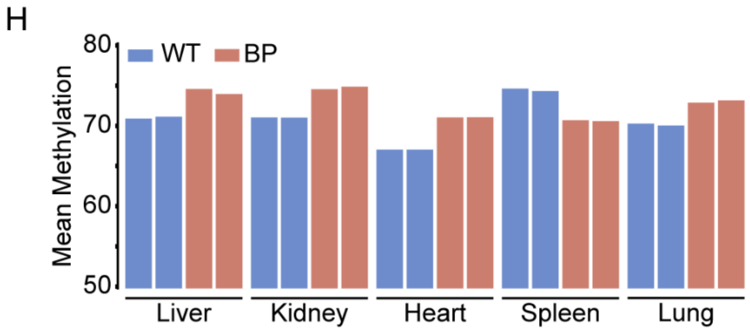

Fig. 3H. Global DNA methylation levels across five organs in term WT and BP mice,

presented as a descriptive overview of altered global methylation levels across multiple tissues rather than a definitive organ-by-organ statistical claim.

5.Figure 2C (DNA methylation of imprinted genes) is hard to understand. Can the authors come up with a more intuitive way of depicting these data?

Response:

We thank the reviewer for this constructive suggestion. We agree that the original presentation of methylation changes at imprinted loci was not sufficiently intuitive. In the revised manuscript, we therefore replaced the original summary-style display with a locus-level visualization. Specifically, revised Fig. 3I shows genome browser tracks of DNA methylation at the Mcts2-associated DMR across five organs at term, allowing the methylation pattern at a representative maternally methylated imprinted control region to be viewed directly in WT and BP samples.

Taken together with the preceding global methylation analysis, this result provides a more complete picture of the methylation landscape in BP mice. BP mice do not show a simple or indiscriminate methylation change. Instead, the data indicate a layered pattern, with a modest increase in genome-wide methylation across most organs together with reduced methylation at a specific maternally methylated imprinted DMR. These two features are consistent with the epigenetic properties expected from two sperm-derived genomes: at the global level, the overall methylation state of BP mice remains closer to the relatively higher methylation pattern characteristic of sperm, whereas at specific loci, maternally methylated imprinted regions show reduced methylation. In this sense, the revised figure provides a further clarification and confirmation of the epigenetic features of BP mice, and makes this aspect of the data much easier to appreciate than the original display. These revisions are reflected in the revised Results section **“Organ-specific transcriptional organization across multiple tissues in BP mice”** and in Fig. 3I of the revised manuscript.

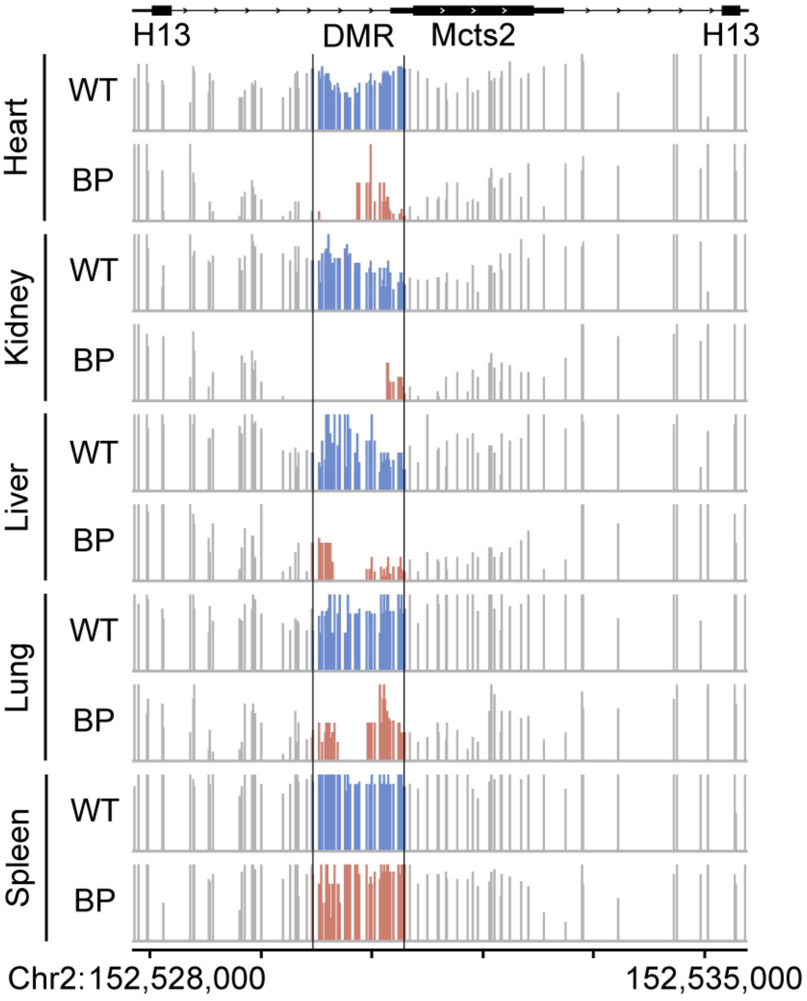

Fig. 3I. Representative DNA methylation profiles at the Mcts2-associated DMR across five organs at term, illustrating reduced methylation at a maternally methylated imprinted control region in BP mice.

6.In general, the methods are incomplete. It is very difficult to understand how the experiments were carried out.

Response:

We thank the reviewer for this important comment. We agree that the original manuscript did not explain the experimental workflow, particularly the tetraploid-complementation strategy, with sufficient clarity. In the revised manuscript, we therefore addressed this point at two levels. First, we substantially expanded the Methods throughout the manuscript, including the descriptions of the sequencing and

bioinformatic workflows questioned in the preceding comments. Second, we reworked the opening Results section and Fig. 1 so that the experimental design can be followed more directly. Specifically, revised Fig. 1A now provides a schematic of the BP tetraploid-complementation strategy, explicitly showing that BP ESC-derived cells form the embryo proper whereas tetraploid cells contribute predominantly to extraembryonic tissues. Fig. 1B shows that this strategy improves the recovery of BP pups, and Fig. 1C provides bright-field and GFP images of BP TC pups and their corresponding placentas, making the lineage configuration more immediately visible. To further clarify how this system behaves in placental tissues, we also added direct GFP-based analyses at E12.5: Fig. 1G–H shows ESC-derived contribution on the fetal side of the placenta, and Fig. 1I shows that this contribution is restricted mainly to CD31-positive endothelial cells within the labyrinth vasculature rather than broadly across trophoblast compartments. Consistent with the tetraploid-complementation framework, GFP fluorescence was present throughout the BP TC pup but remained mosaic and much more limited in the placenta, indicating that the embryo proper was generated predominantly from BP ESCs whereas placental contribution from BP-derived cells was restricted. In addition, embryonic analysis at E12.5 is now presented more explicitly, including the measurement scheme used for embryo phenotyping and the downstream transcriptomic analyses under this framework. Together, these revisions make both the experimental design and its interpretation substantially clearer than in the original submission. The detailed placental and embryonic analyses generated under this clarified framework are described in our responses to Comments 1 and 2, and the corresponding revisions are incorporated into the revised Methods and the Results section "**Tetraploid complementation improves the generation of bi-paternal mice and reveals early variability in placental development**."

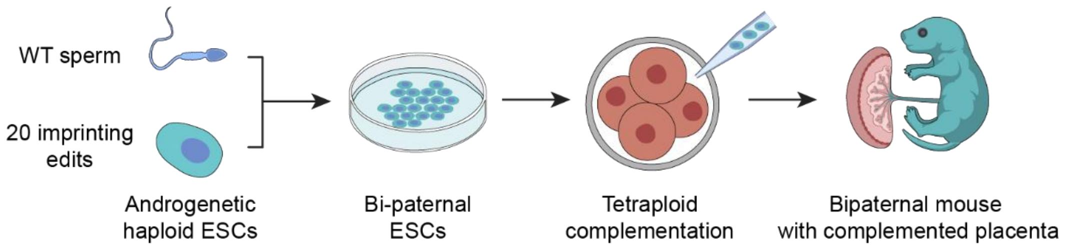

Fig. 1A. Schematic illustration of the tetraploid-complementation strategy used to generate BP TC mice, showing BP ESC contribution to the embryo proper and tetraploid contribution to extraembryonic tissues.

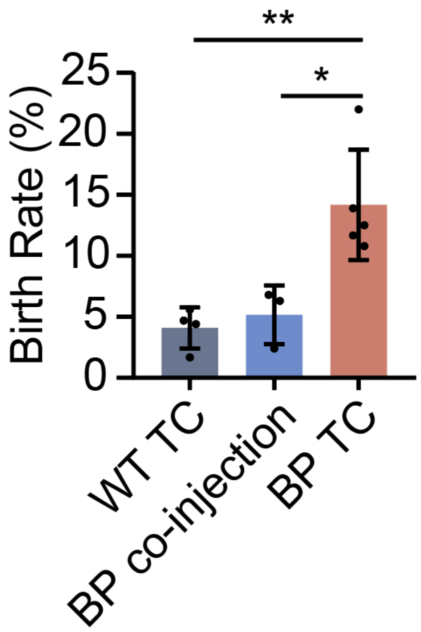

Fig. 1B. Comparison of birth rates among experimental groups. Bar graph showing the percentage of transferred embryos that yielded live-born pups in the WT TC, BP co-injection, and BP TC groups. Data are presented as mean ± SD, with individual data points representing independent embryo transfer experiments. Statistical significance is indicated.

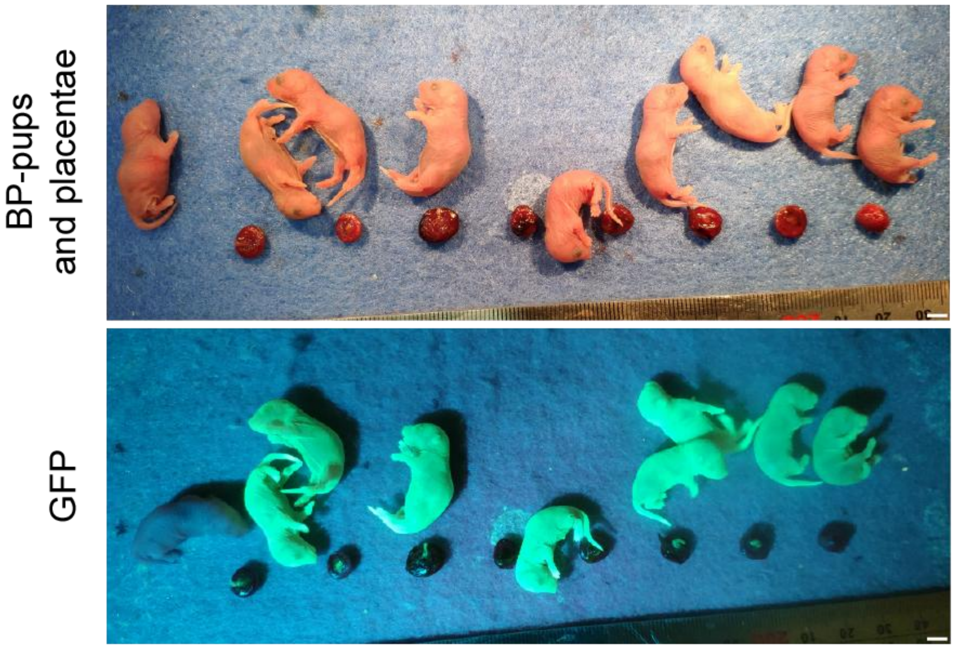

Fig. 1C. Bright-field and GFP images of BP TC pups and corresponding placentas, providing a direct visual illustration of ESC contribution in the tetraploid-complementation system.

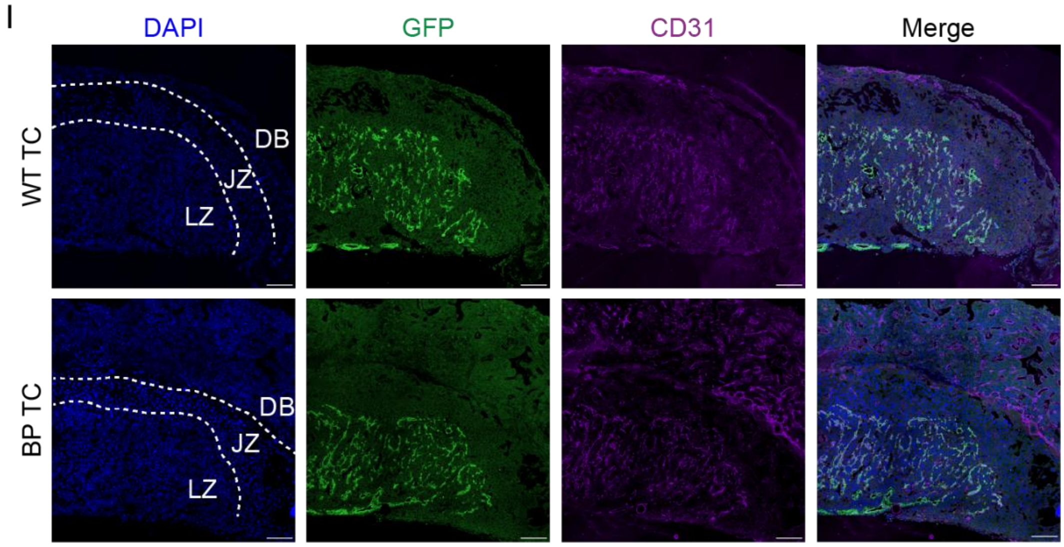

Fig. 1G–I. Direct examination of ESC-derived placental contribution at E12.5, including GFP-positive area quantification and CD31 co-staining showing restriction of ESC-derived cells mainly to fetal endothelial cells within the labyrinth vasculature.

We thank the reviewer for the thoughtful and constructive evaluation of our work. The

questions raised here prompted us to re-examine several central aspects of the study more rigorously, leading to extensive additional experiments, expanded analyses, and substantial revision of both the data presentation and the overall interpretation. In particular, these comments drove major improvements in the control framework, placental and embryonic characterization, and methodological clarity, which together have significantly strengthened the rigor, clarity, and evidential basis of the manuscript. We are therefore grateful for the reviewer's careful assessment, which led to important improvements throughout the revised study.

Reviewer #3:

[identifies himself as Gavin Kelsey]

The manuscript seeks to demonstrate that functional non-equivalence of the parental genomes in mice extends beyond genomic imprinting. The study uses elegant embryo construction approaches that build on previous work from the group (in particular ref. 11) that 'overcome' imprinting by engineering deletions at 20 imprinted loci, mostly at imprinting control regions (ICRs). The current manuscript presents an advance on the earlier report by improving embryo survival to term by use of tetraploid complementation (although tetraploid complementation was used in the previous report, it appears not to have been used specifically in the context of the 20KO ahESCs). The focus of the current manuscript is then to undertake transcriptome analysis on a set of nine tissues in neonates, complemented by genome-wide DNA methylation analysis, as well as metabolomic analysis in neonatal liver. The authors find substantial transcriptional deregulation in these neonatal tissues, very modest
global DNA hypermethylation (in 4 out of 5 tissues), and metabolomic changes, in particular within the TCA cycle. In addition, they report abnormal lipid accumulation in liver.

There is considerable technical ingenuity in the creation of viable 'biparental' (BP) mice with the manipulation of 20 imprinted loci. And the metabolomic analysis appears to

be well powered (8 replicates). On the other hand, the transcriptomic and methylome analysis is seriously underpowered. And it is difficult to see that the central contention that the phenotypes observed are independent of imprinting can be confidently supported because of a number of caveats not considered by the authors.

Response:

We thank the reviewer for the careful reading of our manuscript and for the highly thoughtful evaluation of this study. We are grateful that the reviewer recognized both the technical ingenuity involved in generating viable bipaternal mice through extensive manipulation of imprinted loci and the strength of the metabolomic analysis. We also fully appreciated the reviewer's central concerns, particularly regarding whether the scope of the original conclusions was adequately supported, whether the transcriptomic and methylome analyses were sufficiently powered, and whether residual imprinting-related effects or other features of the experimental system could be excluded as contributors to the observed phenotypes.

These comments were particularly influential in shaping the revision because they led us to re-examine not only individual datasets, but also the evidential basis of the manuscript as a whole. In response, we substantially expanded the study by introducing additional controls, increasing biological replication, and extending the analysis to a more informative embryonic stage, and we revised the structure and interpretation of the manuscript accordingly. This process did not simply narrow the wording of the original submission; rather, it changed the way we assessed the system itself.

A particularly important consequence of this revision was that, under the expanded control framework and increased replication, we were able to examine matched BP individuals more rigorously than before. In doing so, we found that individuals sharing the same engineered genotype nevertheless occupied a substantially broader range of developmental and transcriptional states, whereas the corresponding procedure-matched controls did not show a comparable degree of dispersion. This, in turn, led us to reconsider a recurrent but previously unintegrated observation from our earlier experimental experience: in our prior bipaternal studies, animals generated under the

same edited configuration often differed strikingly in the severity of developmental abnormalities or in whether postnatal survival was achieved, and a similar heterogeneity was also evident in the classic bimaternal study of Kono and colleagues. We do not present this as a retrospective interpretation imposed on the data. Rather, it was the reviewer's insistence on stronger support—especially the need for expanded replication and a more rigorous control framework—that allowed this feature of the system to emerge clearly enough to shape the revised manuscript.

For this reason, the revised manuscript is not simply a more cautious restatement of the original submission. It has been substantially expanded and reorganized, and the interpretation has been deliberately brought back to a more descriptive level. Rather than presenting the study as definitive evidence that parental genomes exert non-redundant functions beyond imprinting, we now focus on what the revised data can robustly support: after placental defects are largely alleviated by tetraploid complementation, BP conceptuses, embryos, and mice remain incompletely normalized and occupy a broader range of developmental and transcriptional states, with later physiological readouts converging toward a distinct metabolic configuration. Throughout the revised manuscript, we therefore present these findings more cautiously and explicitly within the limits of the current model. We respond to the reviewer's specific comments in detail below.

1. It is stated in the Introduction that "we selected androgenetic haploid embryonic stem cells (ahESCs) carrying deletions at all 20 critical imprinting control regions, a genotype previously shown to provide the most complete imprinting correction and to support adult BP offspring [11]."

In my reading of the earlier study (ref. 11), the deletions comprised 13 ICR deletions, with the remaining 7 being deletions, presumably in the coding regions, of 4 non-canonical imprinted loci (Gab1, Sfmbt2, Jade1, Smoc1), the Sfmbt2-encoding miRNA cluster, Igf2 and Xist. The original article refers to '20 key imprinted loci' rather than '20 critical imprinting control regions'.

The authors must revert to the original description of the deletions, and not to give the

impression that 20 ICRs were deleted.

There are many methodological details missing in the manuscript.

Response:

We agree that our original wording was inaccurate and could give the impression that all 20 deletions were located at imprinting control regions. This resulted from an overly compressed description in the opening text, where we used “imprinting control regions” as a shorthand for the broader set of imprinting-related modifications examined in our previous study. In the revised manuscript, we have therefore reverted to the original terminology and clarified the composition of these modifications in the opening sentence of the Results section, which now reads: *“In our previous work, deletions at 20 key imprinted loci enabled the generation of bi-paternal (BP) mice by co-injecting androgenetic haploid embryonic stem cells (ahESCs) together with sperm into enucleated oocytes. These modifications comprised 13 imprinting control regions and 7 additional imprinted genes or loci.”*

The reviewer is also correct that methodological details were insufficiently presented in the original submission. More broadly, the earlier version did not separate clearly enough between the primary observations and the scope of interpretation they could support. In the revised manuscript, we have therefore substantially reorganized the study from two parts into four. This restructuring gives much greater prominence to the experimental and developmental framework itself, while also allowing the placental and embryonic comparisons—both underdeveloped in the previous version—to be presented as distinct parts of the study. As a result, the revised manuscript devotes substantially more space to the methodological basis of the work and presents the overall logic in a form that more closely matches the evidential support.

2. The control group for the BP mice are described as WT. What are the WTs? I am assuming that they are unmanipulated ESCs (C57-GFP-ES?) that have also undergone tetraploid complementation, but this needs to be explicit.

In Table 1 it is notable that only 1.67% of transferred C57-GFP-ES cell-derived transferred embryos survived to term. Given this very low recovery rate, it has to be recognised that the WTs themselves have developed in an aberrant maternal environment of very small litters - litter size will impact growth trajectory and quite likely aspects of perinatal physiology.

It also possible that culturing even WT ESCs, in particular under 'hypomethylating' conditions such as 2i media, can lead to erosion of methylation at ICRs (e.g., PMID: 29356242); therefore the fidelity of imprinting in the WTs is not guaranteed.

My assumption from the previous study (ref. 11) is that neonates have been taken by caesarean section from surrogate females at e19.5. This should help mitigate against variation in gestation length. Nevertheless, having to compare between separate WT and BP litters clearly introduces a variable, for which sample numbers of a sufficient size is probably the best design to minimise confounding variables.

Response:

The reviewer is correct that the control framework in the original submission was not sufficiently explicit and, in part, not sufficiently rigorous for the scope of interpretation we were attempting to draw. In the context of developmental efficiency and birth-rate comparison, the WT group referred to unmanipulated ESC-derived conceptuses generated by tetraploid complementation. However, for the transcriptomic comparisons in the original version, we had relied primarily on naturally derived wild-type mice rather than including a procedure-matched ESC-derived tetraploid-complemented control. We agree that this left several variables insufficiently resolved, including the possible contributions of ESC derivation, 2i culture, tetraploid complementation, small litter size, and comparison across separate litters.

Rather than attempting to defend the original comparison framework, we redesigned it. In the revised manuscript, we established a new embryonic dataset at a strictly defined developmental stage (E12.5), including naturally fertilized WT embryos, wild-type ESC-derived tetraploid-complemented embryos (ES TC), and BP ESC-derived tetraploid-complemented embryos (BP TC), with six biological replicates per group.

This design allowed us to examine, within the same developmental window and under increased replication, which features are associated with the BP condition itself and which may instead reflect ESC culture or the tetraploid-complementation procedure. Importantly, the revised analyses showed that ES TC embryos did not display the same degree of developmental or transcriptomic divergence as BP embryos, although they were not entirely identical to naturally fertilized WT controls. We consider this an important result in its own right, because it is consistent with the reviewer's concern that ESC derivation and tetraploid complementation can introduce a limited degree of variation, while also showing that these procedure-associated effects are not sufficient to account for the broader divergence observed in the BP condition.

This change also altered how we interpret the study as a whole. The embryonic comparison now provides the developmental foundation for the later organ-level analyses, rather than leaving the postnatal WT-versus-BP comparison to carry that interpretive weight on its own. Accordingly, the revised manuscript no longer uses the original control framework to support broad mechanistic conclusions. Instead, under a substantially improved comparison framework, we now present the data at a level more closely matched to what they support: BP embryos remain incompletely normalized and occupy a broader range of developmental and transcriptional states than control embryos. The central embryonic analyses added in response to this concern are presented below in revised **Fig. 2A–J** and incorporated into the Results section **"Broader developmental and transcriptional states in BP embryos during mid-gestation."**

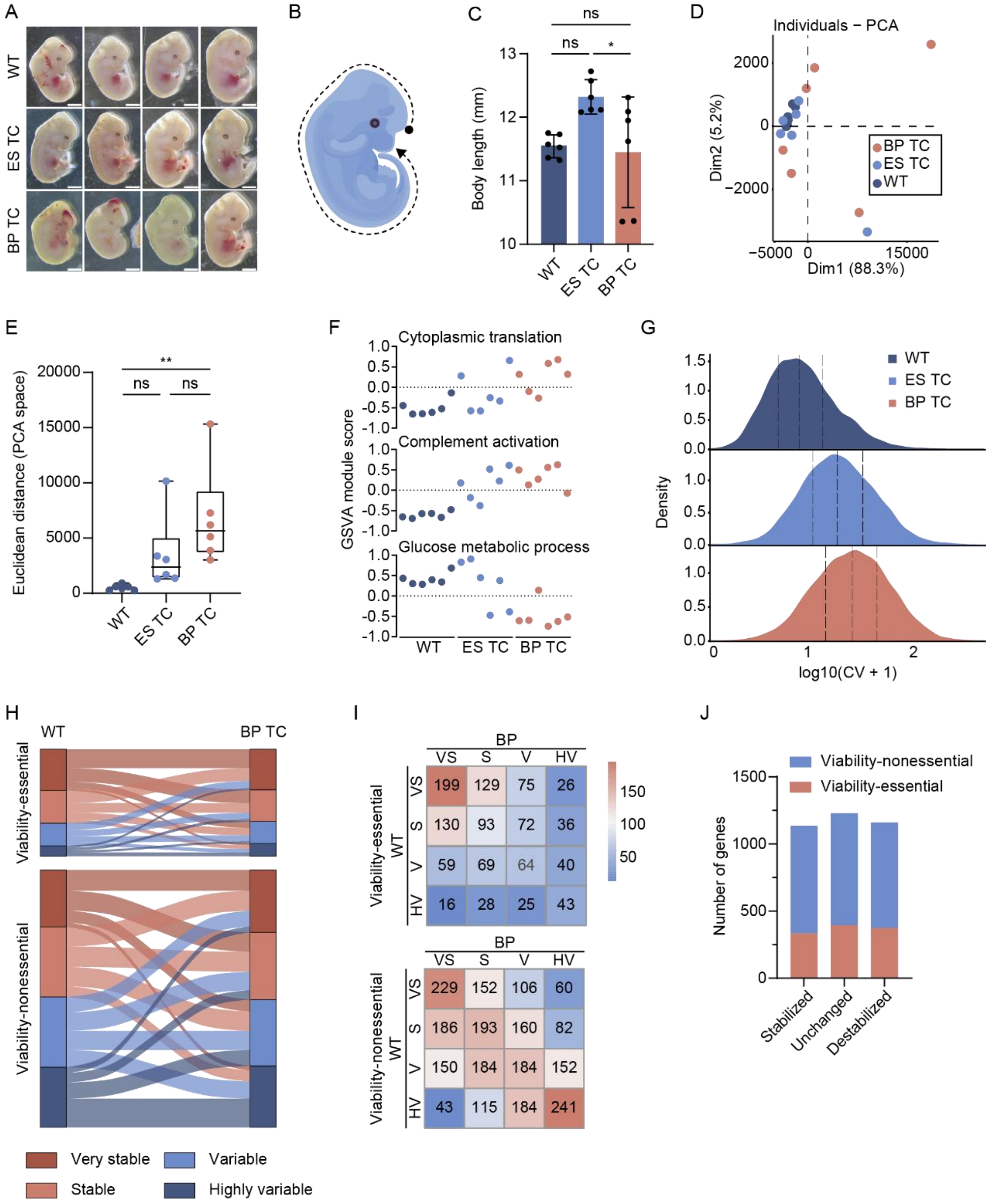

Revised Fig. 2A–J. Expanded embryonic analysis under an improved control framework.

To address the reviewer's concern that the original WT comparison did not fully control for ESC culture and tetraploid complementation, we established a new whole-embryo RNA-seq dataset at E12.5 using three groups analyzed within the same developmental window: naturally fertilized WT embryos, wild-type ESC-derived tetraploid-complemented embryos (ES TC), and BP ESC-derived tetraploid-complemented embryos (BP TC), with six biological replicates per group. Panels **A–C** define the

embryonic material used for this revised analysis and show that BP embryos already display broader morphological variation at mid-gestation. Panels **D–E** show that BP embryos occupy a broader transcriptomic space than either WT or ES TC controls, and that this difference is quantitatively reflected in greater within-group dispersion. Panel **F** further shows that representative biological programs vary more broadly across BP embryos than across controls. Panel **G** extends this observation genome-wide, showing that BP embryos have the broadest overall distribution of gene-expression variability, whereas ES TC embryos show only a modest shift relative to naturally fertilized WT embryos. Panels **H–J** show that this broader variability in BP embryos is not confined to peripheral genes, but also involves subsets of genes important for embryonic development. Together, these analyses distinguish the limited variation associated with the ESC/tetraploid-complementation framework from the broader developmental and transcriptional spread observed under the BP condition.

These analyses provide the revised developmental comparison added in response to the reviewer's concern regarding the original control framework. By incorporating both naturally fertilized WT embryos and procedure-matched ES TC embryos at E12.5, the revised design allows the BP condition to be evaluated against controls that separate, to the extent possible within the current model, the effects of ESC derivation and tetraploid complementation from the broader developmental and transcriptomic divergence observed in BP embryos. In the revised manuscript, this embryonic dataset is used as the developmental foundation for the later organ-level analyses, and the resulting conclusions are presented more cautiously and descriptively than in the original version. These analyses and the corresponding interpretive revisions have been integrated into the revised Results, figure legends, and the broader revised manuscript.

3. An 'n' of 2 samples per tissue for the RNA-seq and WGBS is insufficient to undertake robust statistical analyses - especially if the experimental and control samples are not from the same litters. In Fig. S1C, the separation of the two replicates of the same group is often of a similar magnitude as the separation between WT and BP samples,

indicating a considerable within group variation.

Response:

We agree with the reviewer that two biological replicates per tissue were not sufficient for robust statistical analysis of either the RNA-seq or WGBS datasets, particularly in a system where experimental and control samples were not derived from the same litters. We also agree that the degree of separation between replicates in the original PCA plots raised an important concern: with the original dataset, it was difficult to determine whether the observed spread primarily reflected underpowered sampling or a biologically meaningful feature of the BP condition.

In the original submission, our intention had been to survey a relatively broad organ range, and the RNA-seq analysis therefore included multiple postnatal tissues despite the limited replicate number. However, the reviewer is correct that this design provided breadth without sufficient replication to support the level of interpretation we were attempting to draw. In response, we expanded the organ-level transcriptomic analysis across the same postnatal tissues from two to four biological replicates per group. This substantially enlarged the organ-level dataset and allowed a more reliable assessment not only of differential expression across tissues, but also of how tightly organ transcriptomes converge among individuals within each genotype.

Under this expanded design, transcriptional differences between BP and WT tissues remained detectable across multiple organs. At the same time, increasing biological replication made a second feature much clearer than in the original version: across several organs, BP samples occupied a broader transcriptional space and showed greater within-group dispersion than WT controls. At the gene-expression level, broader expression ranges were also observed across a substantial fraction of the transcriptome, and representative pathway-level programs showed wider distributions among BP individuals rather than a single uniform shift. In the revised manuscript, we therefore present these results more cautiously, not as the basis for a strong mechanistic claim, but as evidence that organ-level transcriptional states remain less tightly convergent among BP individuals after birth. This emphasis is also more consistent with the revised

embryonic analyses, where broader developmental and transcriptomic dispersion is already detectable at E12.5 under an improved control framework.

This more restrained interpretation also fits better with the broader logic of the revised manuscript. In the bipaternal system, animals sharing the same engineered background have long shown marked differences in postnatal outcome even after major perinatal abnormalities were substantially alleviated, and the revised organ-level transcriptomic analysis now provides a more structured way to describe this feature at the tissue level. We do not present this observation as proof of a broader causal mechanism than the data can support. Rather, under increased replication, it now becomes possible to describe more rigorously that BP mice occupy a broader range of organ-level transcriptional states across individuals after birth. This descriptive level also places the transcriptomic results in better continuity with the later metabolic analyses, where adult BP liver shows a comparatively more coherent but abnormal physiological endpoint. In other words, the revised manuscript no longer treats the organ RNA-seq dataset as support for an overextended conclusion; instead, it uses the expanded replication to show more carefully how broader transcriptomic dispersion across individuals relates to the developmental and physiological organization of the BP condition.

Finally, we also revised the presentation of the WGBS data in light of this comment. Because the methylome analysis remained limited in replicate number, we no longer use it as the basis for strong organ-by-organ conclusions. In the revised manuscript, these data are presented more conservatively as supportive epigenetic context for the broader transcriptional observations. The central organ-level analyses added and reorganized in response to this concern are presented below in revised **Fig. 3A–I** and integrated into the Results section **"Multi-organ transcriptomic organization and DNA methylation profiles in BP mice."**

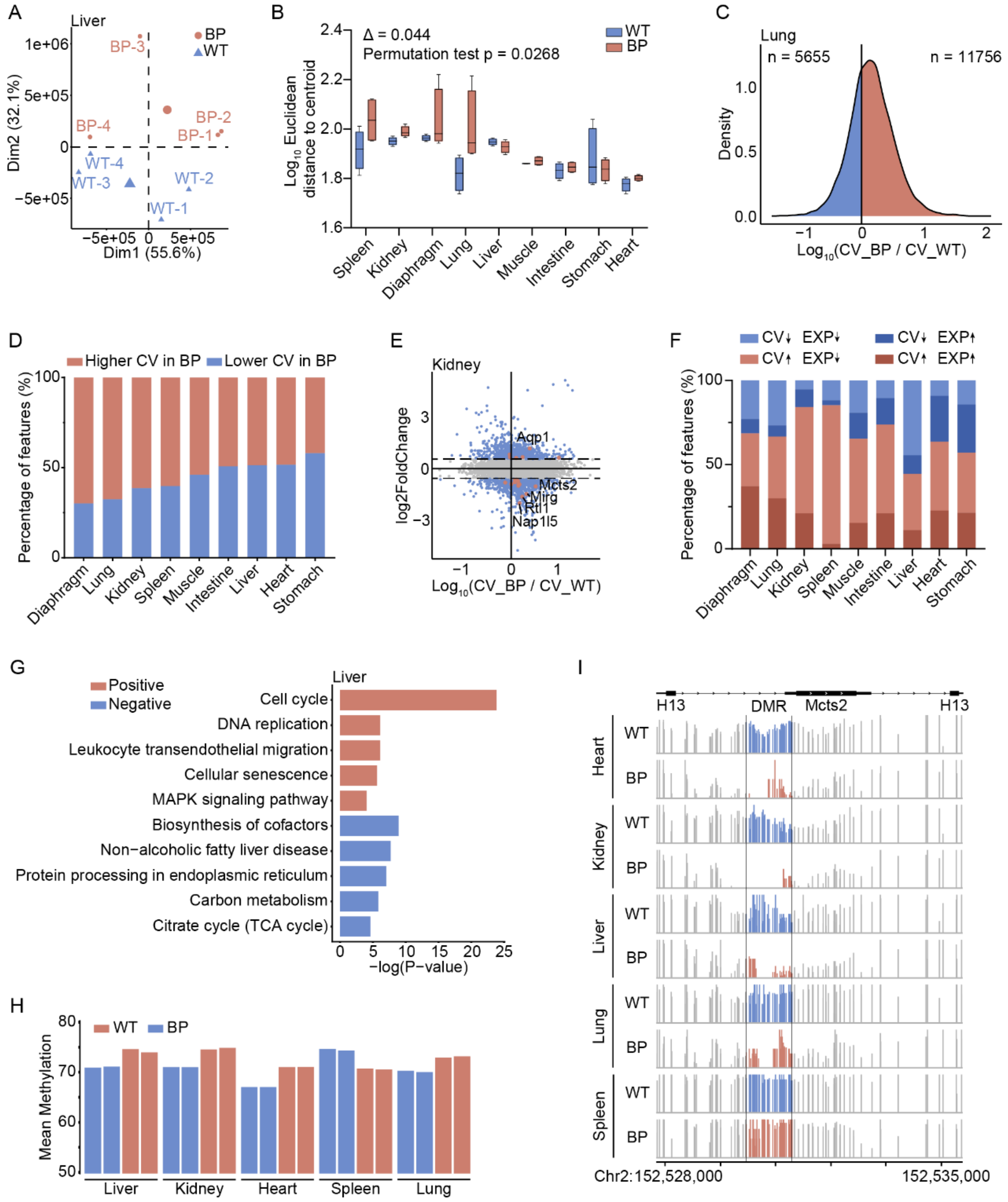

Revised Fig. 3A–I. Expanded multi-organ transcriptomic analysis under increased biological replication.

To address the reviewer's concern that the original organ-level RNA-seq and WGBS datasets were underpowered, we expanded the postnatal transcriptomic analysis from two to four biological replicates per group across nine organs, and we correspondingly revised the way these data are interpreted. Panel **A** provides a representative organ-level PCA, illustrating that BP samples can occupy a broader transcriptomic space than WT controls. Panel **B** summarizes this feature across nine organs by quantifying

distances to the group centroid in PCA space, showing increased within-group dispersion in BP tissues at the sample level. Panels **C–D** extend this analysis to the gene-expression level, showing that in multiple organs a substantial fraction of expressed genes exhibit broader expression ranges in BP relative to WT. Panels **E–F** further show that genes contributing to expression differences between genotypes frequently also display increased variability in BP tissues, indicating that broader dispersion is not limited to a small set of outlier loci. Panel **G** places these organ-level changes in relation to imprinted-gene-associated transcriptional programs, showing that imprinted loci remain involved but that the resulting expression changes occur within a broader tissue-level transcriptional landscape. Panels **H–I** provide revised methylation context for this section, showing modest global methylation differences across organs together with a representative locus-level example at the Mcts2-associated DMR. In the revised manuscript, these methylation data are presented conservatively as supportive epigenetic context rather than as the basis for strong organ-by-organ conclusions. Together, this revised figure shows that, under expanded biological replication, BP mice continue to exhibit broader organ-level transcriptional states across individuals after birth, while the methylation data provide complementary rather than primary support for this interpretation.

These analyses provide the expanded organ-level framework added in response to the reviewer's concern regarding limited replication and within-group variation in the original submission. In the revised manuscript, they are used to support a more restrained conclusion: across multiple postnatal organs, BP mice occupy broader transcriptional states across individuals than WT controls, whereas the methylation data are retained as supportive rather than primary evidence. These revisions are incorporated into the revised Results, the corresponding figure legend, and the broader revised manuscript.

4. The conclusion that the observed phenotype of the BP mice demonstrates functional imbalance of parental genomes beyond imprinting is a difficult one to make.

Although the authors have done a remarkable job in targeting 13 ICRs and deleting 6 other imprinted genes, the possibility remains that the effects reside in imprinted genes that remain unmanipulated. In this context, the imprinted locus Gpr1/Zdbf2 is relevant. This locus has not been manipulated in the BP mice. Maternal germline methylation of the Gpr1 ICR, which is only transiently maintained in the early embryo, is required for long-term imprinted expression of Zdbf2 in the hypothalamus. Lack of expression or altered imprinting of Zdbf2 results in significant postnatal growth and metabolic phenotypes (PMID: 27841881; PMID: 35049495). I am not suggesting that a failure to control Zdfb2 imprinting in BP mice is the cause of the neonatal liver metabolic phenotype observed in this study, but it is does indicate that it is very difficult to rule out the contribution of untested/unmanipulated imprinted genes to the effects.
Likewise, the Nap1l5 locus is unmanipulated in BP mice and, as the authors show in the RNA-seq analysis (Fig. 1C), is strongly up-regulated in at least two neonatal tissues. Although there has been no functional work on Nap1l5 imprinting, so that the consequences of its deregulated imprinting are unknown, it also makes the point that there are imprinted genes that are missed in the current BP model, making it very difficult to conclude that the BP phenotype is fully independent of imprinted gene deregulation.
The same applies to the Mcts2/H13 imprinted locus which does not appear to have been specifically manipulated - and which the authors highlight in network analysis and implicate in causing secondary effects on wider transcriptional programmes.
Statements in the Results and Discussion that "The resulting BP mice carried complete imprinting correction" is therefore limited to the imprinted loci that are specifically manipulated in the current study. And further, as the authors acknowledge from their transcriptomic analysis, "Analysis of imprinted genes revealed persistent dysregulation across all non-neural organs of BP mice".

Response:

We agree with the reviewer that the original conclusion was too strong. The current BP model does not allow us to conclude that the observed phenotypes are fully independent

of imprinted gene dysregulation, nor does it justify describing the animals as having complete imprinting correction except in the limited sense of the loci that were directly targeted. This applies not only to loci such as Gpr1/Zdbf2, Nap1l5, and Mcts2/H13 raised here, but more generally to the system itself: the currently defined set of imprinted loci may not yet be complete; parent-of-origin regulation can be tissue-dependent and context-dependent; and deletion-based engineering is not equivalent to restoring endogenous imprinting in its normal epigenetic form.

This point is also consistent with work currently underway in our group. In parallel with the present study, we have been examining parental methylation asymmetries in bi-maternal and bi-paternal mice to ask whether known imprinted loci can be recovered as expected and whether current approaches may still miss additional parent-of-origin-regulated loci. The logic of this effort follows the principle established in the reviewer's 2003 *Genome Research* study, namely that parental methylation differences can serve as an entry point for identifying previously unrecognized imprinted loci. Although this work is still in progress and is not used here as evidence for the present manuscript, it reinforces the reviewer's broader point that imprinting discovery itself may not yet be complete. For that reason, we cannot logically claim that all relevant imprinting effects have been corrected in the current BP model.

Accordingly, we did not attempt to defend the stronger framing of the original submission. Instead, we revised the manuscript throughout to remove or qualify statements that exceeded the evidential limits of the current system. In particular, we no longer use the study to argue that the observed phenotypes demonstrate parental genome imbalance cleanly beyond imprinting, and we no longer use "complete imprinting correction" in an unrestricted sense. The revised manuscript now takes a more limited position: after extensive correction of major known imprinting barriers, persistent abnormalities remain, but the present model does not allow these effects to be fully separated from residual dysregulation at known or as-yet-uncharacterized imprinted loci.

5. The observation "Unexpectedly, imprinting defects were accompanied by

widespread dysregulation of

non-imprinted autosomal genes…. Across multiple non-neural tissues, the number of differentially expressed non-imprinted genes exceeded that of imprinted genes, and in several organs the fold changes were even greater".

This seems to be a rather anecdotal statement.

Given that persistent dysregulation of some imprinted genes is observed, this would be expected to lead to a variety of down-stream transcriptional changes in tissues tested. This possibility is acknowledged in the subsequent paragraph, in which the authors suggest that disrupted imprinting (of Mcts2 and/or H13) may exert secondary effects on the wider transcriptional programme.

In addition, given the very limited number of replicates per tissue (2), it is difficult to robustly call differentially expressed genes, especially in comparing samples from separate litters with the confounds noted above.

Response:

We agree with the reviewer that this part of the original presentation was not adequately supported. With only two biological replicates per tissue, and with the limitations of the original comparison framework noted above, the emphasis on non-imprinted dysregulation as an "unexpected" finding and the contrast based on relative counts or fold changes were overstated.

In response, we made two corresponding changes in the revised manuscript. First, as described above, we expanded the organ-level RNA-seq analysis from two to four biological replicates per tissue. Second, we revised the interpretation accordingly: the organ transcriptomic data are no longer presented as evidence that non-imprinted dysregulation constitutes a separate or unexpected layer beyond imprinting. Instead, persistent dysregulation of imprinted loci is now treated more cautiously as one plausible contributor to broader transcriptional perturbation across tissues, involving both imprinted and non-imprinted genes.

These points are now addressed more clearly in the expanded organ-level analyses described above and in the revised manuscript, where this part of the Results has been

substantially reframed.

6. "Integration of expression and methylation data revealed a consistent relationship between transcriptional repression and promoter hypermethylation" - this statement relates to Fig. 2D and Fig. S3C. It is very unclear what these figures actually present: what does 'Density' on the y-axis represent; what does DE Value on the x-axis represent; and where is a methylation value plotted?
Further: "DNA methylation profiling further revealed hypermethylation at the promoters of these enzyme-encoding genes (Fig. S4C)". What does the scale on this heatmap represent? Ideally, the authors need to plot actual percentage methylation of these promoters so that an actual effect size is evident, as well as undertaking a statistical test, although with just 2 replicates this may not be possible.

Ultimately, the contention that the metabolic phenotype can be attributed to parental genome non-equivalence beyond imprinted genes seems premature. It is difficult to conclude whether the effects described represent consequences of dysregulated expression of imprinted genes not targeted in the 20KO model; because of some drift in expression of some targeted imprinted genes; or even epigenetic defects at non-imprinted loci sustained in ESCs during culture/manipulation.

Response:

We agree that the original presentation and interpretation of these methylation data were not sufficiently clear. In particular, the expression–methylation integration shown in the original manuscript was difficult to interpret from the figures themselves, and the associated wording was stronger than the available evidence could support. We therefore did not retain this part of the analysis in its original form in the revised manuscript.
Instead, in the revised version we have substantially narrowed the scope of what is concluded from the methylation data. Rather than using these analyses to argue for a consistent promoter-level relationship between transcriptional repression and

hypermethylation, or to support a broader claim that the metabolic phenotype reflects parental genome non-equivalence beyond imprinting, we now present the methylation results more conservatively as epigenetic context. In particular, we retained a representative locus-level example at the **Mcts2-associated DMR**, which provides a more direct and interpretable view of imprinting-associated methylation abnormality in BP mice across organs.

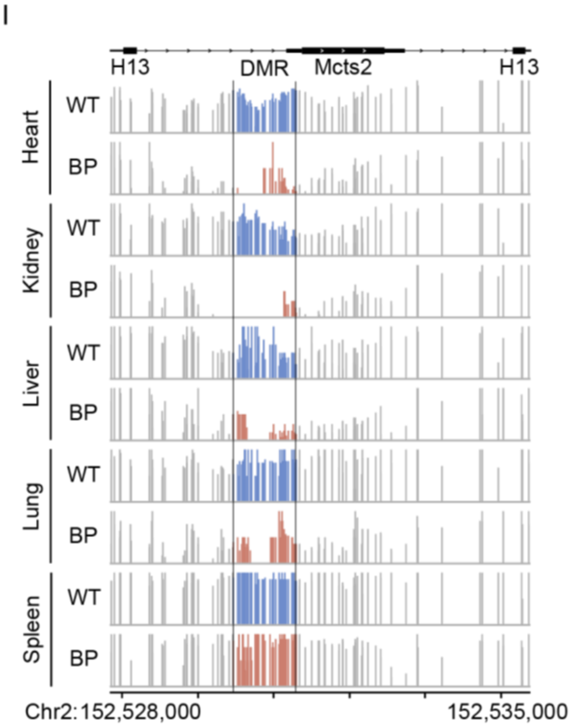

Fig. 3I. Representative DNA methylation profiles at the Mcts2-associated DMR across five organs at term.

Genome browser tracks show locus-specific methylation at the Mcts2-associated differentially methylated region in WT and BP mice, providing a representative example of imprinting-associated methylation abnormality in the revised dataset. This panel is included as a locus-level illustration and is not intended to support a global statement about promoter methylation or a direct causal explanation for the metabolic phenotype.

In the revised manuscript, this locus-specific display is used in a limited way: to provide

a clearer example of imprinting-associated methylation abnormality than the original summary-style presentation allowed. The metabolic findings are now interpreted more cautiously and are no longer framed as evidence that the observed liver phenotype can be cleanly attributed to parental genome imbalance beyond imprinting. Rather, the revised manuscript presents the adult liver metabolic state as a later physiological outcome that occurs in the setting of broader transcriptional and epigenetic abnormalities, while leaving open the possibility that residual dysregulation at known or uncharacterized imprinted loci, as well as features of the experimental system itself, may still contribute. These revisions are incorporated into the revised Results, figure legends, and the broader revised manuscript.

Notes on Figures:

Fig. 3C: please indicate in legend why 5 of the metabolites are identified by red text.

Response:

We thank the reviewer for this comment. In the original figure, the five metabolites were highlighted in red because they are related to glycolysis and the TCA cycle, which were examined in more detail in the subsequent analyses. We agree, however, that this selective labeling was unnecessary in a summary heatmap that includes a broader set of representative metabolites. In the revised manuscript, we therefore removed the red text and instead indicate the direction of change using upward and downward arrows. This presentation is also consistent with the later schematic and gene-expression panels, where the direction of change is shown in the same way. The corresponding figure and legend have been revised accordingly.

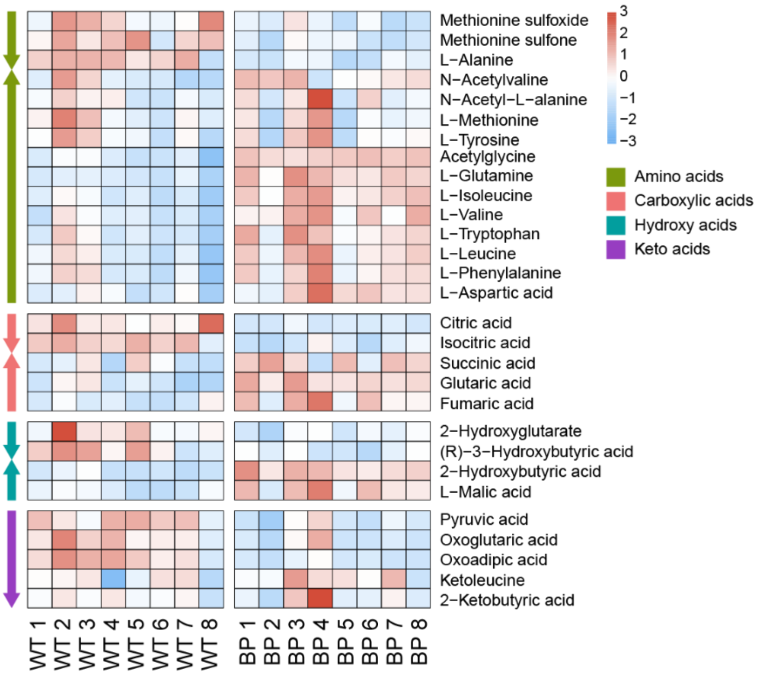

Revised legend text:

B Heatmap illustrating differential metabolite profiles between WT and BP samples. Metabolite abundances were scaled and visualized across individual biological replicates, with colors representing relative levels. Upward and downward arrows indicate metabolites increased or decreased in BP relative to WT, respectively. Metabolites are grouped by biochemical class, and samples are arranged by genotype.

Fig. 4B: there appear to be 5 datapoints for the WTs and BPs in these bar charts. Please specify in the legends what each datapoint represents: does each point represent a single pup, in which case is this from multiple areas of view/sections?

Response:

We thank the reviewer for this helpful comment. In this analysis, each dot does not represent an independent animal. Rather, the five datapoints were obtained from randomly selected microscopic fields sampled from multiple section levels of the same liver, with non-overlapping positions chosen to provide broader coverage of the tissue. These measurements were used to quantify the area and number of lipid vacuole–like structures across the liver sections. They are shown to illustrate the histological distribution of the phenotype within the liver tissue and are not intended to represent

independent biological replicates. We agree that this was not sufficiently clear in the original legend. In the revised manuscript, this panel has been renumbered as Fig. 4F, and the legend has been revised accordingly.

Revised legend text:

F Representative hematoxylin and eosin (H&E)-stained liver sections from WT and BP mice at term. Boxed regions are shown at higher magnification to highlight lipid vacuole–like structures (arrows). Quantification of lipid vacuole area (top right) and number (bottom right) is shown. Each dot represents one randomly selected, non-overlapping microscopic field sampled from different section levels of the same liver, chosen to provide broader coverage of the tissue.

Fig. 4C: are these data from pups or adults? Also, the range in muscle content (70-85%) appears far greater and much higher than expected, especially given the very limited variation in fat content (~7% in WT, ~22% in BP). Indeed, combining the fat and muscle content of at least one of the BP mice would lead to a total body composition of >100%, so it seems there is something wrong with part of this figure.

Response:

We thank the reviewer for carefully examining these data and for raising this important concern. These measurements were obtained from adult mice at 16 weeks of age, not from pups, and this has now been clarified in the revised manuscript. We also agree that the previous presentation was confusing. The body composition analyzer reports multiple compartments, including fat, muscle, and free water, and the fat and muscle values should therefore not be interpreted as complementary percentages that sum to 100% on their own. In addition, we found that part of the original dataset had been inadvertently omitted from the previous version of the figure, which contributed to the apparent inconsistency noted by the reviewer. We have now corrected the figure by including the complete dataset and have revised the corresponding legend and text accordingly.

Revised legend text:

Body composition analysis of adult WT and BP mice at 16 weeks of age, showing fat mass and muscle mass as measured by the body composition analyzer. These parameters are presented separately and do not sum to total body composition, which also includes additional compartments such as free water.

Availability of data: all RNA-seq, WGBS, and metabolomic datasets need to be deposited. There is also no information to ascertain the quality of these datasets in relation to read depth, numbers of genes observed, genomic coverage for methylation analysis.

Response:

We thank the reviewer for this important comment regarding data availability and dataset quality. All RNA-seq, whole-genome bisulfite sequencing (WGBS), and metabolomics datasets generated in this study have now been deposited in the appropriate public repository, and the corresponding accession information has been added to the Data Availability section of the revised manuscript.

In addition, to facilitate assessment of dataset quality, we have now included summary quality-control metrics in the revised manuscript and Supplementary Materials. Specifically, the newly added Supplementary Tables summarize key sequencing statistics, including total reads, mapped reads, and mapping rate for the RNA-seq datasets, as well as sequencing depth, mapping efficiency, and CpG coverage for the WGBS datasets. We appreciate the reviewer's careful attention to this point, which helped us improve the transparency of the revised manuscript.

Overall, we are grateful for the reviewer's careful and rigorous evaluation of this work. The comments raised here led us to strengthen the study substantially, both experimentally and interpretively, and helped us bring the manuscript to a clearer and more rigorously supported form.
