# Supplementary material for: Engineered bipaternal mice reveal the consequences of life without a maternal genomic contribution
Source: PLoS Biol. 2026 Jun 25;24(6):e3003871. doi: 10.1371/journal.pbio.3003871 (PMC13298975; doi:10.1371/journal.pbio.3003871)
Supplement: Attachment — Submitted filename: Response Letter.docx [file pbio.3003871.s019.docx]

**Point-to-point response**

We sincerely thank the Academic Editor and the reviewers for their careful evaluation of our revised manuscript and for their constructive comments. We are encouraged that the revised framing and added analyses were viewed as improving the manuscript, and we also appreciate the remaining concerns regarding methodological transparency, control-group interpretation, methylation analysis, and the strength of claims about variability. In this revision, we have further clarified the origins and genetic backgrounds of the WT embryos, WT ESCs, and BP ESCs; added details on embryo transfer, litter and recipient sources, sample numbers, sequencing batches, and analysis criteria; revised the nomenclature across the manuscript and figures; expanded the methylation analysis to a feature-resolved WGBS framework; and moderated statements where the experimental design supports a structured pattern of developmental and transcriptional divergence rather than strong standalone conclusions about variance. We address each comment in detail below.

REVIEWERS' COMMENTS:
Reviewer #1:
[identifies himself as Robert Feil]

The authors went through considerable efforts to address the points raised by this and the other reviewers. The manuscript now includes informative data on E12.5 embryos and, where needed, the authors added further replicates and technical control experiments.  In addition, they improved the presentation of their data and turned down the conclusions about non-imprinted genes.  they now also provide a balanced discussion that, importantly, highlights the limitations of the experimental systems used.   Overall, this is an excellent revision, which considerably improved the value of this manuscript.
**Response:**

We sincerely thank Dr Feil for his careful re-evaluation of our revised manuscript and for his encouraging assessment. The comments raised in the previous round were important in helping us recognize several limitations in the original presentation, particularly the need to define the scope of the engineered bipaternal system more clearly, to avoid over-interpreting transcriptional changes in non-imprinted genes, and to discuss the limitations of the experimental design more explicitly. In response, we added the E12.5 embryo analyses, incorporated additional replicates and technical controls where appropriate, revised the presentation of the data, and moderated the conclusions so that they more closely reflect the strength and limitations of the evidence. We are grateful that these revisions are now viewed as having improved the clarity and value of the manuscript, and we thank the reviewer again for the constructive guidance throughout the review process.

Reviewer #2:

The authors have done an excellent job substantially revising the paper. Most importantly, they moderated their claims of parental asymmetry beyond imprinting, conducted additional analyses for rigor, used more appropriate controls for the bipaternal offspring and clarified methods.

I do have one further request with respect to analysis: Figure 3H shows global DNA methylation levels across 5 organs in term WT and BP mice. Even though there are not many samples in each group, there could be greater analysis performed to try and understand where there the BP hypermethylation arises—the sequences can be separated by repetitive elements, regulatory elements, exons etc. These are standard analyses, which could be informative and useful.

**Response:**

We thank the reviewer for this encouraging assessment of the revised manuscript and for this helpful suggestion. We agree that the previous global methylation summary did not sufficiently indicate which genomic contexts contributed to the BP-associated methylation changes. We therefore expanded this analysis and replaced the previous global-only display with a feature-resolved WGBS panel in the main text.

Specifically, we stratified BP–WT methylation differences across major genomic annotations, including coding and exonic regions, repetitive elements, UTR/TSS-associated regulatory regions, and imprinted control regions/germline DMRs. We also added a matched hyper-DMR enrichment analysis to evaluate whether BP-hypermethylated regions were preferentially associated with specific sequence contexts. This analysis is now presented in revised Fig. 4G, and the corresponding sample-level whole-genome methylation overview has been moved to Fig. S6A, with the same tissue order as in the main figure.

The revised Results text now reads:

“Finally, because genomic imprinting is closely associated with DNA methylation, we analyzed mouse tissue WGBS data from WT and BP mice to examine BP-associated methylation patterns across multiple organs. To resolve the sequence contexts underlying the BP-associated methylation changes, we stratified BP–WT differences across major genomic annotations, including coding and exonic regions, repetitive elements, UTR/TSS-associated regulatory regions, and imprinted control regions/germline DMRs (Fig. 4G). These annotations are partially overlapping and provide sequence-context information rather than an exhaustive non-overlapping partition of the genome. This analysis revealed that methylation differences were organized by both genomic context and tissue type. Several genic and regulatory annotations, including 5′ UTR, TSS-associated regions and ICRs/gDMRs, showed prominent BP-associated hypermethylation or hyper-DMR enrichment, whereas other repeat and intergenic classes showed more variable or tissue-dependent patterns. Sample-level global mCG values are provided as a whole-genome overview in Fig. S6A, and locus-level methylation and coverage at the Mcts2/H13 gDMR are shown in Fig. 4H. Together, these results provide a more resolved epigenetic framework for interpreting the postnatal transcriptional abnormalities observed in BP tissues.”

We have also revised the figure legend accordingly:

Fig. 4G. Element-level DNA methylation differences and BP-hypermethylated DMR enrichment across five mouse tissues. The heatmap shows BP minus WT ΔmCG across genomic features including coding regions, exons, transposable element subclasses, TSS, UTRs, and ICRs/gDMRs. The bar plot shows enrichment of merged BP-hypermethylated DMRs in each genomic feature. Positive ΔmCG values indicate higher methylation in BP. Related sample-level whole-genome methylation values are shown in Fig. S6A.

Fig. S6A. Global mCG levels across five mouse tissues in WT and BP samples. Each dot represents one WGBS sample.

We are grateful to the reviewer for these constructive and thoughtful suggestions. The reviewer’s comments helped us refine several important aspects of the manuscript, including moderating the interpretation of parental asymmetry beyond imprinting, strengthening the analysis of non-imprinted gene dysregulation, clarifying the methodological basis of the experimental system, and improving the presentation of methylation changes across tissues. In response, we revised the wording of the main conclusions, added more rigorous analyses and controls, clarified the limitations of the system, and replaced the previous global methylation summary with a feature-resolved analysis that identifies the genomic contexts associated with BP methylation changes. These revisions have substantially improved both the precision and the balance of the manuscript, and we sincerely thank the reviewer for the careful reading and constructive guidance.

Reviewer #3:
[identifies himself as Gavin Kelsey]

This manuscript represents a substantial reframing of the previous submission "Functional imbalance in bi-paternal mice reveals postnatal consequences of parental genome asymmetry".  The authors have pivoted away from their earlier interpretation that the physiological phenotype of 'bipaternal' mice was evidence for a contribution of the paternal genome beyond classical genomic imprinting.  This change in emphasis is welcome.  Instead, the authors frame the manuscript in terms of developmental and transcriptional variation, with the interpretation that the presence of both parental genome contributions provides developmental robustness, thereby helping to "constrain mammalian development within a relatively narrow range of trajectories".  This reframing is bolstered by the addition of new samples in the postnatal tissue transcriptome analysis (increasing sample size from 2 to 4), as well as addition of transcriptome analysis of mid-gestation embryos.  The latter, importantly, incorporates a new and more appropriate control group.  The author's new interpretation has interesting parallels with observations that although parthenogenesis is observed in some non-mammalian vertebrates, success of uniparental reproduction is generally limited with variable outcomes.

As before, the study is characterised by the authors' experimental skills in elegant embryo manipulations, and achieving a live birth rate of 15% of the engineering biparental mice is a fantastic achievement.  But there remain concerns about just how much the authors can conclude about phenotypic variability with their current experimental design.

I suggest that the authors should address the following points.

**Response:**

We thank Dr Kelsey for his careful and constructive re-evaluation of our revised manuscript. We appreciate the reviewer’s recognition that the manuscript has been substantially reframed and that the added E12.5 embryo analyses, expanded postnatal transcriptome sampling, and inclusion of a WT ESC-derived tetraploid-complementation control have strengthened the study.

We also agree that the current experimental design requires careful definition of the control framework, transparent reporting of methodological details, and appropriately bounded interpretation of phenotypic and transcriptional variability. In response to these comments, we have further revised the manuscript to clarify the genetic background and identity of the WT, WT ESC, and BP ESC materials; to provide additional details on embryo transfer, litter origin, sample numbers, sequencing batches, and analytical inclusion criteria; to use consistent nomenclature for WT TC and BP TC samples throughout the text and figures; and to moderate statements where the data support a pattern of developmental and transcriptional divergence but not a strong quantitative claim about variance.

Importantly, we have also revised the interpretation of the WT TC control. Rather than treating naturally conceived WT embryos and WT TC embryos as interchangeable, the revised text now explicitly presents WT TC embryos as defining an ESC-derived tetraploid-complementation baseline, with BP TC embryos showing additional or BP-associated deviations within this background. We believe these revisions address the reviewer’s main concerns and improve the precision, transparency, and balance of the manuscript. We address each point in detail below.

1. Some methodological details are still missing:
The main control group - the naturally conceived embryos and offspring - are referred to as WT.  The authors should identify in the Methods which mouse strain these are and whether they are isogenic with the ES cells used (both the engineered BP-ESCs and WT-ESCs).  The authors should also confirm the genetic background of the BP-ESCs and WT-ESCs.  Please also specify what the WT-ESCs are.  My assumption is that they are unmanipulated, diploid biparental ESCs, but please confirm (you could conceive of an experimental design in which the ideal control ESCs were diploid androgenetic, but these would not support development to the necessary stage).

**Response:**

We thank Dr Kelsey for identifying these missing methodological details. We agree that the genetic background and origin of the WT embryos, WT ESCs, and BP ESCs should be stated explicitly to allow readers to interpret the control groups appropriately. We have therefore clarified these details in the Methods under the subsection “Mouse strains and ESC lines.”

Specifically, the naturally fertilized WT embryos and offspring used as controls were obtained from in vivo fertilization using C57BL/6J mice. The WT ESC line used for tetraploid complementation was an unmanipulated diploid biparental ESC line derived from naturally fertilized C57BL/6J-GFP embryos cultured to the blastocyst stage. The BP ESC line used for tetraploid complementation was the 20KO androgenetic diploid ESC line established as described in the following Methods section and in our previous study. All WT embryos, WT ESCs, BP ESCs, and sperm donors used for these experiments were on a C57BL/6J genetic background unless otherwise stated.

We also confirm that the WT ESCs used here were conventional diploid biparental ESCs derived from fertilized embryos, not diploid androgenetic ESCs. We have added this clarification to the Methods to avoid ambiguity about the nature of the WT TC control.

2. Concern about the control groups:
The authors have responded to my comments and those of reviewer 1 by adding tetraploid complementation with WT ES cells as a control group, which they apply in a new section on embryonic outcomes (analysed at e12.5).  This is an important improvement of the new manuscript.  But it also sets up an inconsistency in the manuscript, in that WT ES constructs are seen as the ideal control in the embryonic analysis, whereas naturally conceived WT animals provide the only control group in all the postnatal analyses.  The Sankey plot in Fig. S1C is important in this respect.  The impression from this plot is that there is much greater concordance of gene expression states between the BP TC and ES (WT) TC groups than between either of the TC groups and the WT group.  This seems logical, as the comparison is between unmanipulated, naturally conceived embryos (WT) and the two classes of ESC-derived embryos with tetraploid complementation.  But it means that the control group for the
postnatal analysis is not optimal.

**Response:**

We thank Dr Kelsey for this important point. We agree that naturally conceived WT animals and WT ESC-derived tetraploid-complemented embryos address different control questions, and that the postnatal analyses should be interpreted with this distinction in mind. In the revised manuscript, we have therefore clarified that naturally conceived WT embryos and animals serve as the reference for normal biparental development, whereas WT TC embryos provide a procedure-matched embryonic baseline for effects associated with ESC derivation and tetraploid complementation.

We also agree that the partial concordance between WT TC and BP TC embryos in the Sankey analysis is informative. To examine this relationship more explicitly, we added a new integrated analysis in Fig. 3A. Genes were first classified from the E12.5 RNA-seq data into stable, WT TC-shifted, BP-amplified, and BP-specific classes according to their expression patterns across WT, WT TC, and BP TC embryos. This classification separates changes shared by WT TC and BP TC embryos from changes that were amplified or more specific in BP TC embryos.

The WT TC-shifted class captures genes concordantly shifted in WT TC and BP TC embryos relative to naturally conceived WT embryos. This class was small but strongly enriched for imprinted genes and showed methylation differences in WT ESCs relative to ICM, consistent with the sensitivity of these loci to the ESC-derived experimental context. By contrast, the BP-amplified and BP-specific classes identify gene groups in which BP TC shows additional deviation within or beyond this baseline. These categories therefore provide a more resolved framework for interpreting the E12.5 data than a simple WT versus WT TC versus BP TC comparison.

We then asked whether these E12.5 RNA-seq-defined gene classes showed corresponding methylation features in ESC WGBS and mouse WGBS datasets. For the ESC comparison, we used public WT ESC and androgenetic ESC WGBS datasets relative to ICM, including WT ESC ES-f3 (GSM4603224, passage 8) and aESC samples aES2 and aES3 (GSM4603222 and GSM4603221, both passage 8). The ICM methylation dataset was GSM1386023 (ICM_MethylC-Seq; GSE56697), originally provided in mm9 coordinates and lifted to mm10 before comparison. For the mouse WGBS comparison, BP minus WT methylation differences were summarized across the five available tissue WGBS datasets.

We do not interpret this analysis as direct lineage tracing or as evidence for causal inheritance of methylation states from ESCs to embryos and then to mouse tissues. Rather, it provides cross-stage concordance between the starting-cell methylome, the E12.5 embryo transcriptome, and the mouse tissue methylome endpoint. This addition helps us acknowledge the limitation raised by the reviewer while also distinguishing the shared ESC-derived component from BP-amplified and BP-specific components. We have incorporated this framework into the Results, figure legend, and Methods, and have moderated the postnatal interpretation accordingly.

The revised Results text now reads:

“The observation that some stability-state transitions were shared by WT TC and BP TC embryos indicated that the E12.5 transcriptomic differences included both a procedure-associated component and BP-associated components. To separate these patterns more explicitly and to link the embryonic transcriptome to methylome features at other levels of the system, we classified E12.5 expressed genes into stable, WT TC-shifted, BP-amplified, and BP-specific groups according to their expression patterns across WT, WT TC, and BP TC embryos (Fig. 3A). The WT TC-shifted class captured genes concordantly shifted in WT TC and BP TC embryos relative to WT; this class was small but strongly enriched for imprinted genes and showed methylation differences in WT ESCs relative to ICM, consistent with the sensitivity of these loci to the ESC-derived experimental context. The BP-amplified and BP-specific classes identified genes with additional BP-associated deviation, and these classes showed corresponding methylation features in aESC/ICM and BP-WT mouse WGBS comparisons. Although these comparisons do not establish direct methylation inheritance across stages, they provide cross-stage methylome context for the E12.5 RNA-seq-defined gene classes. This framework also provided a basis for examining how BP-associated transcriptional and methylation states are represented across mouse tissues.”

Figure 3A Integrated summary of E12.5 RNA-seq-defined gene classes and corresponding WGBS methylation features. Genes were classified as stable, WT TC-shifted, BP-amplified, or BP-specific according to expression patterns across WT, WT TC, and BP TC embryos. In the category schematics, gray, blue, and red indicate WT, WT TC, and BP TC, respectively. The RNA-seq summary shows gene number, imprinted gene enrichment, and mean absolute log2 fold-change relative to WT. Dot-strip plots show ΔmCG for gene-associated regions in WT ESC versus ICM, aESC versus ICM, and BP versus WT mouse WGBS comparisons. Each dot represents one gene-associated region. Solid vertical lines indicate median ΔmCG; dashed lines indicate ΔmCG = 0.

3. Tendency to overstatement of results:
There are instances where results are rather cursorily or selectively described; for example:
"Consistent with this pattern, pairwise Euclidean distance analysis revealed significantly greater within-group distances among BP TC embryos than among WT embryos, whereas WT TC embryos showed no comparable increase relative to WT embryos (Fig. 2E)."  But it is also the case in this figure that there is no significant difference in the Euclidean distance between the WT TC and BP TC groups, which is arguably the more relevant comparison.

"Differential expression analysis followed by gene set enrichment analysis identified alterations in pathways related to core cellular and metabolic processes, including protein translation, complement activation, and glucose metabolism (Fig. 2F). Notably, pathway-level module scores also displayed broader distributions among BP embryos than among WT embryos, indicating that transcriptional states associated with these biological processes varied across individuals."  But it is not clear that BP TC embryos show a greater range of values than the WT TC (labelled ES TC in the figure).

"WT TC embryos showed only a modest rightward shift relative to WT embryos, suggesting that tetraploid complementation introduces limited transcriptional variability, whereas the BP condition further increases this range (Fig. 2G)."  Whereas there seems to be a major shift between WT and ES TC and a more modest difference between the ES TC and BP TC groups.

"Principal component analysis (PCA) showed that WT samples generally formed relatively compact clusters within each tissue, whereas BP samples occupied wider regions of transcriptional space in several organs (Fig. 3A, S3A)."  It is not evident that there is greater dispersion of BP TC samples in most of the PCA plots in Fig. S3A.

Finally in this context, although the sample number for the postnatal analysis has been increased to 4, which is welcome, if the new emphasis of the study is transcriptional or phenotypic variation, it is not clear that this sample size is sufficient to make robust conclusions of variance.
**Response:**

We thank Dr Kelsey for pointing out these instances. We agree that several statements in the previous version described the data too broadly and did not sufficiently distinguish BP-associated effects from changes shared by WT TC and BP TC embryos. We have therefore revised the Results to avoid implying that BP TC embryos are uniformly more variable than WT TC embryos across all metrics, or that WT TC embryos are equivalent to naturally conceived WT embryos. The revised interpretation now separates a shared ESC-derived tetraploid-complementation baseline from BP-associated additional or stronger deviations.

For the E12.5 embryo analyses, we added a new integrated perturbation map as revised Fig. 2E. Rather than relying on the Euclidean distance plot, selected pathway module plots, or CV distribution alone, this panel integrates sample-level perturbation scores with gene-level expression deviations across individual WT, WT TC, and BP TC embryos. The upper tracks summarize imprinted-gene perturbation, Euclidean distance to the WT centroid, and pathway-level perturbation for each embryo. The heatmap then shows WT-centered expression deviations for representative imprinted genes, aberrant non-imprinted genes grouped by translation, immune/complement, and metabolism-related modules, and stable non-imprinted genes.

This revised presentation directly addresses the reviewer’s concerns about the previous Fig. 2E–G. First, for the Euclidean distance analysis, we agree that the lack of a significant WT TC–BP TC difference is important and should not be overlooked. The revised text no longer interprets this analysis as evidence that BP TC embryos are clearly separated from WT TC embryos by this global metric. Instead, Fig. 2E shows that WT TC embryos already carry measurable transcriptional perturbation relative to naturally conceived WT embryos, and that BP TC embryos should be interpreted within this shared ESC-derived TC background. Second, for the pathway module scores, we no longer use a few representative pathway plots as standalone evidence that BP TC embryos have a broader range than WT TC embryos. These pathway analyses are now incorporated into a sample-resolved perturbation framework. Third, for the CV distribution, we have revised the interpretation to acknowledge that WT TC embryos show a clear shift relative to WT embryos, consistent with the ESC-derived tetraploid-complementation procedure contributing to the transcriptional baseline. BP TC embryos are therefore described as showing additional or stronger deviations within this already shifted background, rather than as a completely separate state in every analysis.

The revised Results text now reads:

“To further examine how transcriptional perturbation was distributed across individual embryos, we integrated sample-level perturbation scores with expression deviations of selected gene classes (Fig. 2E). WT TC embryos already showed measurable deviation from naturally conceived WT embryos, particularly in imprinted-gene and pathway-level perturbation scores, indicating that ESC-derived tetraploid complementation establishes a detectable transcriptional baseline. BP TC embryos shared this baseline but showed additional or stronger deviations across imprinted genes and representative non-imprinted gene modules. In contrast, a set of stable non-imprinted genes remained largely unchanged across groups, indicating that the observed perturbation was structured rather than uniformly genome-wide. These results support the interpretation that BP TC embryos exhibit incomplete normalization within an ESC-derived developmental background.”

Fig. 2E. Integrated expression perturbation map across individual E12.5 embryos. Upper tracks show sample-level imprinted-gene, WT-centroid distance, and pathway perturbation scores for WT, WT TC, and BP TC embryos. The heatmap shows WT-centered expression deviations for selected imprinted genes, representative aberrant non-imprinted genes related to translation, immune/complement, and metabolism, and stable non-imprinted genes. Red and blue indicate increased and decreased expression relative to the WT mean, respectively.

We also revised the description of the postnatal PCA analyses. We agree that most individual PCA plots in Fig. S3A do not by themselves demonstrate greater BP dispersion. We have therefore avoided presenting the PCA panels as direct evidence that BP samples are more dispersed in most tissues. Instead, we now describe these data as showing tissue-dependent transcriptional organization, with the quantitative dispersion analysis interpreted as an across-organ summary rather than a claim supported by each individual PCA panel.

The revised text now reads:

“Principal component analysis (PCA) showed tissue-dependent separation between WT and BP samples, with the extent and direction of separation differing among organs (Fig. 4A, S3A). Quantification of distances to group centroids showed a tendency toward increased dispersion in BP samples in several organs, and an across-organ permutation analysis indicated a modest overall increase in BP within-group dispersion relative to WT (Fig. 4B). These analyses suggest altered transcriptional organization across BP tissues, although individual tissue-level dispersion patterns should be interpreted cautiously given the limited number of biological replicates.”

Finally, we agree that n = 4 per group is not sufficient to support strong standalone conclusions about variance in each postnatal tissue. We have therefore moderated the language throughout this section. The revised manuscript no longer frames the postnatal RNA-seq data as providing definitive proof of increased variance across tissues. Instead, we describe the results as dispersion-related patterns and gene-level variability trends that are consistent across several analyses. The technical replicate experiment is now presented only as evidence that the observed differences are unlikely to be explained primarily by library construction or sequencing noise, not as a substitute for larger biological sample sizes. We have also stated this limitation more explicitly in the Discussion.

4. Some experimental details are missing, which should be provided for clarity:

Fig. 1E: please specify how many litters these data derive from.

Fig. 2C: are the embryo length data from single or multiple litters per group?  The point here being that readers should know whether the greater variation in length in the BP TC group is not a result of a technical or trivial factor, such as the WT embryos being from a single litter but the BP TC embryos from 2 or more litters that could have slight developmental asynchrony.  It could be that the production of BP TC embryos is highly efficient, so that all embryos are from the same litter, but we need to know this.

Fig. 3I: what is the scale for the methylation plot?  If the highlighted region is indeed the gDMR for Mcts2/H13, the expectation is that in the WT tissues it should be ~50% methylated.

One of the problems with this sort of plot is that where there are 'gaps' - for example, across much of the gDMR in the BP kidney - it is not clear whether this is because the sites are unmethylated or because there are missing values.  Having a plot that also incorporates read number per position would eliminate that ambiguity.
**Response:**

We thank Dr Kelsey for these helpful requests for clarification. We have added the missing sample-origin information to the figure legends and Methods, and we have revised the Mcts2/H13 methylation plot to clarify both the methylation scale and read coverage across the highlighted interval.

For the placental area quantification in Fig. 1E, WT placentas were collected from two naturally pregnant females, whereas WT TC and BP TC placentas were collected from six and eight recipient females, respectively. Each data point represents one placenta. This information has now been added to the Fig. 1 legend and to the Methods.

For the embryo body-length quantification in Fig. 2C, six embryos were analyzed per group. WT embryos were naturally conceived embryos collected at E12.5 from two litters. WT TC embryos were obtained from four recipient females after embryo transfer, and BP TC embryos were obtained from three recipient females after embryo transfer. Thus, the data were not derived from a single litter per group. We agree that litter origin, embryo transfer, and recipient females can contribute to variation in developmental timing and embryo size. We have therefore added this information to the Methods and Fig. 2 legend, and we have moderated the interpretation so that the broader distribution of BP TC embryo length is not attributed solely to intrinsic BP-associated developmental variability.

For the methylation plot, previously shown as Fig. 3I and now revised as Fig. 4H, we agree that the original presentation did not provide sufficient information to distinguish low methylation from missing data. We have therefore revised this panel in three ways. First, we redefined the highlighted interval using the Mcts2/H13 gDMR interval chr2:152528194–152528847 in GRCm39, consistent with the public parental methylome annotation used for the ICM reference dataset. Second, we added an explicit methylation scale, with CpG methylation levels plotted from 0 to 100%. Third, we added coverage tracks showing read depth at the same genomic positions. These coverage tracks clarify where the absence of methylation bars reflects low or absent coverage rather than unmethylated CpGs.

The revised plot shows site-level CpG methylation and coverage across the Mcts2/H13 gDMR in five mouse tissues. As expected for an imprinted gDMR, WT samples show intermediate methylation across covered CpGs within the highlighted region, although the plotted values are not uniformly 50% at every CpG position. This likely reflects the site-resolved nature of the display, tissue composition, local CpG coverage, and averaging across the available biological replicates. We have therefore described the panel as a representative locus-level methylation profile rather than as a quantitative estimate of exactly 50% methylation across the entire gDMR.

The revised figure legends now include the following information:

**Fig. 1E.** Quantification of placental area at E12.5 for WT, WT TC, and BP TC placentas. WT placentas were collected from two naturally pregnant females, whereas WT TC and BP TC placentas were collected from six and eight recipient females, respectively. Each dot represents one placenta.

**Fig. 2C.** Quantification of embryonic body length at E12.5 for WT, WT TC, and BP TC embryos. Body-length quantification included six embryos per group. WT embryos were collected from two naturally conceived litters, whereas WT TC and BP TC embryos were obtained from four and three recipient females, respectively, after embryo transfer. Each dot represents one embryo.

**Fig. 4H.** DNA methylation profiles at the Mcts2/H13 gDMR across five mouse tissues. CpG methylation levels are shown for WT and BP samples in heart, liver, lung, kidney, and spleen. Vertical bars indicate CpG methylation level (%), and coverage tracks show sequencing depth at the same positions. The shaded interval marks the Mcts2/H13 gDMR.

Other corrections/comments

Consistency in labelling: the WT ESC tetraploid-complemented embryos are variously labelled as WT TC, ES TC and even ES 4N in the figures (e.g., Fig. 1, Fig. 2), and even differently within the same figure (Fig. S1).  Similarly, the BP TC samples sometimes are referred to as BP 4N.  Please use a consistent nomenclature for the samples.
**Response:**

We thank Dr Kelsey for pointing out this inconsistency. We agree that the mixed use of WT TC, ES TC, ES 4N, BP TC, and BP 4N could cause confusion. We have revised the manuscript, figure labels, figure legends, and Methods to use a consistent nomenclature throughout.

Specifically, naturally conceived wild-type embryos or animals are referred to as WT. Wild-type ESC-derived tetraploid-complemented embryos are referred to as WT TC. Bipaternal ESC-derived tetraploid-complemented embryos or animals are referred to as BP TC. We no longer use ES TC, ES 4N, or BP 4N in the revised figures or text. This nomenclature has been applied consistently across the main figures, supplementary figures, Results, Methods, and figure legends.

We appreciate the reviewer’s careful attention to this point. This correction has made the experimental groups easier to follow and has improved the clarity of the revised manuscript.

COMMENTS FROM THE ACADEMIC EDITOR [lightly edited]:

Overall, I agree that the manuscript is much improved as noted by the reviewers. I also agree with reviewer #3 that much more details on methodology needs to be provided for a true biological interpretation/understanding of the phenomenon. As a reader I need to know if the results are biologically relevant or a phenomenon that results form the extensive manipulations that these cells/embryos undergo.

One thing I could not find in the revision is information about the sequencing and whether the new samples were sequenced separate from the original samples, or if the original samples were re-sequenced with the added observations. If the former, this alone introduces a lot of variability in the results.

Also, one thing that is difficult for me to see as a biologist is the inconsistent number of observations for the groups between the figures. Also, when reading the methodology, which is incredibly important to fully understand prior to accepting findings as having biological relevance, there is an overall lack of detail as to how many of any were done, collected, analyzed etc. For example, how many embryos were transferred in order to get the few analyzed? Complete transparency of methodology is imperative for the reader to be able to determine the strength of the results.

I agree with Reviewers #1 and #2 that this manuscript should be added to the body of literature becasue it represents an amazing feat, but I also agree with reviewer #3 about potential bias in biological interpretation resulting from the methods. Overall, I think the paper is much improved but authors must carefully consider and answer all of the reviewer #3's concerns and the point brought up by reviewer #2 prior to publication.

**Response:**

We thank the Academic Editor for the careful assessment and for emphasizing the need for greater methodological transparency. We agree that, in this experimental system, readers need sufficient information to distinguish BP-associated biological patterns from effects that may arise from ESC derivation, embryo manipulation, tetraploid complementation, recipient females, litter origin, or sequencing batch structure. In response, we have revised the manuscript to provide additional experimental details in the Results, Methods, figure legends, and supplementary tables, and we have further moderated the interpretation where the current design does not allow complete separation of these factors.

First, we have clarified the control framework and interpretive limits of the study. As also noted in our response to Reviewer #3, naturally conceived WT embryos and animals provide the reference for normal biparental development, whereas WT TC embryos provide a procedure-matched embryonic baseline for ESC derivation and tetraploid complementation. We now interpret BP TC findings in relation to both of these contexts, rather than treating WT TC and naturally conceived WT as interchangeable controls. We have also revised statements about transcriptional and phenotypic variability to avoid implying that the current sample sizes support strong tissue-by-tissue variance conclusions.

Second, we have clarified the sequencing batch structure. All E12.5 whole-embryo RNA-seq samples, including WT, WT TC, and BP TC embryos, were prepared and sequenced in the same batch. For the nine-organ transcriptomic dataset from term samples, the original samples and additional biological replicates generated during revision were sequenced in two RNA-seq batches. Batch effects between the two RNA-seq batches were modeled using DESeq2 by incorporating batch as a covariate in the design formula. For exploratory analyses, including PCA, CV analysis, and correlation analysis, batch-adjusted expression values were generated before downstream visualization and comparison. These details have been added to the Methods, and sequencing quality and sample information are summarized in Supplementary Table S1. WGBS sequencing information, including sequencing depth, mapping efficiency, and CpG coverage, is provided in Supplementary Table S2.

Third, we have added more detailed information on the numbers of embryos generated, transferred, collected, and analyzed. The E12.5 WT TC and BP TC embryos analyzed in this study were obtained from multiple independent tetraploid-complementation experiments. WT TC embryos were collected from five independent experiments, and BP TC embryos were collected from five additional independent experiments. In each tetraploid-complementation experiment, 60 tetraploid embryos were transferred in total, with 20 embryos transferred into each CD-1 pseudopregnant recipient. For the term BP TC mice newly generated for nine-organ collection, three independent tetraploid-complementation experiments were performed, again with 60 tetraploid embryos transferred per experiment and 20 embryos transferred per recipient.

We have also clarified why the number of observations differs among figures. Different analyses were performed at different biological and technical levels: E12.5 whole-embryo RNA-seq analyses used six embryos per group; E12.5 body-length quantification used six embryos per group; term nine-organ RNA-seq analyses used four biological replicates per genotype per tissue; and mouse WGBS analyses used two biological replicates per genotype per organ. For the E12.5 body-length analysis, WT embryos were naturally conceived embryos collected from two litters, whereas WT TC and BP TC embryos were recovered from four and three recipient females, respectively. The embryos used for body-length measurement were photographed immediately after dissection and then collected for RNA-seq analysis, so the morphological and transcriptomic analyses were performed on overlapping embryos. We have revised the figure legends and Methods to state what each data point represents and to provide the relevant sample size for each analysis.

The revised Results text now includes the following clarification in the E12.5 embryo section:

“The E12.5 WT TC and BP TC embryos analyzed here were obtained from multiple independent tetraploid-complementation experiments. WT TC embryos were collected from five independent experiments, and BP TC embryos were collected from five additional independent experiments; in each experiment, 60 tetraploid embryos were transferred in total, with 20 embryos transferred into each CD-1 pseudopregnant recipient. Body length was quantified from six embryos per group. WT embryos were naturally conceived embryos collected from two litters, whereas WT TC and BP TC embryos were recovered from four and three recipient females, respectively.”

The revised Results text also includes the following clarification in the multi-organ tissue section:

“The term BP mice used for the additional nine-organ collection were generated from three independent tetraploid-complementation experiments, each involving transfer of 60 tetraploid embryos in total, with 20 embryos transferred into each CD-1 pseudopregnant recipient.”

Finally, we have addressed the specific point raised by Reviewer #2 by replacing the previous global-only methylation summary with a feature-resolved WGBS analysis, and we have addressed the concerns raised by Reviewer #3 by clarifying strain and ESC-line origins, sample sources, litter and recipient information, sequencing batch structure, nomenclature, and the interpretation of WT TC as an embryonic procedural baseline. We are grateful to the Academic Editor and the reviewers for their careful reading and constructive guidance throughout this revision. These comments have helped us improve the transparency of the experimental design, clarify the control framework, strengthen the methylation analyses, and moderate the interpretation of variability in this technically complex system. We believe the revised manuscript is now clearer, more balanced, and better supported by the data.
