# Supplementary material for: Engineered bipaternal mice reveal the consequences of life without a maternal genomic contribution
Source: PLoS Biol. 2026 Jun 25;24(6):e3003871. doi: 10.1371/journal.pbio.3003871 (PMC13298975; doi:10.1371/journal.pbio.3003871)
Supplement: Peer review history

# Author comment: Author response to Decision Letter 0

**DOI:** 10.1371/journal.pbio.3003871.r001

12 Mar 2026

Attachment Submitted filename: Point-to-point response.pdf

# Editor report: Decision Letter 0

**DOI:** 10.1371/journal.pbio.3003871.r002

**Authors:** Roland Roberts

31 Mar 2026

Dear Zhikun,

Thank you for submitting your revised manuscript entitled "Developmental and physiological features of bipaternal mice lacking maternal genomic contribution" for consideration as a Short Report by PLOS Biology.

Your manuscript has now been evaluated by the PLOS Biology editorial staff, and I'm writing to let you know that we would like to send your submission out for re-review.

IMPORTANT: I'd like to apologise for the delay incurred; we were discussing your revisions with the Academic Editor, but unfortunately a family emergency means that they were not able to provide their assessment in time. As a result, although we will send your revised paper back out to the reviewers, we are not fully convinced that they will be persuaded by the changes that you made.

However, before we can send your manuscript back to the reviewers, we need you to complete your submission by providing the metadata that is required for full assessment. To this end, please login to Editorial Manager where you will find the paper in the 'Submissions Needing Revisions' folder on your homepage. Please click 'Revise Submission' from the Action Links and complete all additional questions in the submission questionnaire.

Once your full submission is complete, your paper will undergo a series of checks in preparation for re-review. After your manuscript has passed the checks it will be sent out for review. To provide the metadata for your submission, please Login to Editorial Manager (https://www.editorialmanager.com/pbiology) within two working days, i.e. by Apr 02 2026 11:59PM.

During the process of completing your manuscript submission, you will be invited to opt-in to posting your pre-review manuscript as a bioRxiv preprint. Visit http://journals.plos.org/plosbiology/s/preprints for full details. If you consent to posting your current manuscript as a preprint, please upload a single Preprint PDF.

Feel free to email us at plosbiology@plos.org if you have any queries relating to your submission.

Kind regards,

Roli

Roland Roberts, PhD

Senior Editor

PLOS Biology

rroberts@plos.org

# Author comment: Author response to Decision Letter 1

**DOI:** 10.1371/journal.pbio.3003871.r003

1 Apr 2026

Attachment Submitted filename: Point-to-point_response_auresp_1.pdf

# Peer review: Decision Letter 1

**DOI:** 10.1371/journal.pbio.3003871.r004

**Authors:** Roland Roberts, Roland Roberts

6 May 2026

Dear Zhikun,

Thank you for your patience while we considered your revised manuscript "Developmental and physiological features of bipaternal mice lacking maternal genomic contribution" for consideration as a Short Report at PLOS Biology. Your revised study has now been evaluated by the PLOS Biology editors, the Academic Editor and the original reviewers.

You'll see that reviewer #1 is now enthusiastic about the study, and has no further requests. Reviewer #2 is also positive, but has one remaining request to perform some relatively minor analyses. Reviewer #3 also welcomes your re-framing of the paper, but has a number of substantial remaining requests, asking you to tone down some claims (point 3), flag a major limitation (point 2), and clarify some methodological details (points 1 and 4).

IMPORTANT: In addition to these comments from the reviewers, the Academic Editor has provided some guidance and some further requests, which can be found at the foot of this letter, below the reviews. Please also attend to these.

In light of the reviews, which you will find at the end of this email, we are pleased to offer you the opportunity to address the remaining points from the reviewers in a revision that we anticipate should not take you very long. We will then assess your revised manuscript and your response to the reviewers' comments with our Academic Editor aiming to avoid further rounds of peer-review, although we might need to consult with the reviewers, depending on the nature of the revisions.

In addition to these revisions, you may need to complete some formatting changes, which you will receive in a follow up email. A member of our team will be in touch with a set of requests shortly. If you do not receive a separate email within a few days, please assume that checks have been completed, and no additional changes are required.

We expect to receive your revised manuscript within 1 month. Please email us (plosbiology@plos.org) if you have any questions or concerns, or would like to request an extension.

At this stage, your manuscript remains formally under active consideration at our journal; please notify us by email if you do not intend to submit a revision so that we withdraw the manuscript.

**IMPORTANT - SUBMITTING YOUR REVISION**

Your revisions should address the specific points made by each reviewer. Please submit the following files along with your revised manuscript:

1. A 'Response to Reviewers' file - this should detail your responses to the editorial requests, present a point-by-point response to all of the reviewers' comments, and indicate the changes made to the manuscript.

*NOTE: In your point-by-point response to the reviewers, please provide the full context of each review. Do not selectively quote paragraphs or sentences to reply to. The entire set of reviewer comments should be present in full and each specific point should be responded to individually.

You should also cite any additional relevant literature that has been published since the original submission and mention any additional citations in your response.

2. In addition to a clean copy of the manuscript, please also upload a 'track-changes' version of your manuscript that specifies the edits made. This should be uploaded as a "Revised Article with Changes Highlighted " file type.

*Resubmission Checklist*

When you are ready to resubmit your revised manuscript, please refer to this resubmission checklist: https://plos.io/Biology_Checklist

To submit a revised version of your manuscript, please go to https://www.editorialmanager.com/pbiology/ and log in as an Author. Click the link labelled 'Submissions Needing Revision' where you will find your submission record.

Please make sure to read the following important policies and guidelines while preparing your revision:

*Published Peer Review*

Please note while forming your response, if your article is accepted, you may have the opportunity to make the peer review history publicly available. The record will include editor decision letters (with reviews) and your responses to reviewer comments. If eligible, we will contact you to opt in or out. Please see here for more details:

https://blogs.plos.org/plos/2019/05/plos-journals-now-open-for-published-peer-review/

*PLOS Data Policy*

Please note that as a condition of publication PLOS' data policy (http://journals.plos.org/plosbiology/s/data-availability) requires that you make available all data used to draw the conclusions arrived at in your manuscript. If you have not already done so, you must include any data used in your manuscript either in appropriate repositories, within the body of the manuscript, or as supporting information (N.B. this includes any numerical values that were used to generate graphs, histograms etc.). For an example see here: http://www.plosbiology.org/article/info%3Adoi%2F10.1371%2Fjournal.pbio.1001908#s5

*Blot and Gel Data Policy*

We require the original, uncropped and minimally adjusted images supporting all blot and gel results reported in an article's figures or Supporting Information files. We will require these files before a manuscript can be accepted so please prepare them now, if you have not already uploaded them. Please carefully read our guidelines for how to prepare and upload this data: https://journals.plos.org/plosbiology/s/figures#loc-blot-and-gel-reporting-requirements

*Protocols deposition*

To enhance the reproducibility of your results, we recommend that if applicable you deposit your laboratory protocols in protocols.io, where a protocol can be assigned its own identifier (DOI) such that it can be cited independently in the future. Additionally, PLOS ONE offers an option for publishing peer-reviewed Lab Protocol articles, which describe protocols hosted on protocols.io. Read more information on sharing protocols at https://plos.org/protocols?utm_medium=editorial-email&utm_source=authorletters&utm_campaign=protocols

Thank you again for your submission to our journal. We hope that our editorial process has been constructive thus far, and we welcome your feedback at any time. Please don't hesitate to contact us if you have any questions or comments.

Sincerely,

Roli

Roland Roberts, PhD

Senior Editor

PLOS Biology

rroberts@plos.org

----------------------------------------------------------------

REVIEWERS' COMMENTS:

Reviewer #1:

[identifies himself as Robert Feil]

The authors went through considerable efforts to address the points raised by this and the other reviewers. The manuscript now includes informative data on E12.5 embryos and, where needed, the authors added further replicates and technical control experiments. In addition, they improved the presentation of their data and turned down the conclusions about non-imprinted genes. they now also provide a balanced discussion that, importantly, highlights the limitations of the experimental systems used. Overall, this is an excellent revision, which considerably improved the value of this manuscript.

Reviewer #2:

The authors have done an excellent job substantially revising the paper. Most importantly, they moderated their claims of parental asymmetry beyond imprinting, conducted additional analyses for rigor, used more appropriate controls for the bipaternal offspring and clarified methods.

I do have one further request with respect to analysis: Figure 3H shows global DNA methylation levels across 5 organs in term WT and BP mice. Even though there are not many samples in each group, there could be greater analysis performed to try and understand where there the BP hypermethylation arises—the sequences can be separated by repetitive elements, regulatory elements, exons etc. These are standard analyses, which could be informative and useful. .

Reviewer #3:

[identifies himself as Gavin Kelsey]

This manuscript represents a substantial reframing of the previous submission "Functional imbalance in bi-paternal mice reveals postnatal consequences of parental genome asymmetry". The authors have pivoted away from their earlier interpretation that the physiological phenotype of 'bipaternal' mice was evidence for a contribution of the paternal genome beyond classical genomic imprinting. This change in emphasis is welcome. Instead, the authors frame the manuscript in terms of developmental and transcriptional variation, with the interpretation that the presence of both parental genome contributions provides developmental robustness, thereby helping to "constrain mammalian development within a relatively narrow range of trajectories". This reframing is bolstered by the addition of new samples in the postnatal tissue transcriptome analysis (increasing sample size from 2 to 4), as well as addition of transcriptome analysis of mid-gestation embryos. The latter, importantly, incorporates a new and more appropriate control group. The author's new interpretation has interesting parallels with observations that although parthenogenesis is observed in some non-mammalian vertebrates, success of uniparental reproduction is generally limited with variable outcomes.

As before, the study is characterised by the authors' experimental skills in elegant embryo manipulations, and achieving a live birth rate of 15% of the engineering biparental mice is a fantastic achievement. But there remain concerns about just how much the authors can conclude about phenotypic variability with their current experimental design.

I suggest that the authors should address the following points.

1. Some methodological details are still missing:

The main control group - the naturally conceived embryos and offspring - are referred to as WT. The authors should identify in the Methods which mouse strain these are and whether they are isogenic with the ES cells used (both the engineered BP-ESCs and WT-ESCs). The authors should also confirm the genetic background of the BP-ESCs and WT-ESCs. Please also specify what the WT-ESCs are. My assumption is that they are unmanipulated, diploid biparental ESCs, but please confirm (you could conceive of an experimental design in which the ideal control ESCs were diploid androgenetic, but these would not support development to the necessary stage).

2. Concern about the control groups:

The authors have responded to my comments and those of reviewer 1 by adding tetraploid complementation with WT ES cells as a control group, which they apply in a new section on embryonic outcomes (analysed at e12.5). This is an important improvement of the new manuscript. But it also sets up an inconsistency in the manuscript, in that WT ES constructs are seen as the ideal control in the embryonic analysis, whereas naturally conceived WT animals provide the only control group in all the postnatal analyses. The Sankey plot in Fig. S1C is important in this respect. The impression from this plot is that there is much greater concordance of gene expression states between the BP TC and ES (WT) TC groups than between either of the TC groups and the WT group. This seems logical, as the comparison is between unmanipulated, naturally conceived embryos (WT) and the two classes of ESC-derived embryos with tetraploid complementation. But it means that the control group for the postnatal analysis is not optimal.

3. Tendency to overstatement of results:

There are instances where results are rather cursorily or selectively described; for example:

"Consistent with this pattern, pairwise Euclidean distance analysis revealed significantly greater within-group distances among BP TC embryos than among WT embryos, whereas WT TC embryos showed no comparable increase relative to WT embryos (Fig. 2E)." But it is also the case in this figure that there is no significant difference in the Euclidean distance between the WT TC and BP TC groups, which is arguably the more relevant comparison.

"Differential expression analysis followed by gene set enrichment analysis identified alterations in

pathways related to core cellular and metabolic processes, including protein translation, complement activation, and glucose metabolism (Fig. 2F). Notably, pathway-level module scores also displayed

broader distributions among BP embryos than among WT embryos, indicating that transcriptional

states associated with these biological processes varied across individuals." But it is not clear that BP TC embryos show a greater range of values than the WT TC (labelled ES TC in the figure).

"WT TC embryos showed only a modest rightward shift relative to WT embryos, suggesting that tetraploid complementation introduces limited transcriptional variability, whereas the BP condition further increases this range (Fig. 2G)." Whereas there seems to be a major shift between WT and ES TC and a more modest difference between the ES TC and BP TC groups.

"Principal component analysis (PCA) showed that WT samples generally formed relatively compact

clusters within each tissue, whereas BP samples occupied wider regions of transcriptional space in

several organs (Fig. 3A, S3A)." It is not evident that there is greater dispersion of BP TC samples in most of the PCA plots in Fig. S3A.

Finally in this context, although the sample number for the postnatal analysis has been increased to 4, which is welcome, if the new emphasis of the study is transcriptional or phenotypic variation, it is not clear that this sample size is sufficient to make robust conclusions of variance.

4. Some experimental details are missing, which should be provided for clarity:

Fig. 1E: please specify how many litters these data derive from.

Fig. 2C: are the embryo length data from single or multiple litters per group? The point here being that readers should know whether the greater variation in length in the BP TC group is not a result of a technical or trivial factor, such as the WT embryos being from a single litter but the BP TC embryos from 2 or more litters that could have slight developmental asynchrony. It could be that the production of BP TC embryos is highly efficient, so that all embryos are from the same litter, but we need to know this.

Fig. 3I: what is the scale for the methylation plot? If the highlighted region is indeed the gDMR for Mcts2/H13, the expectation is that in the WT tissues it should be ~50% methylated.

One of the problems with this sort of plot is that where there are 'gaps' - for example, across much of the gDMR in the BP kidney - it is not clear whether this is because the sites are unmethylated or because there are missing values. Having a plot that also incorporates read number per position would eliminate that ambiguity.

Other corrections/comments

Consistency in labelling: the WT ESC tetraploid-complemented embryos are variously labelled as WT TC, ES TC and even ES 4N in the figures (e.g., Fig. 1, Fig. 2), and even differently within the same figure (Fig. S1). Similarly, the BP TC samples sometimes are referred to as BP 4N. Please use a consistent nomenclature for the samples.

COMMENTS FROM THE ACADEMIC EDITOR [lightly edited]:

Overall, I agree that the manuscript is much improved as noted by the reviewers. I also agree with reviewer #3 that much more details on methodology needs to be provided for a true biological interpretation/understanding of the phenomenon. As a reader I need to know if the results are biologically relevant or a phenomenon that results form the extensive manipulations that these cells/embryos undergo.

One thing I could not find in the revision is information about the sequencing and whether the new samples were sequenced separate from the original samples, or if the original samples were re-sequenced with the added observations. If the former, this alone introduces a lot of variability in the results.

Also, one thing that is difficult for me to see as a biologist is the inconsistent number of observations for the groups between the figures. Also, when reading the methodology, which is incredibly important to fully understand prior to accepting findings as having biological relevance, there is an overall lack of detail as to how many of any were done, collected, analyzed etc. For example, how many embryos were transferred in order to get the few analyzed? Complete transparency of methodology is imperative for the reader to be able to determine the strength of the results.

I agree with Reviewers #1 and #2 that this manuscript should be added to the body of literature becasue it represents an amazing feat, but I also agree with reviewer #3 about potential bias in biological interpretation resulting from the methods. Overall, I think the paper is much improved but authors must carefully consider and answer all of the reviewer #3's concerns and the point brought up by reviewer #2 prior to publication.

# Author comment: Author response to Decision Letter 2

**DOI:** 10.1371/journal.pbio.3003871.r005

18 May 2026

Attachment Submitted filename: Response Letter.docx

# Editor report: Decision Letter 2

**DOI:** 10.1371/journal.pbio.3003871.r006

**Authors:** Roland Roberts, Roland Roberts, Roland Roberts

28 May 2026

Dear Zhikun,

Thank you for your patience while we considered your revised manuscript "Developmental and physiological features of bipaternal mice lacking maternal genomic contribution" for publication as a Research Article at PLOS Biology. This revised version of your manuscript has been evaluated by the PLOS Biology editors and the Academic Editor.

Based on our Academic Editor's assessment of your revision, we are likely to accept this manuscript for publication, provided you satisfactorily address the following data and other policy-related requests.

IMPORTANT - please attend to the following:

a) We ideally need the Title to contain an active verb, and also to mention that the bipaternal mice were engineered. We suggest changing your Title to: "Engineered bipaternal mice reveal the consequences of life without a maternal genomic contribution"

b) I note that you say, of the ethical approval, "No specific approval number was issued; approval was granted by the named committee." Can you confirm that this is the case, and that no approval number was provided?

c) Please address my Data Policy requests below; specifically, we need you to supply the numerical values underlying Figs 1BEFH, 2CDEFGH, 3, 4ABCDEFG, 5ABCEG, S1ABCDEFG, S2, S3AB, S4AB, S5, S6, S7ABC, either as a supplementary data file or as a permanent DOI’d deposition.

d) Please cite the location of the data clearly in all relevant main and supplementary Figure legends, e.g. “The data underlying this Figure can be found in S1 Data” or “The data underlying this Figure can be found in https://zenodo.org/records/XXXXXXXX"; also update your Data Availability Statement accordingly.

e) We note that your Materials and Methods section, and the supplementary Figure legends are currently in the Supplement (Supplementary Information.docx) – please move this material to the main manuscript.

f) Please make any custom code available, either as a supplementary file or as part of your data deposition.

g) Please include the URLs of your funders in the Financial Disclosure statement.

As you address these items, please take this last chance to review your reference list to ensure that it is complete and correct. If you have cited papers that have been retracted, please include the rationale for doing so in the manuscript text, or remove these references and replace them with relevant current references. Any changes to the reference list should be mentioned in the cover letter that accompanies your revised manuscript.

In addition to these revisions, you may need to complete some formatting changes, which you will receive in a follow up email. A member of our team will be in touch with a set of requests shortly. If you do not receive a separate email within a few days, please assume that checks have been completed, and no additional changes are required.

We expect to receive your revised manuscript within two weeks.

To submit your revision, please go to https://www.editorialmanager.com/pbiology/ and log in as an Author. Click the link labelled 'Submissions Needing Revision' to find your submission record. Your revised submission must include the following:

- a cover letter that should detail your responses to any editorial requests, if applicable, and whether changes have been made to the reference list

- a Response to Reviewers file that provides a detailed response to the reviewers' comments (if applicable, if not applicable please do not delete your existing 'Response to Reviewers' file.)

- a track-changes file indicating any changes that you have made to the manuscript.

NOTE: If Supporting Information files are included with your article, note that these are not copyedited and will be published as they are submitted. Please ensure that these files are legible and of high quality (at least 300 dpi) in an easily accessible file format. For this reason, please be aware that any references listed in an SI file will not be indexed. For more information, see our Supporting Information guidelines:

https://journals.plos.org/plosbiology/s/supporting-information

*Published Peer Review History*

Please note that you may have the opportunity to make the peer review history publicly available. The record will include editor decision letters (with reviews) and your responses to reviewer comments. If eligible, we will contact you to opt in or out. Please see here for more details:

https://plos.org/published-peer-review-history/

*Press*

Should you, your institution's press office or the journal office choose to press release your paper, please ensure you have opted out of Early Article Posting on the submission form. We ask that you notify us as soon as possible if you or your institution is planning to press release the article.

*Protocols deposition*

To enhance the reproducibility of your results, we recommend that if applicable you deposit your laboratory protocols in protocols.io, where a protocol can be assigned its own identifier (DOI) such that it can be cited independently in the future. Additionally, PLOS ONE offers an option for publishing peer-reviewed Lab Protocol articles, which describe protocols hosted on protocols.io. Read more information on sharing protocols at https://plos.org/protocols?utm_medium=editorial-email&utm_source=authorletters&utm_campaign=protocols

Please do not hesitate to contact me should you have any questions.

Sincerely,

Roli

Roland Roberts, PhD

Senior Editor

rroberts@plos.org

PLOS Biology

------------------------------------------------------------------------

ETHICS STATEMENT:

Thanks for providing the name of the IACUC/ethics committee that reviewed and approved the animal care and use protocol/permit/project license. Please also include an approval number.

------------------------------------------------------------------------

DATA POLICY:

You may be aware of the PLOS Data Policy, which requires that all data be made available without restriction: http://journals.plos.org/plosbiology/s/data-availability. For more information, please also see this editorial: http://dx.doi.org/10.1371/journal.pbio.1001797

Note that we do not require all raw data. Rather, we ask that all individual quantitative observations that underlie the data summarized in the figures and results of your paper be made available in one of the following forms:

1) Supplementary files (e.g., excel). Please ensure that all data files are uploaded as 'Supporting Information' and are invariably referred to (in the manuscript, figure legends, and the Description field when uploading your files) using the following format verbatim: S1 Data, S2 Data, etc. Multiple panels of a single or even several figures can be included as multiple sheets in one excel file that is saved using exactly the following convention: S1_Data.xlsx (using an underscore).

2) Deposition in a publicly available repository. Please also provide the accession code or a reviewer link so that we may view your data before publication.

Regardless of the method selected, please ensure that you provide the individual numerical values that underlie the summary data displayed in the following figure panels as they are essential for readers to assess your analysis and to reproduce it: Figs 1BEFH, 2CDEFGH, 3, 4ABCDEFG, 5ABCEG, S1ABCDEFG, S2, S3AB, S4AB, S5, S6, S7ABC. NOTE: the numerical data provided should include all replicates AND the way in which the plotted mean and errors were derived (it should not present only the mean/average values).

Please also ensure that figure legends in your manuscript include information on where the underlying data can be found, and ensure your supplemental data file/s has a legend.

Please ensure that your Data Statement in the submission system accurately describes where your data can be found.

------------------------------------------------------------------------

CODE POLICY

Per journal policy, if you have generated any custom code during the course of this investigation, please make it available without restrictions. Please ensure that the code is sufficiently well documented and reusable, and that your Data Statement in the Editorial Manager submission system accurately describes where your code can be found. More information on our Code Policy, what and how to share can be found here: https://journals.plos.org/plosbiology/s/code-availability

Please note that we cannot accept sole deposition of code in GitHub, as this could be changed after publication. However, you can archive this version of your publicly available GitHub code to Zenodo. Once you do this, it will generate a DOI number, which you will need to provide in the Data Accessibility Statement (you are welcome to also provide the GitHub access information). See the process for doing this here: https://docs.github.com/en/repositories/archiving-a-github-repository/referencing-and-citing-content

------------------------------------------------------------------------

BLOT AND GEL REPORTING REQUIREMENTS:

We require the original, uncropped and minimally adjusted images supporting all blot and gel results reported in an article's figures or Supporting Information files. We will require these files before a manuscript can be accepted so please prepare and upload them now. Please carefully read our guidelines for how to prepare and upload this data: https://journals.plos.org/plosbiology/s/figures#loc-blot-and-gel-reporting-requirements

------------------------------------------------------------------------

DATA NOT SHOWN?

- Please note that per journal policy, we do not allow the mention of "data not shown", "personal communication", "manuscript in preparation" or other references to data that is not publicly available or contained within this manuscript. Please either remove mention of these data or provide figures presenting the results and the data underlying the figure(s).

------------------------------------------------------------------------

# Author comment: Author response to Decision Letter 3

**DOI:** 10.1371/journal.pbio.3003871.r007

6 Jun 2026

Attachment Submitted filename: Response_Letter_auresp_3.docx

# Editor report: Decision Letter 3

**DOI:** 10.1371/journal.pbio.3003871.r008

**Authors:** Roland Roberts, Roland Roberts, Roland Roberts, Roland Roberts

8 Jun 2026

Dear Zhikun,

Thank you for the submission of your revised Research Article "Engineered bipaternal mice reveal the consequences of life without a maternal genomic contribution" for publication in PLOS Biology. On behalf of my colleagues and the Academic Editor, Rocio Rivera, I'm pleased to say that we can in principle accept your manuscript for publication, provided you address any remaining formatting and reporting issues. These will be detailed in an email you should receive within 2-3 business days from our colleagues in the journal operations team; no action is required from you until then. Please note that we will not be able to formally accept your manuscript and schedule it for publication until you have completed any requested changes.

Please take a minute to log into Editorial Manager at http://www.editorialmanager.com/pbiology/, click the "Update My Information" link at the top of the page, and update your user information to ensure an efficient production process.

PRESS: We frequently collaborate with press offices. If your institution or institutions have a press office, please notify them about your upcoming paper at this point, to enable them to help maximise its impact. If the press office is planning to promote your findings, we would be grateful if they could coordinate with biologypress@plos.org. If you have previously opted in to the early version process, we ask that you notify us immediately of any press plans so that we may opt out on your behalf.

We also ask that you take this opportunity to read our Embargo Policy regarding the discussion, promotion and media coverage of work that is yet to be published by PLOS. As your manuscript is not yet published, it is bound by the conditions of our Embargo Policy. Please be aware that this policy is in place both to ensure that any press coverage of your article is fully substantiated and to provide a direct link between such coverage and the published work. For full details of our Embargo Policy, please visit http://www.plos.org/about/media-inquiries/embargo-policy/.

Thank you again for choosing PLOS Biology for publication and supporting Open Access publishing. We look forward to publishing your study.

Sincerely,

Roli

Roland G Roberts, PhD, PhD

Senior Editor

PLOS Biology

rroberts@plos.org
